# Global, regional, national epidemiology and trends of Parkinson’s disease from 1990 to 2021: findings from the Global Burden of Disease Study 2021

**DOI:** 10.3389/fnagi.2024.1498756

**Published:** 2025-01-10

**Authors:** Yuanrong Luo, Lichun Qiao, Miaoqian Li, Xinyue Wen, Wenbin Zhang, Xianwen Li

**Affiliations:** ^1^Department of Anesthesiology, Affiliated Nanjing Brain Hospital of Nanjing Medical University, Nanjing, China; ^2^School of Public Health, Xi’an Jiaotong University Health Science Center, Xi’an, China; ^3^Department of Functional Neurosurgery, Affiliated Nanjing Brain Hospital of Nanjing Medical University, Nanjing, China; ^4^School of Nursing, Nanjing Medical University, Nanjing, China

**Keywords:** Parkinson’s disease, Global Burden of Disease Study, disability-adjusted life years, DALYs, population aging

## Abstract

**Aims:**

In light of the escalating global incidence of Parkinson’s disease and the dearth of therapeutic interventions that can alter the disease’s course, there exists an urgent necessity to comprehensively elucidate and quantify the disease’s global burden.

**Methods:**

This study analyzed the incidence, prevalence, and disability-adjusted life years (DALYs) of Parkinson’s disease at global, regional, and national levels based on the Global Burden of Disease Study 2021. Bayesian age-period cohort (BAPC) analysis was used to predict the burden in Parkinson’s disease from 2022 to 2035.

**Results:**

In 2021, 11.77 million people worldwide had Parkinson’s disease. Age-standardized rates of incidence, prevalence, and DALYs increased to 15.63/100,000, 138.63/100,000, and 89.59/100,000. The burden of Parkinson’s disease were higher in males than in females, and showed an increase and then a slight decrease with age. The disease burden was highest in East Asia. BAPC projection showed an increase in all metrics by 2035 except for a slight decrease in the age-standardized DALYs rates.

**Conclusion:**

The global burden of Parkinson’s disease has risen over the past 32 years, and there is a need to focus on key populations, as well as to improve health policies to prevent and treat Parkinson’s disease.

## 1 Introduction

Parkinson’s disease is a progressive neurodegenerative disorder with high prevalence in middle-aged and elder people, primarily characterized by motor symptoms such as tremors, rigidity, and bradykinesia, which severely affects the quality of life (Xu et al., 2024; Sun et al., 2024; Aarsland et al., 1999). It was first described by James Parkinson in 1817 and is the most prevalent movement disorder and second most common degenerative disease of the central nervous system after Alzheimer’s disease (Sun et al., 2024; Samii et al., 2004). The etiology of Parkinson’s disease remains predominantly elusive. However, it is increasingly recognized that a confluence of environmental and genetic factors can precipitate the disease, particularly in individuals with a hereditary predisposition. Epidemiological studies have identified exposure to pesticides, herbicides, and industrial chemicals as significant risk factors, with heightened vulnerability observed among agricultural and industrial laborers (Tysnes and Storstein, 2017). Furthermore, the GBA gene, which encodes the lysosomal enzyme glucocerebrosidase (GCase) and is pivotal in glycosphingolipid metabolism, has been associated with Parkinson’s disease pathology. Mutations in the GBA gene are present in an estimated 5–15% of Parkinson’s disease patients, underscoring its role as a substantial genetic component in the disease’s pathogenesis (Delamarre and Meissner, 2017). This disease not only affects individuals but also imposes significant socio-economic challenges. It has become a major public health problem worldwide with the increasing degree of population ageing.

The Global Burden of Disease, Injury and Risk Factors Study (GBD) estimated that 6.1 million individuals worldwide had Parkinson’s disease in 2016, up from just 2.5 million in 1990, and that the age-standardized prevalence rate (ASPR) increased by 21.7% over the same period (GBD 2016 Parkinson’s Disease Collaborators, 2018). Notably, the number of patients was approximately 8.5 million in 2019, an increase of 39.34% compared to 2016 (GBD 2019 Diseases and Injuries Collaborators, 2020). Moreover, according to the data from the World Health Organisation in 2023, disability-adjusted life years (DALYs) due to Parkinson’s disease have increased by 81% since 2000, and deaths have increased by more than 100% (World Health Organization, 2023). From a global perspective, the burden of Parkinson’s disease has been increasing, reflecting an ageing population and advancements in diagnostic capabilities. Therefore, understanding the epidemiology and trends of Parkinson’s disease is crucial for public health planning, resource allocation and the development of targeted interventions.

In this study, we presented a detailed examination of the epidemiological trends and burden of Parkinson’s disease across different regions, countries, Socio-demographic Index (SDI), sexes and age groups from 1990 to 2021, emphasizing the disparities that exist in the burden of disease. In addition, trends in Parkinson’s disease burden were projected for the next decade or so. Such disparities and timely projections highlight the importance of developing effective public health responses.

## 2 Methods

### 2.1 Study data source

The Global Burden of Disease Study 2021 estimated the incidence, prevalence, mortality, risk factors, and DALYs of 371 diseases in 204 countries and territories from 1990 to 2021 (GBD 2021 Diseases and Injuries Collaborators, 2024). The data collection involved a systematic assessment derived from censuses, household surveys, vital registration and population statistics, disease registries, disease notifications, health service utilization, air pollution monitoring devices, satellite imagery, and other sources (GBD 2021 Causes of Death Collaborators, 2024). These data were obtained by downloading from the Global Health Data Exchange,^1^ by the specified requirements. This study extracted and analyzed data on the global burden of disease for Parkinson’s disease from 1990 to 2021.

### 2.2 Statistical analysis

This study described the burden of Parkinson’s disease from 1990 to 2021 in 204 countries, 21 regions, and 5 levels of the SDI, using the number and rate of incidence, prevalence, DALYs, and their 95% uncertain interval (UI), and stratified by different sexes. We delineated the evolution of the global burden of Parkinson’s from 1990 to 2021. To capture the trends in the disease burden attributable to Parkinson’s, we employed linear regression analysis to compute the estimated annual percentage change (EAPC) in the global age-standardized rates (ASRs) (Sun et al., 2024). The Spearman’s correlation coefficient was used to evaluate the relationship between SDI and ASRs of Parkinson’s disease at the global, regional, and national levels. Bayesian age-period-cohort (BAPC) model integrated nested Laplace approximations to predict the burden of Parkinson’s disease from 2022 to 2035 (Jürgens et al., 2014). Statistical analysis and visualization were performed using the R software program (version 4.1.2) and JD GBDR (V2.24, Jingding Medical Technology Co., Ltd.). *p*-value <0.05 was considered statistically significant.

## 3 Results

### 3.1 Global level

There were 0.13 (95% UI: 0.12–0.15) million incident cases with Parkinson’s disease in 2021, which increased by 220.07% (95% UI: 210.02–229.15%) compared to 1990. The incident cases for males [0.76 (95% UI: 0.68–0.85) million] in 2021 were higher than for females [0.52 (95% UI: 0.51–0.63) million], and the increase in the former was also higher than in the latter over past 32 years (Supplementary Table S1). Globally, the age-standardized incidence rate (ASIR) was 15.63/100,000 (95% UI: 14.03/100,000–17.39/100,000) in 2021 with an EAPC of 1.07 (95% CI: 1.07–1.11). For males, the ASIR was 19.72/100,000 (95% UI: 17.69/100,000–21.88/100,000), which was approximately 1.60 times higher than for females in 2021. The EAPCs for males and females were 1.11 (95% CI: 1.09–1.13) and 0.93 (95% CI: 0.91–0.95), respectively (Supplementary Table S2).

The prevalent cases of Parkinson’s disease in 2021 were 11.77 (95% UI: 10.44–13.42) million, increased by 273.76% (95% UI: 260.22–287.26%) from 1990. For different sexes, the prevalent cases were 6.44 (95% UI: 5.67–7.40) million and 5.33 (95% UI: 4.75–6.04) million for males and females, respectively, with the increase being higher in the former (Supplementary Table S1). The age-standardized prevalence rate (ASPR) was 138.63/100,000 (95% UI: 123.06/100,000–157.62/100,000) with an EAPC of 1.52 (95% CI: 1.49–1.54) in 2021. Over the past 32 years, the ASPR increased for both sexes, rising to 168.24/100,000 (95% UI: 148.41/100,000–191.71/100,000) for males and 114.47/100,000 (95% UI: 102.05/100,000–129.78/100,000) for females (Supplementary Table S2).

The DALYs number of Parkinson’s disease in 2021 was 7.47 (95% UI: 6.74–8.14) million, which increased by 161.80% (95% UI: 146.07–177.39%). From 1990 to 2021, the DALYs number increased by 179.51% (95% UI: 157.02–200.85%) for males and 141.37% (95% UI: 123.73–160.53%) for females (Supplementary Table S1). Globally, the age-standardized death rate (ASDR) was 89.59/100,000 (95% UI: 80.69/100,000–97.50/100,000) in 2021, with an EAPC of 0.32 (95% CI: 0.28–0.36). Moreover, the ASDR was 117.47/100,000 (95% UI: 106.15/100,000–128.88/100,000) for males and 68.56/100,000 (95% UI: 60.90/100,000–75.76/100,000) for females in 2021, respectively (Supplementary Table S2).

### 3.2 Regional level

The incident cases, prevalent cases, and DALYs numbers have increased in all regions over the past 32 years. In 2021, the top three regions with the incident cases, prevalent cases and DALYs numbers were East Asia, South Asia, and Western Europe, with the East Asia having the highest burdens. The incident and prevalent cases of Parkinson’s disease in East Asia were 0.52 (95% UI: 0.45–0.61) million and 5.21 (95% UI: 4.42–6.18) million in 2021, which was a significant increase of 453.97% (95% UI: 419.00–489.82%) and 675.27% (95% UI: 619.23–729.36%) compared to 1990. Moreover, the DALYs number was 7.47 (95% UI: 6.74–8.14) million, an increase of 161.80% (95% UI: 146.07–177.39%) from 1990 to 2021 (Supplementary Table S1).

The top three regions with ASIR were Andean Latin America, East Asia, and Western Europe, with East Asia having the highest ranking at 24.16/100,000 (95% UI: 20.61/100,000–28.00/100,000) in 2021. The top three regions with ASPR were Eastern Europe, Western Europe, and Western Sub-Saharan Africa. Western Europe had the highest ASPR of 7113.44/100,000 (95% UI: 6407.11/100,000–7867.10/100,000), with an EAPC of 0.67 (95% CI: 0.55–0.79). Furthermore, the top three regions with ASDR were Andean Latin America, East Asia, and High-income North America. East Asia had the highest ASDR of 107.68/100,000 (95% UI: 91.45/100,000–124.81/100,000) in 2021 (Supplementary Table S2).

### 3.3 National level

China, India, and United States of America consistently ranked among the top three countries in terms of incidence cases, prevalence cases, and DALYs, with China leading in all these metrics between 1990 and 2021. Among the 204 countries and regions analyzed, the State of Qatar and the United Arab Emirates stood out for being in the top three in terms of number of changes in incidence, prevalence, and DALYs. Specifically, Qatar experienced an increase of 10.49% (95% UI: 9.31–11.91) in incidence cases, 11.15% (95% UI: 9.62–12.95) in prevalence cases, and 3.98% (95% UI: 2.85–5.62) in DALYs. Meanwhile, the United Arab Emirates experienced an increase of 10.37% (95% UI: 9.06–12.13) in incidence cases, 12.43% (95% UI: 10.60–15.12) in prevalence cases, and 4.43% (95% UI: 3.30–6.51) in DALYs (Supplementary Table S3).

**Figure 1:**
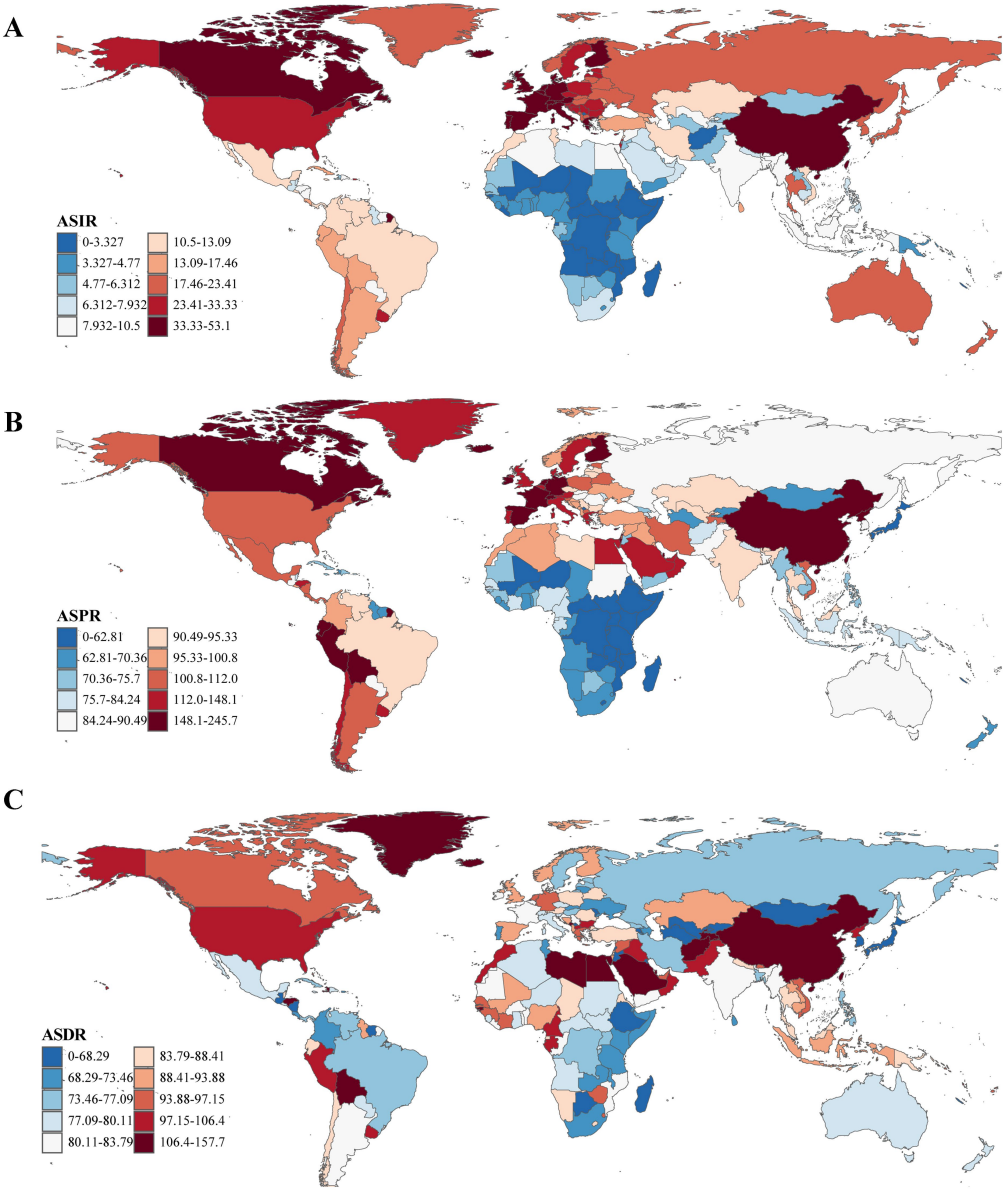
The ASRs (per 100,000 population) of Parkinson’s disease at the national level in 2021. **(A)** ASIR. **(B)** ASPR. **(C)** ASDR. ASRs, age-standardized rates; ASIR, age-standardized incidence rate; ASPR, age-standardized prevalence rate; ASDR, age-standardized disability-adjusted life years rate.

**Figure 2:**
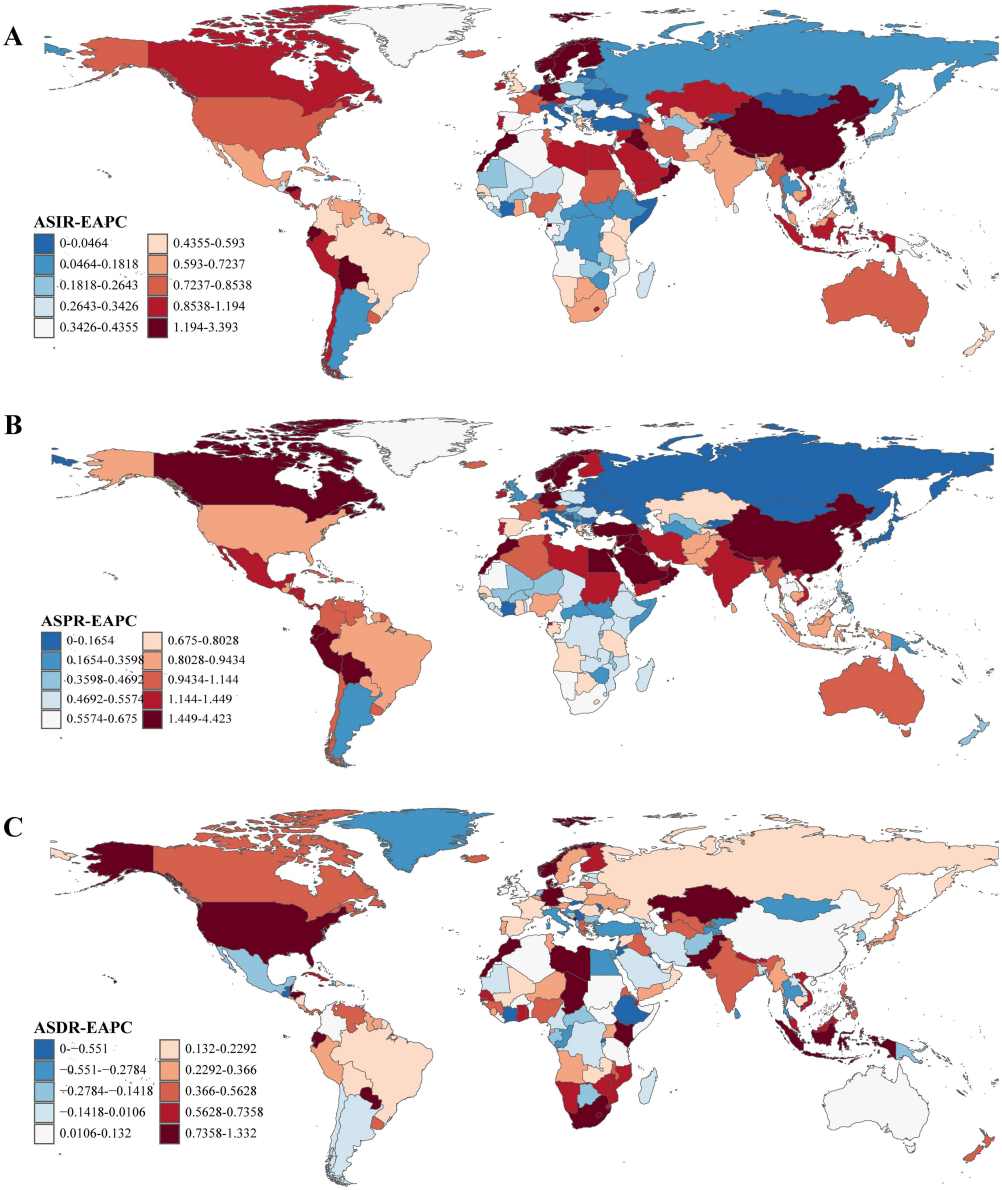
The EAPCs of ASRs of Parkinson’s disease at the national level from 1990 to 2021. **(A)** EAPC of ASIR. **(B)** EAPC of ASPR. **(C)** EAPC of ASDR. EAPC, estimated annual percentage change; ASRs, age-standardized rates; ASIR, age-standardized incidence rate; ASPR, age-standardized prevalence rate; ASDR, age-standardized disability-adjusted life years rate.

From 1990 to 2021, ASRs increased in the vast majority of countries and regions. In 1990, Israel [ASPR: [164.48/100,000 (95% UI: 138.47/100,000–199.54/100,000)] and ASIR: [18.45/100,000 (95% UI: 16.07/100,000–21.73/100,000)] and the Netherlands [ASPR: [162.18/100,000 (95% UI: 143.17/100,000–181.79/100,000)] and ASIR: [18.78/100,000 (95% UI: 16.77/100,000–20.45/100,000)] recorded the highest ASPR and ASIR, respectively. By 2021, China had emerged as the leading country in both metrics ASPR: [245.73/100,000 (95% UI: 208.28/100,000–289.24/100,000)] and ASIR: [24.34/100,000 (95% UI: 20.67/100,000–28.30/100,000)]. Notably, the countries and regions with the highest EAPCs in ASPR and ASIR were identical; however, the top three for ASDR differed, with Cyprus [−2.55 (95% UI: −2.77 to −2.32)], Qatar [−1.92, (95% UI: −2.46 to −1.37)], and Lesotho [1.67, 95% UI: (1.40–1.94)] leading the rankings (Supplementary Table S4 and Figures 1, 2).

### 3.4 SDI quintile level

The incident cases, prevalent cases, and DALYs number of Parkinson’s disease increased from 1990 to 2021 at different SDI quintiles, with the highest increases in the middle SDI quintile at 349.51% (95% UI: 331.95–368.99), 474.92% (95% UI: 444.99–503.89), and 215.92% (95% UI: 185.47–248.93), respectively. In 2021, the cases and numbers were in descending order middle, high-middle, high, low-middle, and low SDI quintiles (Supplementary Table S1). Furthermore, the ASRs also increased to varying degrees over the past 32 years. High-middle SDI quintile had the highest ASRs of 18.49/100,000 (95% UI: 16.31/100,000–21.03/100,000), 173.40/100,000 (95% UI: 151.36/100,000–200.71/100,000), and 94.16/100,000 (95% UI: 84.06/100,000–104.78/100,000) in 2021 (Supplementary Table S2).

**Figure 3:**
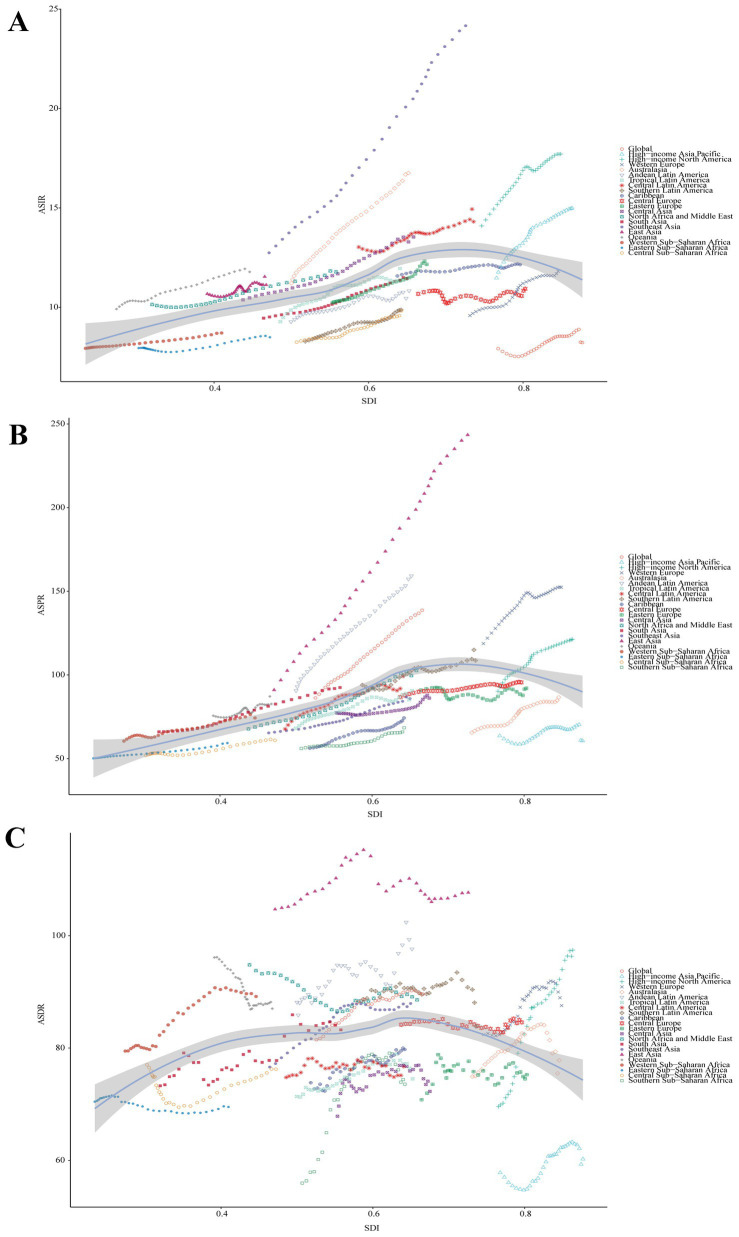
Correlation between SDI and ASRs of Parkinson’s disease at the regional level. **(A)** ASIR (*r* = 0.4219, *p* = 0.000×10^0^). **(B)** ASPR (*r* = 0.5337, *p* = 0.000×10^0^). **(C)** ASDR (*r* = 0.1041, *p* = 5.721×10^−3^. SDI, Socio-demographic Index; ASIR, age-standardized incidence rate; ASPR, age-standardized prevalence rate; ASDR, age-standardized disability-adjusted life years rate.

**Figure 4:**
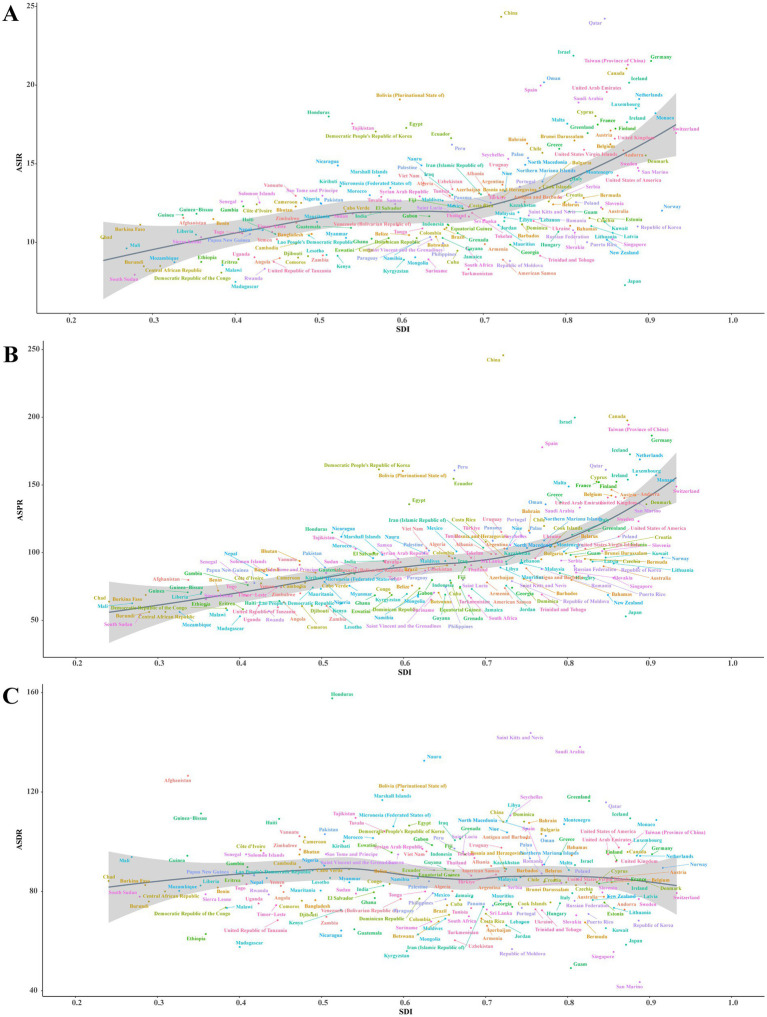
Correlation between SDI and ASRs of Parkinson’s disease at the national level in 2021. **(A)** ASIR (*r* = 0.5098, *p* = 0.000 × 10^0^). **(B)** ASPR (*r* = 0.6689, *p* = 0.000 × 10^0^). **(C)** ASDR (*r* = 0.0009, *p* = 9.902 × 10^−1^). Each colored dot above represents a country, and the line represents the average expected value based on the SDI and ASRs in 204 countries and territories. SDI, Socio-demographic Index; ASRs, age-standardized rates; ASIR, age-standardized incidence rate; ASPR, age-standardized prevalence rate; ASDR, age-standardized disability-adjusted life years rate.

At the regional level, it was observed that ASRs for Parkinson’s disease increased and then decreased as the SDI rose higher (Figure 3). The ASIR estimates in Oceania, North Africa and Middle East, East Asia, Southeast Asia, Australasia, Central Latin America, and high-income North America exceeded expectations. The ASPR estimates in Oceania, East Asia, Andean Latin America, and Western Europe exceeded expectations. Moreover, the ASPR estimates in Western Sub-Saharan Africa, Oceania, North Africa and Middle East, Andean Latin America, Southern Latin America, and East Asia exceeded expectations. At the national level, ASIR or ASPR was positively correlated with SDI in 204 countries and territories in 2021, suggesting that higher levels of SDI were associated with higher ASIR and ASPR for Parkinson’s disease (Figures 4A,B). While ASDR did not change significantly with increasing SDI (Figure 4C).

### 3.5 Burden based on age and sex

**Figure 5:**
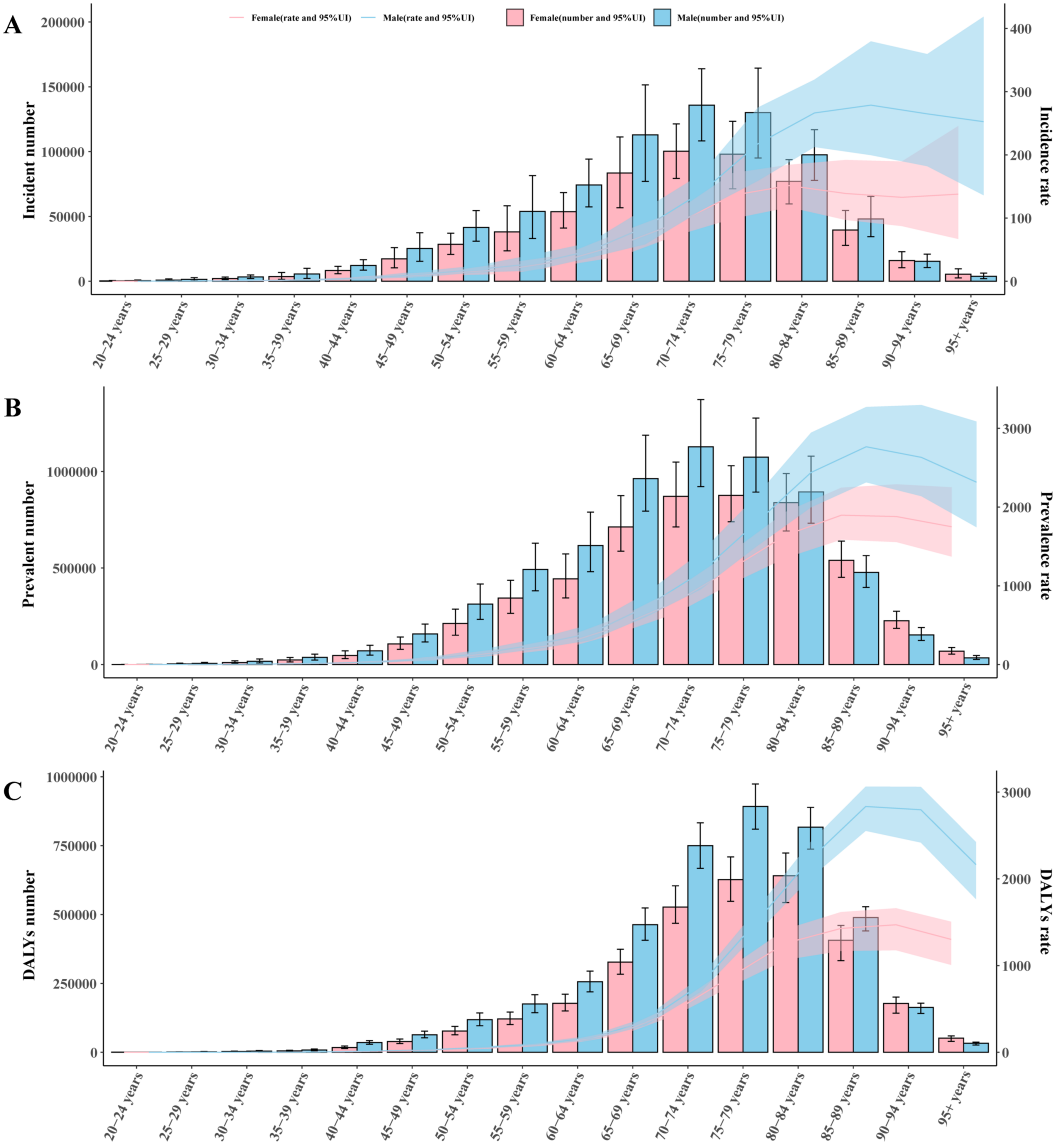
The global disease burden of Parkinson’s disease by age and sex in 2021. **(A)** Incident case and incidence rate. **(B)** Prevalent cases and prevalence rate. **(C)** DALYs number and DALYs rate. Error bars and shadow bands indicate 95% uncertainty intervals. DALYs, disability-adjusted life years.

Globally, the burden of Parkinson’s disease varies between sexes and ages. In 2021, the number and rate of incidence, prevalence and DALYs all showed a trend of increasing and then decreasing with age. In the 90–94 age group, the incidence number in females exceeded that of males. The highest incidence rates for males [278.68/100,000 (95% UI: 199.57/100,000–379.59/100,000)] and females [151.39/100,000 (95% UI: 17.20/100,000–184.31/100,000)] were found in the 85–89 and 80–84 age groups, respectively (Figure 5A). As for prevalence number, females exceeded males in the 85–89 age group. The highest prevalence rates for males [2766.09/100,000 (95% UI: 2314.08/100,000–3271.54/100,000)] and females [1897.02/100,000 (95% UI: 1586.83/100,000–2246.53/100,000)] were both found in the 85–89 age groups (Figure 5B). In addition, the DALYs number in females exceeded that of males was also in the 90–94 age group. The highest DALYs rates for males [2834.37/100,000 (95% UI: 2553.24/100,000–3064.28/100,000)] and females [1470.62/100,000 (95% UI: 1176.69/100,000–1661.83/100,000)] were found in the 85–89 and 90–94 age groups (Figure 5C).

**Figure 6:**
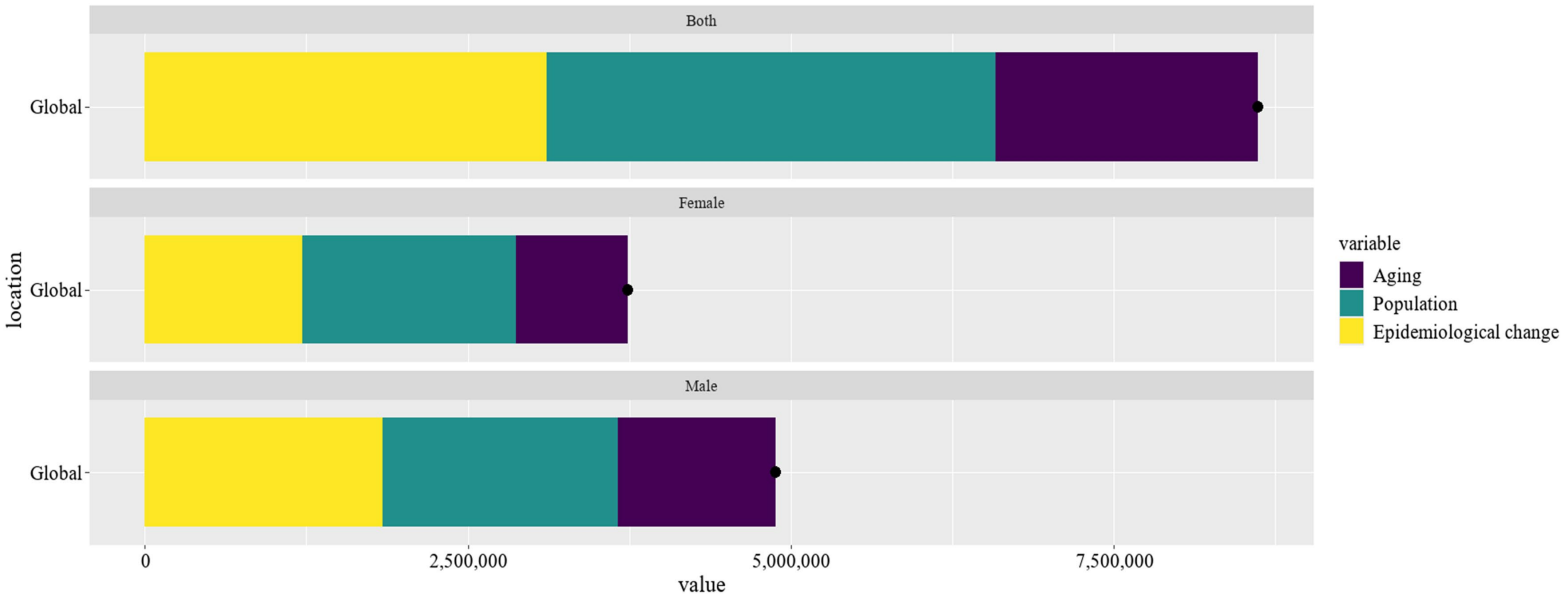
Evaluate the effects of population growth, aging, and epidemiological shifts on Parkinson’s disease prevalence from 1990 to 2021.

The decomposition analysis highlighted the multifaceted impacts of aging, population growth, and epidemiological changes on Parkinson’s disease prevalence (Figure 6). Aging contributed significantly to the prevalence of Parkinson’s disease, with 1218878.1 cases (24.98%) in males and 866391.05 cases (23.17%) in females, giving a cumulative global prevalence of 2034883.6 cases (23.61%). The substantial proportion of aging in Parkinson’s disease prevalence underscores the escalating disease burden due to the aging global population. Population growth had the greatest impact on the prevalence of Parkinson’s disease in females with 1651078.5 cases (44.16%) as compared to 1821435.52 cases (37.33%) in males and a global prevalence of 3475747.84 cases (40.33%). The pronounced effect of population growth on Parkinson’s disease prevalence highlighted the necessity for public health strategies to address the increasing number of cases. Epidemiological changes accounted for 1839358.75 cases in males (37.69%) and 1221735.5 cases in females (32.67%), totaling 3108245.98 cases (36.06%) globally. The contribution of epidemiological changes to Parkinson’s disease suggested that factors beyond aging and population growth, such as environmental exposures and lifestyle modifications, play a crucial role in the disease’s dynamics.

### 3.6 Projection burden from 2022 to 2035

**Figure 7:**
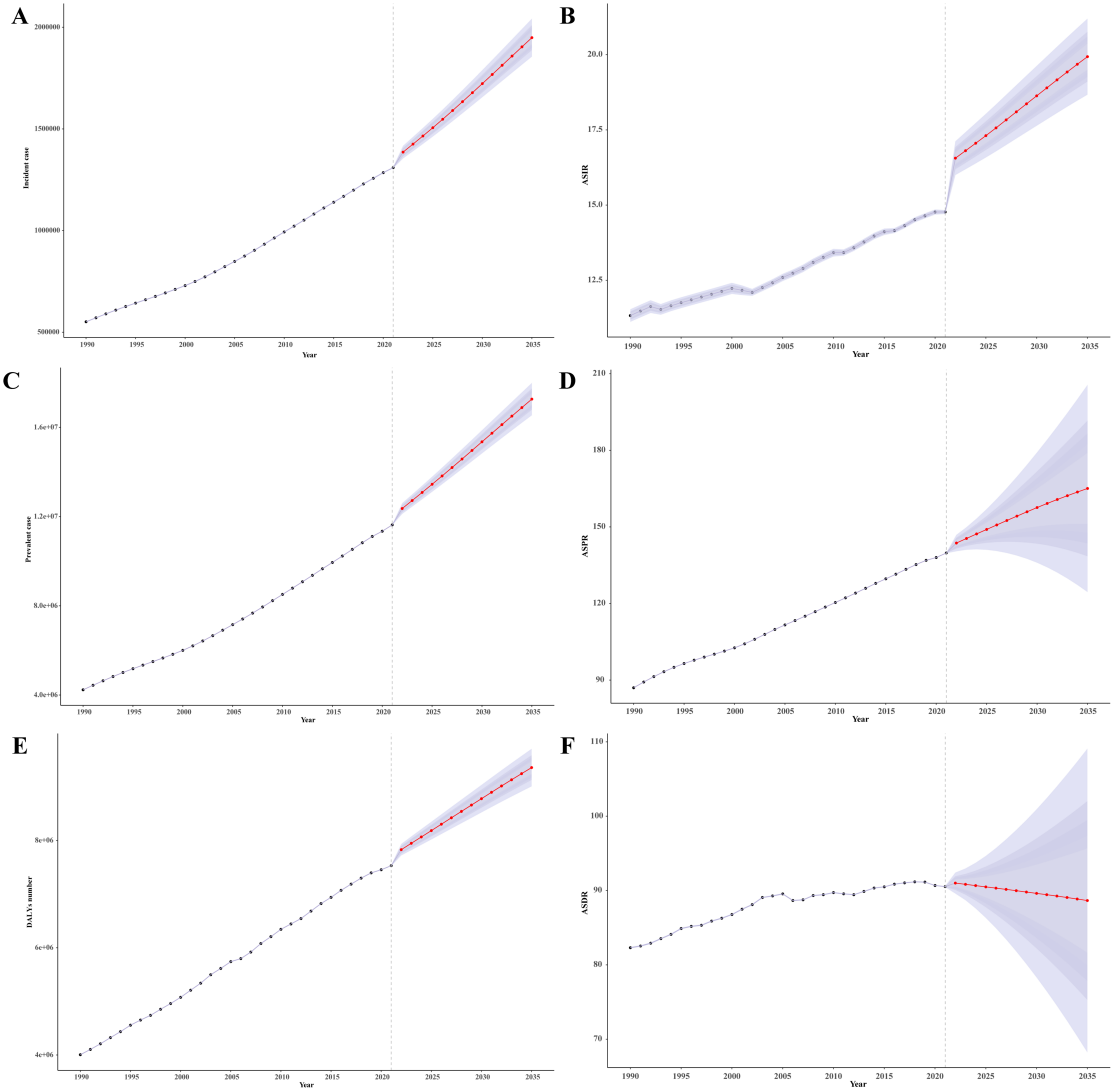
The projection of burden of Parkinson’s disease globally from 2022 to 2035. **(A)** Incident case. **(B)** ASIR. **(C)** Prevalent case. **(D)** ASIR. **(E)** DALYs number. **(F)** ASIR. DALYs, disability-adjusted life years; ASRs, age-standardized rates; ASPR, age-standardized prevalence rate; ASDR, age-standardized disability-adjusted life years rate.

We also projected the burden of Parkinson’s disease from 2022 to 2035 using the BAPC model. Over the next 14 years, the incident cases, prevalent cases, and DALYs number would increase from 1309567.02 (95% UI: 1306412.32–1312721.72), 11627666.63 (95% UI: 11618229.25–11637104.01), and 7530675.63 (95% UI: 7523047.31–7538303.95) in 2021 to 1948971.16 (95% UI: 1855771.49–2042170.83), 17268157.75 (95% UI: 16545461.71–17990853.8), and 9359677.37 (95% UI: 9010307.01–9709047.72) in 2035 (Figures 7A–C). Furthermore, the ASIR and ASPR for Parkinson’s disease would increase from [14.62/100,000 (95% UI:14.58/100,000–14,65/100,000)] and [139.79/100,000 (95% UI: 139.71/100,000–139.87/100,000)] in 2021 to [20.20/100,000 (95% UI: 18.58/100,000–21.81/100,000)] and [165.05/100,000 (95% UI: 124.49/100,000–205.62/100,000)] in 2035, respectively (Figures 7D,E). While the ASDR would decrease slightly, from [90.53/100,000 (95% UI: 90.47/100,000–90.57/100,000)] in 2021 to [88.66/100,000 (95% UI: 68.23/100,000–102.01/100,000)] in 2035 (Figure 7F).

## 4 Discussion

This study provides a comprehensive analysis of the global burden and epidemiological trends of Parkinson’s disease from 1990 to 2021, emphasizing significant regional and socio-economic differences. Our findings indicate a substantial increase in the global prevalence and incidence of Parkinson’s disease over the past two decades (Ben-Shlomo et al., 2024), with the most pronounced rises observed in countries with middle to high SDI. This upward trend is likely driven by factors such as population aging, changes in lifestyle, and environmental influences associated with urbanization and industrialization (Zhang et al., 2022; Wang et al., 2015; Zhu et al., 2024), particularly in rapidly developing regions (GBD 2016 Parkinson’s Disease Collaborators, 2018). Studies have demonstrated that in regions experiencing rapid urbanization, the incidence of Parkinson’s disease is heightened by environmental pollutants such as particulate matter, naphtha and its derivatives, occupational exposures, as well as the utilization of pesticides and insecticides (Aravindan et al., 2024).

From the perspective of ASIR, there has been a 220.07% increase globally from 1990 to 2021. Notably, in regions with middle to high SDI, the ASIR reached 18.49/100,000 in 2021, significantly higher than the 10.21/100,000 observed in low SDI regions. This disparity underscores the profound impact of socio-economic conditions on the burden of Parkinson’s disease. In higher SDI regions, advanced healthcare systems and diagnostic technologies contribute to earlier and more accurate diagnoses, whereas lower SDI countries may experience underdiagnosis and reporting issues due to limited medical resources, leading to an underestimation of true prevalence. It is crucial to recognize that the majority of Parkinson’s disease patients do not succumb directly to the disease; instead, they die from complications arising from Parkinson’s disease, such as pulmonary infections (Xu et al., 2014). This insight is pivotal for grasping the extended implications of Parkinson’s disease on mortality rates and underscores the imperative for holistic management approaches that anticipate and mitigate these complications. Additionally, the COVID-19 pandemic’s strain on healthcare resources and the rise in mortality rates may have further impacted Parkinson’s disease outcomes, global life expectancy at birth declined by 1.6 years (1.0–2.2) between 2019 and 2021, age-standardised mortality rates declined between 1950 and 2019 [a 62.8% (95% UI 60.5–65.1) decline], and increased during the COVID-19 pandemic period [2020–2021; 5.1% (0.9–9.6) increase] (GBD 2021 Demographics Collaborators, 2024).

In China, the burden of Parkinson’s disease has notably increased over recent decades. In 2021, the ASPR in China was 245.73/100,000, and the ASIR was 24.34/100,000. This increase is linked to China’s large population, escalating aging population, and significant advancements in diagnostic capabilities. However, many patients in China face delays in diagnosis, with most waiting over a year and 17% waiting more than 3 years. This highlights the need for improvements in early screening and diagnostic processes.

Globally, Parkinson’s disease burden shows significant variations by gender and age. Data from 2021 reveals that the incidence of Parkinson’s disease increases with age, but among individuals aged 75–79, the incidence in men is lower than in those aged 70–74. Overall, men have a 1.6-fold higher incidence rate compared to women, with men also showing higher ASPR and ASDR. This discrepancy may be related to higher smoking rates, greater intake of sugars and animal fats among men (World Health Organization, 2019), and possibly the neuroprotective effects of estrogen and other sex-linked genetic factors in women (Boulos et al., 2019; Terrin et al., 2023; Chen et al., 2023). However, in the age group of 85 and above, the prevalence of Parkinson’s disease is higher in women than in men, aligning with the global trend of longer life expectancy in women. Research suggests that the prevalence of Parkinson’s disease is marginally elevated in Japanese women, a phenomenon potentially linked to the extended life expectancy in Japan, particularly among females, which consequently contributes to a slightly higher incidence of Parkinson’s disease in this demographic (Kimura et al., 2002).

Emerging research increasingly supports the notion that the utilization of calcium channel blockers is correlated with a substantial decrease in the risk profile for Parkinson’s disease (Tseng et al., 2021; Lin et al., 2024). However, the extent to which these agents may exert disease-modifying effects within the context of Parkinson’s disease is not yet fully understood and warrants further investigation. Furthermore, a plethora of studies has demonstrated that, upon controlling for alcohol and tobacco consumption, coffee and tea exhibit neuroprotective attributes that may significantly ameliorate the risk of developing Parkinson’s disease (Boulos et al., 2019; Mappin-Kasirer et al., 2020). This suggests a potential role for these beverages in the modulation of Parkinson’s disease risk, which is an area that merits further exploration within the scientific community. Additionally, a compelling corpus of evidence is accumulating, indicating an inverse relationship between tobacco use and the risk of Parkinson’s disease. It is important to note, however, that while tobacco use may present a protective effect against Parkinson’s disease, this benefit does not compensate for the broader detrimental impact that smoking has on an individual’s health (Xu et al., 2024).

Amidst the escalating global demographic shift towards an aging population, the incidence of Parkinson’s disease is anticipated to surge, with projections estimating a 1.5-fold increase by 2035. In China, where the aging crisis is particularly pronounced, the prevalence of Parkinson’s disease is expected to surpass half of the global patient count by 2030 (Xu et al., 2024). Consequently, Parkinson’s disease has emerged as the second most prevalent neurodegenerative disease worldwide and the third most significant health threat to middle-aged and older adults, following closely behind cancer and cardiovascular diseases (Ha et al., 2024).

Additionally, the study identifies major causes of death among Parkinson’s disease patients, including aspiration pneumonia, dementia, senile pneumonia, cancer, and cardiovascular diseases. Approximately 60% of patients suffer from cardiovascular dysfunction, and some die suddenly (Simon et al., 2020; Park et al., 2020; Scorza et al., 2022). This emphasizes the critical role of quality healthcare services. In low SDI countries, EAPC in ASDR is 0.30, exceeding the levels observed in middle and high SDI regions. These findings highlight Parkinson’s disease as a growing global health challenge, particularly affecting the elderly and men. With the ongoing global aging trend, the burden of Parkinson’s disease is expected to continue rising.

This study employs the BAPC model to forecast the trajectory of Parkinson’s disease globally from 2022 to 2035. BAPC is particularly useful for projecting future rates based on historical data, making it an invaluable tool for public health planning and analysis. Our analysis anticipates an increase in the incidence and prevalence of Parkinson’s disease, as well as the burden measured by DALYs, with the exception of a modest decline in the ASDR. Concurrently, we observe an upward trend in both the ASIR and ASPR of Parkinson’s disease. Given the intricate nature of Parkinson’s disease’s etiology, it is imperative that research priorities are allocated towards unraveling the underlying causes and advancing therapeutic interventions. It is also crucial that public health strategies are bolstered to facilitate early diagnosis and prevention initiatives for Parkinson’s disease. Emerging evidence suggests that the use of α-synuclein seeding assays offers a more precise method for differentiating individuals with Parkinson’s disease from healthy counterparts. Furthermore, this approach may hold prognostic value, potentially allowing for the anticipation of disease progression and staging (Ben-Shlomo et al., 2024). Furthermore, there is a need to refine the allocation of healthcare resources on a regional basis to address the increasing burden of Parkinson’s disease.

Therefore, it is crucial to enhance research, strengthen preventive measures, and develop targeted treatment strategies. Especially in resource-limited countries and regions, improving early detection, diagnosis, and treatment capabilities is essential to address this challenge effectively. Through these efforts, we can better manage the global burden of Parkinson’s disease and improve patient quality of life.

## Data Availability

Publicly available datasets were analyzed in this study. This data can be found at: https://vizhub.healthdata.org/gbd-results.

## Footnotes

^1^ https://www.healthdata.org/researchanalysis/gbd
