# Supplementary material for: Global, regional, national epidemiology and trends of Parkinson’s disease from 1990 to 2021: findings from the Global Burden of Disease Study 2021
Source: Front Aging Neurosci. 2025 Jan 10;16:1498756. doi: 10.3389/fnagi.2024.1498756 (PMC11757241; doi:10.3389/fnagi.2024.1498756)
Supplement: Supplementary file 1 [file Table_1.DOCX]

**Supplementary materials**

**Table S1. The incidence, prevalence, DALYs and their change trends of Parkinson’s disease from 1990 to 2021**

| Characteristics | Incidence cases | | | Prevalence cases | | | DALYs cases | | |
| --- | --- | --- | --- | --- | --- | --- | --- | --- | --- |
|  | 1990 | 2021 | 1990-2021 | 1990 | 2021 | 1990-2021 | 1990 | 2021 | 1990-2021 |
|  | Both (95%UI) | Both (95%UI) | Change (%) | Both (95%UI) | Both (95%UI) | Change (%) | Both (95%UI) | Both (95%UI) | Change (%) |
| **Global** | 417134.69  (370654.53-466859.89 | 1335142.12  (1196373.04-1485216.84) | 220.07  (210.02-229.15) | 3148394.56  (2749313.80-3629278.39) | 11767271.97  (10438278.37-13419874.68) | 273.76  (260.22-287.26) | 2854049.82  (2642177.71-3069764.10) | 7471821.42  (6736684.88-8137066.75) | 161.80  (146.07-177.39) |
| **Sex** |  |  |  |  |  |  |  |  |  |
| Male | 222778.35  (198558.78-249409.69) | 762142.24  (683406.98-850674.64) | 242.11  (231.32-252.06) | 1558967.50  (1360361.50-1801027.72) | 6438639.86  (5672343.52-7401532.78) | 313.01  (296.60-327.89) | 1528363.66  (1409605.37-1661598.63) | 4271994.64  (3843068.34-4706572.92) | 179.51  (157.02-200.85) |
| Female | 194356.34  (172763.05-217680.62) | 572999.88  (514626.31-637747.38) | 194.82  (184.58-204.36) | 1589427.07  (1396725.56-1826889.42) | 5328632.12  (4748783.45-6042536.89) | 235.25  (222.81-247.95) | 1325686.16  (1204600.43-1439419.28) | 3199826.78  (2841371.48-3536422.94) | 141.37  (123.73-160.53) |
| **SDI** |  |  |  |  |  |  |  |  |  |
| High | 130050.41  (117351.75-143567.08) | 327961.71  (304409.20-352285.61) | 152.18  (141.61-163.55) | 1043331.12  (930293.62-1177957.26) | 2822911.56  (2608235.77-3048228.44) | 170.57  (156.53-186.03) | 856555.36  (794336.82-910111.03) | 2012286.91  (1802337.39-2167256.61) | 134.93  (124.46-142.01) |
| High-middle | 120875.25  (107308.27-135760.59) | 367914.10  (322893.75-418790.63) | 204.38  (192.25-220.80) | 950115.59  (834882.27-1092577.34) | 3455524.57  (3004050.99-4008210.37 | 263.70  (246.75-283.45) | 794054.16  (732075.41-851403.68) | 1859319.14  (1656530.48-2072133.80) | 134.16  (116.06-152.97) |
| Middle | 96793.70（84662.37-110440.45） | 435098.08（379286.32-492734.48） | 349.51  (331.95-368.99) | 687048.58  (590858.07-814397.99) | 3949951.97  (3413736.28-4611871.68) | 474.92  (444.99-503.89) | 701919.01  (634976.63-763784.85) | 2217518.00  (1971931.77-2467378.99) | 215.92  (185.47-248.93) |
| Low-middle | 51953.32  (46223.71-58310.04) | 158405.68  (141540.72-176126.65) | 204.90  (195.85-215.41) | 353410.82  (302918.04-413110.40) | 1210606.95  (1049177.01-1415664.33) | 242.55  (230.16-256.20) | 364127.78  (316909.83-426573.52) | 1053494.62  (933747.03-1172786.74) | 189.32  (160.11-216.80) |
| Low | 16987.16  (15193.28-18976.11) | 44822.49  (40182.46-49572.55) | 163.86  (156.75-171.31) | 110963.92  (94273.53-130093.61) | 320799.57  (276425.45-372375.88) | 189.10  (179.20-198.99) | 133978.67  (115315.91-160066.26) | 322628.46  (274809.97-378298.55) | 140.81  (116.15-177.70) |
| **Regions** |  |  |  |  |  |  |  |  |  |
| Andean Latin America | 2183.62  (1983.39-2400.56) | 9787.66  (8728.51-10927.77) | 348.23  (316.49-388.17) | 17994.16  (15491.37-20866.15) | 92644.78  (78378.56-108908.65) | 414.86  (361.48-474.73) | 15939.15  (14262.56-17647.60) | 55083.14  (46987.55-64115.56) | 245.58  (199.64-299.63) |
| Australasia | 2319.71  (2058.15-2516.23) | 6709.53  (6131.61-7499.99) | 189.24  (166.72-222.76) | 15895.54  (13328.46-18305.89) | 50041.38  (42899.96-59826.76) | 214.81  (175.18273.46) | 18026.33  (16829.65-19116.47) | 46462.21  (41207.75-50050.73) | 157.75  (139.77-171.18) |
| Caribbean | 2062.30  (1908.24-2207.31) | 5338.44  (4975.38-5724.88) | 158.86  (150.10-169.03) | 14304.34  (12351.28-16235.24) | 40118.53  (35114.81-45310.79) | 180.46  (165.66-197.05) | 18143.02  (16939.98-19250.81) | 43021.71  (38599.87-47700.92) | 137.13  (116.26-159.36) |
| Central Asia | 4445.67  (3977.86-4920.86) | 8713.71  (7945.62-9479.62) | 96.00  (82.37-111.46) | 33155.04  (28027.21-38423.21) | 60308.57  (51414.91-69116.05) | 81.90  (63.04-105.56) | 28049.67  (25027.86-31839.91) | 46866.45  (42726.72-51381.84) | 67.08  (48.46-88.05) |
| Central Europe | 17216.56  (15758.02-18640.95) | 28542.03  (26431.61-30625.29) | 65.78  (59.28-72.63) | 127262.69  (112462.40-142899.69) | 227814.51  (203147.42-249502.45) | 79.01  (69.58-88.79) | 119821.82  (113173.04-125876.74) | 205701.67  (189999.27-221098.25) | 71.67  (63.28-79.92) |
| Central Latin America | 7063.85  (6442.28-7738.66) | 29014.82  (26476.74-31819.99) | 310.75  (296.30-325.81) | 53186.07  (46766.19-60991.24) | 241563.38  (212859.78-274750.56) | 354.19  (334.56-374.09) | 52941.40  (50161.01-55754.12) | 180811.91  (162820.88-200168.41) | 241.53  (216.93-268.25) |
| Central Sub-Saharan Africa | 1423.16  (1239.04-1615.87) | 3860.79  (3408.25-4413.23) | 171.28  (153.57-190.66) | 9590.73  (7876.37-11556.93) | 27789.84  (22919.57-33249.23) | 189.76  (165.48-217.70) | 11980.58  (10033.87-14242.36) | 29038.79  (22452.78-35480.04) | 142.38  (93.89-215.51) |
| East Asia | 94427.43  (80165.82-109879.36) | 523098.68  (445049.76-607919.25) | 453.97  (419.00-489.82) | 672346.28  (565540.35-807514.33) | 5212482.89  (4415844.92-6184114.04) | 675.27  (619.23-729.36) | 709320.71  (620295.64-790943.09) | 2234332.89  (1898277.90-2598616.17) | 215.00  (169.72-270.56) |
| Eastern Europe | 29598.70  (25435.26-34236.44) | 39438.48  (34171.89-44856.05) | 33.24  (28.15-39.13) | 249710.81  (210305.63-296977.62) | 332572.73  (279154.99-391577.67) | 33.18  (28.63-37.97) | 188245.49  (174682.33-203538.47) | 272732.29  (249596.87-296743.24) | 44.88  (35.57-55.36) |
| Eastern Sub-Saharan Africa | 4967.51  (4422.12-5528.68) | 12485.79  (11258.78-13758.10) | 151.35  (142.68-159.24) | 32284.25  (27237.24-37990.43) | 86052.51  (74397.32-98843.58) | 166.55  (156.16-177.40) | 40139.45  (33815.40-46331.52) | 88083.31  (68860.62-111837.21) | 119.44  (89.92-163.91) |
| High-income Asia Pacific | 15676.23  (13559.11-18118.68) | 40940.37  (36458.59-45744.84) | 161.16  (140.63-185.04) | 125906.98  (107091.35-148914.15) | 295706.63  (255167.21-341779.65) | 134.86  (119.48-152.53) | 110466.27  (101397.14-118555.04) | 336580.84  (289249.44-368739.08) | 204.69  (178.28-225.21) |
| High-income North America | 42383.84  (36182.27-48800.59) | 102845.30  (95282.59-111010.83) | 142.65  (122.15-170.06) | 333847.32  (282525.03-393498.11) | 843346.65  (786077.66-906806.86) | 152.61  (124.57-183.75) | 261020.67  (238830.68-280383.41) | 688598.58  (612954.01-739622.03) | 163.81  (154.35-171.23) |
| North Africa and Middle East | 14690.70  (13242.40-16210.82) | 54380.13  (48759.63-59977.06) | 270.17  (256.28-285.29) | 96660.03  (82758.06-113106.49） | 404383.98  (349761.26-463328.17) | 318.36  (300.85-341.69) | 122305.81  (108766.93-134926.21) | 322772.50  (288211.08-354299.21) | 163.91  (140.01-192.53) |
| Oceania | 252.64  (220.86-289.52) | 691.04  (609.55-770.06) | 173.53  (152.49-195.54) | 1723.37  (1415.73-2036.36) | 4944.74  (4151.05-5812.12) | 186.92  (160.81-218.00) | 2024.18  (1629.21-2473.92) | 4802.59  (3901.63-6100.53) | 137.26  (96.97-186.55) |
| South Asia | 49018.98  (42578.85-56331.58) | 162627.12  (141805.41-185043.13) | 231.76  (220.79-245.05) | 333200.41  (283776.13-394942.79) | 1265216.02  (1069411.27-1504597.43) | 279.72  (263.70-296.67) | 322779.23  (266379.66-403225.45) | 1054949.50  (892663.73-1214219.90) | 226.83  (181.08-271.06) |
| Southeast Asia | 20675.05  (18665.23-22838.43) | 67603.17  (61544.01-74556.25) | 226.98  (215.85-240.52) | 144543.82  (125453.18-169291.10) | 502949.97  (443328.25-572656.56) | 247.96  (231.20-270.12) | 153530.01  (136758.99-172958.47) | 474793.15  (421929.84-542495.60) | 209.25  (179.34-243.19) |
| Southern Latin America | 5937.17  (5464.22-6375.53) | 12894.64  (11756.73-14524.91) | 117.19  (102.07-136.74) | 41984.56  (37088.21-46444.86) | 98466.67  (85012.73-116369.39) | 134.53  (113.38-164.27) | 39059.69  (36812.67-41366.70) | 76949.14  (70719.65-82629.00) | 97.00  (88.55-105.58) |
| Southern Sub-Saharan Africa | 1958.11  (1729.64-2231.78) | 4871.75  (4250.19-5543.06) | 148.80  (139.92-158.92) | 13490.44  (11597.60-15833.02) | 34070.78  (29185.90-39592.97) | 152.56  (142.08-163.35) | 12470.17  (10848.67-14420.91) | 34008.11  (30969.01-36835.73) | 172.72  (139.29-206.44) |
| Tropical Latin America | 7661.46  (6555.53-8800.10) | 27301.26  (23728.02-30990.85) | 256.35  (242.05-272.72) | 57718.78  (49030.56-68466.50) | 228978.38  (195204.05-269203.75) | 296.71  (279.32-315.12) | 53765.27  (50343.32-57287.88) | 182535.23  (165240.15-196903.62) | 239.50  (225.85-250.11) |
| Western Europe | 85896.26  (79497.19-92555.42) | 175481.71  (162555.89-188776.41) | 104.30  (96.43-112.98) | 728665.44  (667678.59-800684.14) | 1599324.14  (1439667.89-1754089.11) | 119.49  (108.95-130.45) | 520636.31  (482218.65-555765.79) | 967935.13  (863141.30-1052274.66) | 85.91  (77.20-91.79) |
| Western Sub-Saharan Africa | 7275.74  (6514.09-8125.59) | 18515.69  (16701.62-20440.50) | 154.49  (144.78-164.81) | 44923.49  (38367.25-51654.82) | 118494.91  (103384.41-136012.27) | 163.77  (152.44-178.55) | 53384.61  (46991.72-60159.93) | 125762.29  (105968.80-143304.89) | 135.58  (100.72-170.67) |

**Table S2. The ASIR, ASPR, ASDR, and EAPC of Parkinson’s disease from 1990 to 2021**

| Characteristics | ASIR (per 100,000 population) | | | ASPR (per 100,000 population) | | | ASDR (per 100,000 population) | | |
| --- | --- | --- | --- | --- | --- | --- | --- | --- | --- |
|  | 1990 | 2021 | EAPC (95%CI) | 1990 | 2021 | EAPC (95%CI) | 1990 | 2021 | EAPC (95%CI) |
|  | Both (95%UI) | Both (95%UI) |  | Both (95%UI) | Both (95%UI) |  | Both (95%UI) | Both (95%UI) |  |
| **Global** | 11.24  (10.01-12.49) | 15.63  (14.03-17.39) | 1.09  (1.07-1.11) | 86.28  (75.74-98.88) | 138.63  (123.06-157.62) | 1.52  (1.49-1.54) | 81.48  (75.26-87.57) | 89.59  (80.69-97.50) | 0.32  (0.28-0.36) |
| **Sex** |  |  |  |  |  |  |  |  |  |
| Male | 14.13  (12.59-15.75) | 19.72  (17.69-21.88) | 1.11  (1.09-1.13) | 99.22  (86.70-113.81) | 168.24  (148.41-191.71) | 1.70  (1.67-1.73) | 105.99  (97.61-114.91) | 117.47  (106.15-128.88) | 0.37  (0.31-0.42) |
| Female | 9.29  (8.30-10.35) | 12.36  (11.11-13.75) | 0.93  (0.91-0.95) | 77.09  (67.90-88.09) | 114.47  (102.05-129.78) | 1.25  (1.23-1.27) | 65.37  (59.40-70.88) | 68.56  (60.90-75.76) | 0.14  (0.11-0.17) |
| **SDI** |  |  |  |  |  |  |  |  |  |
| High | 11.40 (10.35-12.56) | 14.99  (13.97-16.04) | 0.92  (0.88-0.96) | 91.50 (81.85-103.08) | 125.17  (115.35-135.55) | 1.05  (0.99-1.11) | 74.27  (68.79-79.07) | 85.43  (77.18-92.02) | 0.60  (0.51-0.67) |
| High-middle | 12.67  (11.27-14.06) | 18.49  (16.31-21.03) | 1.20  (1.18-1.23) | 102.66  (90.25-117.98) | 173.40 (151.36-200.71) | 1.61  (1.57-1.64) | 88.94  (82.06-94.95) | 94.16  (84.06-104.78) | 0.110  (0.05-0.17) |
| Middle | 10.88  (9.48-12.26) | 16.87  (14.67-19.11) | 1.47  (1.44-1.51) | 76.14  (65.02-89.48) | 154.79  (133.73-179.22) | 2.29  (2.23-2.34) | 85.83  (78.01-93.47) | 91.07  (80.79-100.96) | 0.14  (0.09-0.18) |
| Low-middle | 9.95  (8.79-11.10) | 11.95  (10.71-13.14) | 0.66  (0.62-0.71) | 65.78  (56.26-76.20) | 91.89  (79.39-106.58) | 1.17  (1.12-1.23) | 75.94  (65.70-88.95) | 86.15  (76.86-95.69) | 0.44  (0.39-0.49) |
| Low | 9.08  (8.18-10.07) | 10.21  (9.20-11.29) | 0.42  (0.37-0.47) | 57.34 (49.02-66.75) | 72.87  (63.08-83.78) | 0.83  (0.76-0.91) | 78.87  (66.71-94.40) | 83.09  (70.40-97.88) | 0.30  (0.18-0.42) |
| **Regions** |  |  |  |  |  |  |  |  |  |
| Andean Latin America | 11.21  (10.21-12.29) | 16.76  (14.97-18.67) | 1.30  (1.28-1.32) | 90.60  (77.80-105.75) | 159.23  (134.66-187.20) | 1.79  (1.75-1.84) | 88.15  (78.90-97.13) | 97.63  (83.37-113.15) | 0.38  (0.36-0.40) |
| Australasia | 9.59  (8.65-10.35) | 11.81  (10.76-13.18) | 0.73  (0.67-0.78) | 65.45  (55.02-75.23) | 86.62  (74.70-102.76) | 0.94  (0.87-1.01) | 74.92  (69.85-79.43) | 77.81  (69.30-83.77) | 0.34  (0.31-0.36) |
| Caribbean | 8.28  (7.72-8.84) | 9.88  (9.19-10.60) | 0.54  (0.50-0.57) | 56.24  (48.78-63.72) | 74.29  (64.99-83.87) | 0.84  (0.77-0.91) | 73.83  (69.10-78.20) | 79.74  (71.55-88.40) | 0.34  (0.32-0.35) |
| Central Asia | 10.24  (9.18-11.27) | 12.16  (11.20-13.02) | 0.61  (0.57-0.65) | 77.28  (65.28-89.87) | 86.14  (74.03-97.94) | 0.37  (0.28-0.46) | 67.86  (60.37-77.51) | 72.28  (66.15-78.68) | 0.50  (0.45-0.55) |
| Central Europe | 11.58  (10.68-12.46) | 12.15  (11.31-12.98) | 0.13  (0.11-0.16) | 86.81  (76.85-96.86) | 95.57  (85.76-104.99) | 0.26  (0.23-0.29) | 84.16  (79.48-88.46) | 84.57  (78.03-90.95) | 0.41  (0.39-0.43) |
| Central Latin America | 9.28  (8.50-10.15) | 11.95  (10.90-13.08) | 0.65  (0.57-0.73) | 67.71  (59.74-77.08) | 99.21  (87.37-112.71) | 0.96  (0.84-1.08) | 74.77  (70.80-78.36) | 76.72  (69.18-84.83) | 0.45  (0.44-0.46) |
| Central Sub-Saharan Africa | 7.95  (7.21-8.80) | 8.49  (7.62-9.42) | 0.25  (0.16-0.33) | 51.96  (42.37-62.02) | 60.96  (50.59-73.47) | 0.51  (0.39-0.64) | 77.01  (64.87-89.63) | 76.23  (57.93-94.84) | 0.17  (0.11-0.23) |
| East Asia | 12.74  (10.90-14.60) | 24.16  (20.61-28.00) | 2.16  (2.11-2.21) | 91.04  (75.57-108.21) | 243.46  (207.08-285.31) | 3.17  (3.04-3.31) | 104.70  (92.85-115.82) | 107.68  (91.45-124.81) | 0.66  (0.58-0.75) |
| Eastern Europe | 10.68  (9.23-12.24) | 10.93  (9.53-12.37) | 0.01  (-0.07-0.08) | 90.92  (76.68-107.83) | 92.06  (77.52-107.98) | -0.04  (-0.16-0.07) | 70.79  (65.55-76.44) | 75.26  (68.96-81.95) | 0.32  (0.27-0.37) |
| Eastern Sub-Saharan Africa | 7.94  (7.15-8.75) | 8.71  (7.85-9.57) | 0.29  (0.26-0.32) | 50.16  (42.56-58.54) | 59.28  (50.98-67.75) | 0.50  (0.45-0.55) | 70.45  (57.63-82.34) | 69.51  (54.12-89.95) | 0.51  (0.49-0.52) |
| High-income Asia Pacific | 7.92  (6.84-9.08) | 8.23  (7.39-9.10) | 0.48  (0.38-0.58) | 63.62  (54.32-74.86) | 60.66  (52.91-69.77) | 0.41  (0.22-0.60) | 57.80  (53.03-61.90) | 60.24  (52.91-65.61) | 0.51  (0.29-0.73) |
| High-income North America | 11.47  (9.86-13.13) | 14.98  (13.89-16.13) | 0.80  (0.74-0.86) | 90.25  (76.66-106.09) | 121.33  (113.40-130.16) | 0.90  (0.83-0.96) | 69.59  (63.77-74.73) | 97.44  (87.02-104.56) | 0.07  (-0.08-0.22) |
| North Africa and Middle East | 10.37  (9.47-11.45) | 13.54  (12.22-14.88) | 0.90  (0.86-0.93) | 67.83  (58.19-78.36) | 102.69  (88.70-117.78) | 1.39  (1.34-1.44) | 94.83  (85.21-105.73) | 88.60  (78.74-97.00) | 0.51  (0.47-0.55) |
| Oceania | 10.67  (9.57-12.05) | 11.13  (9.86-12.41) | 0.23  (0.18-0.27) | 75.63  (62.83-90.53) | 82.67  (68.83-96.32) | 0.38  (0.31-0.45) | 96.13  (79.40-116.92) | 87.03  (71.23-109.69) | 0.30  (0.28-0.33) |
| South Asia | 10.15  (8.79-11.60) | 11.79  (10.27-13.28) | 0.58  (0.52-0.64) | 66.03  (55.58-78.11) | 92.49  (78.24-109.01) | 1.25  (1.17-1.33) | 73.28  (59.55-92.21) | 83.24  (70.52-95.84) | 0.60  (0.58-0.63) |
| Southeast Asia | 9.45  (8.61-10.37) | 11.46  (10.49-12.52) | 0.66  (0.63-0.68) | 65.37  (56.63-75.20) | 85.83  (75.67-97.87) | 0.92  (0.88-0.97) | 76.62  (67.94-86.85) | 87.93  (78.01-100.26) | 0.61  (0.61-0.62) |
| Southern Latin America | 13.03  (12.08-13.93) | 14.31  (13.06-16.14) | 0.41  (0.37-0.46) | 94.01  (83.19-103.79) | 108.65  (94.03-128.38) | 0.61  (0.51-0.70) | 88.70  (83.44-93.90) | 83.84  (77.08-90.11) | 0.31  (0.28-0.34) |
| Southern Sub-Saharan Africa | 8.25  (7.25-9.32) | 9.82  (8.62-11.08) | 0.55  (0.51-0.58) | 56.05  (47.61-65.95) | 68.45  (57.98-79.80) | 0.58  (0.51-0.65) | 55.96  (48.24-64.96) | 73.56  (66.74-79.49) | 0.30  (0.29-0.32) |
| Tropical Latin America | 9.27  (7.92-10.66) | 10.83  (9.38-12.26) | 0.44  (0.40-0.49) | 68.17  (57.83-80.50) | 90.55  (77.15-106.24) | 0.82  (0.73-0.90) | 71.37  (66.04-75.89) | 74.52  (67.23-80.35) | 0.45  (0.43-0.46) |
| Western Europe | 14.10  (13.11-15.15) | 17.71  (16.41-19.08) | 0.65  (0.57-0.74) | 118.76  (108.72-130.55) | 7113.44  (6407.11-7867.10) | 0.67  (0.55-0.79) | 82.39  (76.24-88.07) | 87.56  (78.67-95.40) | 0.19  (0.17-0.22) |
| Western Sub-Saharan Africa | 9.91  (8.92-10.95) | 11.78  (10.69-12.99) | 0.59  (0.55-0.63) | 5494.22  (4872.02-6124.65) | 6075.81  (5385.72-6757.27) | 0.70  (0.63-0.77) | 79.46  (70.17-89.37) | 89.18  (76.00-100.52) | 0.39  (0.37-0.40) |

**Table 3. The incidence, prevalence, DALYs and their change trends of PD from 1990 to 2021**

| location_name | Incidence cases | | | Prevalence cases | | | DALYs cases | | |
| --- | --- | --- | --- | --- | --- | --- | --- | --- | --- |
|  | 1990 | 2021 | 1990-2021 | 1990 | 2021 | 1990-2021 | 1990 | 2021 | 1990-2021 |
|  | Both (95%UI) | Both (95%UI) | Change (%) | Both (95%UI) | Both (95%UI) | Change (%) | Both (95%UI) | Both (95%UI) | Change (%) |
| Afghanistan | 624.91(555.14-706.94) | 981.14(876.69-1087.52) | 57.01(42.83-75.19) | 3782.24(3156.96-4608.09) | 6688.77(5647.13-7794.31) | 76.85(53.25-105.95) | 7851.14(5130.86-10853.39) | 9676.00(6996.43-12552.59) | 23.24(-5.78-56.94) |
| Albania | 232.75(201.09-264.05) | 598.31(519.08-667.04) | 157.06(135.64-178.45) | 1665.81(1359.26-1994.95) | 4523.24(3591.64-5327.64) | 171.53(138.01-207.64) | 1611.48(1441.50-1792.37) | 4008.68(3360.70-4706.25) | 148.76(107.63-195.30) |
| Algeria | 1196.85(1033.06-1358.37) | 4111.42(3584.54-4671.47) | 243.52(206.08-287.66) | 7559.28(6204.33-9066.35) | 30340.83(25199.81-36300.97) | 301.37(249.25-365.96) | 6944.40(5600.79-8608.90) | 22748.02(18713.26-28106.65) | 227.57(171.83-290.62) |
| American Samoa | 1.64(1.40-1.88) | 3.85(3.32-4.36) | 134.71(98.95-167.38) | 12.45(10.17-14.95) | 30.98(26.33-36.13) | 148.92(113.07-191.22) | 16.24(14.05-18.18) | 35.25(29.88-42.41) | 117.01(81.49-157.17) |
| Andorra | 7.51(6.32-8.80) | 24.42(21.76-28.20) | 225.09(185.22-271.82) | 59.71(48.48-71.31) | 221.37(187.77-259.73) | 270.76(218.80-328.91) | 44.21(34.38-56.69) | 116.16(89.20-146.76) | 162.72(95.52-243.30) |
| Angola | 245.33(216.00-279.78) | 859.59(747.17-991.95) | 250.38(218.19-285.55) | 1636.65(1350.25-1982.47) | 6104.43(5010.91-7414.13) | 272.98(228.41-323.96) | 1946.16(1620.33-2390.25) | 6383.10(5139.83-7870.50) | 227.98(147.89-337.67) |
| Antigua and Barbuda | 5.48(5.05-6.03) | 12.49(11.26-14.19) | 127.79(107.06-155.85) | 35.75(30.66-41.70) | 90.40(75.86-110.94) | 152.85(112.92-201.04) | 49.40(46.10-52.83) | 96.68(87.77-103.58) | 95.69(79.67-111.73) |
| Argentina | 4255.69(3878.24-4591.84) | 7935.61(7221.11-9196.30) | 86.47(69.35-108.02) | 30522.86(26590.26-33834.98) | 60063.51(50925.14-72724.04) | 96.78(72.58-128.67) | 27529.53(25760.74-29258.70) | 48675.08(44638.61-52457.95) | 76.81(68.05-86.16) |
| Armenia | 224.89(204.97-249.24) | 434.82(385.14-487.60) | 93.35(74.03-115.09) | 1761.45(1524.42-2036.14) | 3408.41(2829.62-4069.64) | 93.50(66.57-129.09) | 1504.38(1361.89-1665.28) | 2840.34(2497.01-3143.27) | 88.80(65.43-112.84) |
| Australia | 1954.94(1696.54-2127.92) | 5801.37(5267.41-6599.79) | 196.75(170.35-234.64) | 13489.76(10925.24-15775.52) | 43867.35(36907.37-53447.55) | 225.19(177.64-296.31) | 15202.59(14169.76-16179.44) | 39335.46(34827.76-42509.83) | 158.74(140.64-172.94) |
| Austria | 1690.46(1495.63-1908.10) | 3280.66(2683.89-3890.92) | 94.07(73.13-122.04) | 13659.55(11620.24-16170.30) | 29568.90(22458.50-36088.41) | 116.47(84.55-155.29) | 10714.47(9973.43-11465.84) | 18125.83(16284.42-19883.98) | 69.17(59.11-79.87) |
| Azerbaijan | 447.83(395.03-506.27) | 1098.22(959.63-1228.77) | 145.23(117.67-174.44) | 3331.09(2779.26-4039.62) | 7339.40(5967.40-8716.76) | 120.33(84.57-160.79) | 3043.92(2512.17-3678.10) | 5199.23(4398.15-6056.70) | 70.81(33.18-114.83) |
| Bahamas | 13.82(12.50-15.25) | 41.00(37.30-44.88) | 196.74(169.62-221.21) | 97.32(83.22-114.76) | 309.86(265.26-362.32) | 218.39(175.45-264.92) | 107.67(99.42-115.87) | 332.40(282.50-392.83) | 208.73(160.97-264.61) |
| Bahrain | 16.23(14.22-18.39) | 108.95(93.81-126.15) | 571.13(499.10-657.89) | 103.50(86.24-123.15) | 756.40(639.67-903.06) | 630.82(539.61-736.19) | 131.77(117.43-145.86) | 497.23(424.70-585.79) | 277.36(216.71-364.10) |
| Bangladesh | 4105.69(3658.16-4599.24) | 13926.11(12193.56-15857.77) | 239.19(214.67-270.93) | 28073.01(23155.43-33449.34) | 111599.58(93017.06-134849.46) | 297.53(255.49-346.93) | 31864.45(26057.98-40251.37) | 93074.38(70278.63-128227.78) | 192.09(123.19-293.33) |
| Barbados | 28.22(25.46-30.91) | 57.54(51.73-63.84) | 103.91(85.83-126.15) | 187.45(157.98-221.16) | 417.95(354.58-499.50) | 122.96(93.44-162.05) | 237.93(222.15-252.15) | 455.59(380.83-530.71) | 91.48(59.86-123.65) |
| Belarus | 1497.94(1337.35-1657.44) | 2043.28(1854.50-2257.50) | 36.41(24.68-47.52) | 12559.85(10391.66-14969.10) | 17134.67(14487.15-20023.94) | 36.42(21.50-53.40) | 10062.58(8842.97-11406.87) | 13894.89(12018.52-15843.46) | 38.08(16.92-65.96) |
| Belgium | 2050.36(1854.60-2353.97) | 3923.62(3495.91-4399.98) | 91.36(70.38-118.09) | 16619.86(14565.33-19416.97) | 36419.77(31040.13-41905.31) | 119.13(92.93-153.01) | 13230.63(12210.09-14154.66) | 22456.39(19676.34-24773.59) | 69.73(60.42-80.36) |
| Belize | 8.03(7.33-8.75) | 28.75(26.21-31.59) | 258.21(223.32-300.92) | 55.12(46.84-64.96) | 215.61(183.21-253.49) | 291.19(230.88-371.46) | 56.78(52.75-61.07) | 220.25(195.54-244.98) | 287.89(247.67-330.44) |
| Benin | 184.86(165.03-205.24) | 492.01(432.54-550.95) | 166.15(140.43-191.75) | 1103.90(917.56-1315.09) | 3121.50(2599.37-3752.48) | 182.77(140.25-226.58) | 1346.31(1137.02-1575.72) | 3200.58(2611.01-3864.08) | 137.73(92.94-189.26) |
| Bermuda | 7.25(6.56-7.97) | 18.49(16.83-20.43) | 154.87(129.59-184.78) | 48.38(40.98-57.33) | 135.70(115.16-155.23) | 180.47(137.20-229.60) | 59.37(47.32-67.12) | 102.17(85.58-123.41) | 72.08(41.15-129.07) |
| Bhutan | 17.97(15.38-20.76) | 71.32(61.88-81.89) | 296.94(256.67-345.70) | 128.63(103.40-155.85) | 541.85(437.52-656.25) | 321.25(260.18-398.40) | 135.99(101.39-171.15) | 523.05(439.41-605.63) | 284.61(200.15-396.76) |
| Bolivia (Plurinational State of) | 384.38(354.70-413.50) | 1656.39(1445.02-1860.63) | 330.93(284.93-381.92) | 2948.27(2618.70-3306.89) | 14012.05(11576.24-16929.77) | 375.26(304.52-470.95) | 3091.10(2374.89-3745.07) | 9500.15(7457.53-12044.18) | 207.34(149.50-285.64) |
| Bosnia and Herzegovina | 498.12(435.79-555.87) | 837.44(733.83-935.41) | 68.12(49.95-84.56) | 3566.85(2910.37-4222.45) | 6338.76(5129.14-7780.77) | 77.71(49.49-107.33) | 3253.86(2884.66-3592.99) | 5856.90(4807.03-6954.92) | 80.00(48.57-114.73) |
| Botswana | 37.87(32.25-44.44) | 124.94(109.68-142.00) | 229.96(190.31-273.90) | 251.34(200.12-307.30) | 855.40(712.70-1018.87) | 240.34(193.74-297.36) | 284.97(227.31-347.10) | 720.70(608.73-878.72) | 152.91(101.72-228.31) |
| Brazil | 7478.85(6379.14-8627.44) | 26716.75(23173.90-30414.85) | 257.23(242.72-273.91) | 56284.65(47684.26-66803.74) | 223990.72(190653.58-263503.11) | 297.96(280.41-316.70) | 52648.85(49239.66-56039.06) | 178349.88(161427.26-192364.34) | 238.75(224.82-249.47) |
| Brunei Darussalam | 13.59(12.44-14.83) | 47.56(42.84-54.04) | 249.84(219.08-295.45) | 67.74(57.07-80.30) | 289.98(246.63-346.51) | 328.07(262.25-411.16) | 74.29(44.74-100.30) | 218.95(142.78-285.22) | 194.71(146.19-261.02) |
| Bulgaria | 2128.05(1940.12-2330.00) | 2107.21(1919.65-2343.91) | -0.98(-7.96-7.11) | 14932.02(13513.09-16784.88) | 15191.85(12666.15-17795.08) | 1.74(-10.50-15.60) | 11636.79(10782.02-12503.24) | 16590.32(14878.04-18284.17) | 42.57(27.82-58.79) |
| Burkina Faso | 365.82(319.61-410.31) | 866.60(781.40-964.26) | 136.89(114.39-165.24) | 2158.06(1748.82-2578.61) | 5388.84(4547.84-6393.58) | 149.71(111.84-197.37) | 2635.58(2072.50-3381.67) | 5839.22(4556.15-7476.50) | 121.55(83.79-168.73) |
| Burundi | 167.88(148.98-190.54) | 342.68(303.18-385.73) | 104.12(86.69-122.66) | 1050.48(859.43-1279.53) | 2332.27(1923.95-2757.16) | 122.02(92.56-153.38) | 1567.03(1178.44-1984.85) | 2630.12(1706.41-3416.93) | 67.84(25.29-141.25) |
| Cabo Verde | 20.84(18.15-23.77) | 47.91(42.89-53.94) | 129.85(107.30-154.77) | 144.49(117.29-174.94) | 343.76(283.93-401.59) | 137.90(100.76-176.13) | 162.91(135.60-194.33) | 349.13(277.45-410.41) | 114.31(58.36-171.24) |
| Cambodia | 319.42(284.55-354.77) | 1097.81(975.57-1227.37) | 243.69(215.20-278.43) | 2212.14(1836.64-2641.59) | 8092.51(6841.98-9486.20) | 265.82(221.25-315.00) | 2933.09(2485.35-3436.73) | 8674.01(6878.21-10527.49) | 195.73(136.01-269.58) |
| Cameroon | 397.43(352.69-438.94) | 1241.48(1097.15-1408.39) | 212.37(187.71-239.81) | 2374.24(1991.95-2793.86) | 7839.82(6506.59-9304.61) | 230.20(193.36-271.91) | 3285.59(2800.68-3841.88) | 9071.59(6971.59-11616.99) | 176.10(113.70-260.40) |
| Canada | 4948.31(4690.25-5260.38) | 15582.77(14784.25-16298.78) | 214.91(200.37-230.29) | 41147.08(38636.05-44247.56) | 151342.14(141798.25-160308.75) | 267.81(246.82-287.64) | 28378.26(26293.54-30496.80) | 75625.37(67306.41-82613.93) | 166.49(153.60-177.67) |
| Central African Republic | 75.67(66.05-85.55) | 149.72(130.91-170.14) | 97.86(82.59-119.16) | 488.28(392.59-592.16) | 1020.03(833.09-1227.04) | 108.90(87.15-137.01) | 663.68(509.58-797.12) | 1150.39(884.75-1440.07) | 73.34(40.88-112.67) |
| Chad | 234.82(205.96-263.77) | 481.82(424.65-546.54) | 105.19(88.67-124.11) | 1419.10(1164.64-1699.18) | 3064.34(2528.46-3650.29) | 115.94(93.47-143.37) | 1596.57(1302.29-1921.01) | 3570.84(2850.44-4429.03) | 123.66(78.61-176.57) |
| Chile | 1190.76(1098.85-1302.47) | 4103.62(3661.99-4529.95) | 244.62(221.28-270.91) | 7998.06(6926.95-9098.49) | 31416.70(26346.78-36621.98) | 292.80(255.16-339.81) | 8051.40(7545.77-8476.48) | 22089.77(20044.99-23772.63) | 174.36(159.98-187.21) |
| China | 91492.12(77329.44-106861.77) | 508377.67(430499.26-592747.86) | 455.65(419.88-493.05) | 651832.67(546481.86-786521.70) | 5077056.87(4277756.31-6049742.04) | 678.89(621.46-734.36) | 685008.67(597880.02-765406.72) | 2159514.01(1826196.33-2521343.81) | 215.25(168.29-272.87) |
| Colombia | 1420.23(1259.73-1568.37) | 6063.14(5307.87-6909.80) | 326.91(288.02-371.51) | 11179.00(9340.04-13084.19) | 53928.83(45027.43-62706.71) | 382.41(329.00-437.74) | 10032.97(9387.23-10678.48) | 38480.44(33354.19-44091.48) | 283.54(237.80-331.03) |
| Comoros | 13.64(12.03-15.23) | 38.77(33.69-43.80) | 184.18(159.17-213.04) | 88.14(72.93-105.53) | 263.54(212.57-320.21) | 198.98(157.36-243.34) | 111.79(89.08-137.90) | 303.22(224.77-398.67) | 171.25(103.27-273.87) |
| Congo | 81.82(70.67-91.76) | 222.17(195.57-254.67) | 171.54(149.50-196.47) | 536.90(439.76-642.58) | 1572.33(1320.60-1923.30) | 177.67(147.06-213.90) | 721.66(584.27-882.10) | 1564.45(1299.28-1881.36) | 116.78(74.25-187.23) |
| Cook Islands | 1.40(1.22-1.59) | 3.31(2.82-3.96) | 137.05(97.27-177.95) | 10.12(8.45-12.32) | 27.17(22.13-33.60) | 168.54(118.74-223.88) | 10.94(9.48-12.49) | 19.81(16.10-25.38) | 81.05(50.35-117.36) |
| Costa Rica | 171.78(153.18-188.76) | 671.64(589.55-771.81) | 291.00(258.39-343.11) | 1366.58(1147.50-1597.25) | 5920.25(5030.15-7216.20) | 333.22(282.57-409.41) | 1080.05(993.28-1168.89) | 3832.28(3418.98-4266.32) | 254.83(224.71-287.84) |
| Croatia | 682.70(600.32-774.32) | 1190.66(1051.14-1322.74) | 74.40(57.49-92.88) | 5154.66(4279.88-6129.91) | 9772.37(7871.01-11625.81) | 89.58(65.09-117.54) | 5136.06(4796.79-5481.09) | 8553.79(7726.67-9287.41) | 66.54(51.12-80.54) |
| Cuba | 781.00(713.27-848.98) | 1896.23(1753.61-2064.35) | 142.79(129.36-161.64) | 5390.31(4588.97-6249.12) | 14697.50(12788.68-16915.55) | 172.67(147.77-202.76) | 6498.58(6112.12-6844.14) | 15787.70(14052.77-17615.70) | 142.94(120.11-167.94) |
| Cyprus | 146.13(121.75-173.57) | 397.86(335.53-477.53) | 172.26(140.98-208.82) | 1005.77(808.84-1226.88) | 3320.85(2671.09-4081.89) | 230.18(190.37-290.07) | 1119.34(957.96-1278.14) | 1788.69(1557.22-2032.68) | 59.80(36.47-89.85) |
| Czechia | 1450.66(1307.38-1580.26) | 2659.03(2364.01-2958.51) | 83.30(63.41-99.25) | 11466.15(9498.41-13320.11) | 22325.15(19188.02-26116.34) | 94.70(62.60-123.92) | 11069.61(10437.23-11711.66) | 19376.73(17248.61-21368.50) | 75.04(57.86-91.27) |
| Côte d'Ivoire | 318.81(283.02-356.72) | 1056.82(939.06-1170.91) | 231.49(200.75-268.05) | 1950.80(1597.05-2318.64) | 6719.73(5621.05-7900.33) | 244.46(202.62-295.56) | 2344.06(1987.81-2718.84) | 7286.70(5875.47-8933.04) | 210.86(149.17-295.74) |
| Democratic People's Republic of Korea | 1581.62(1389.69-1799.79) | 5489.76(4733.02-6456.56) | 247.10(203.03-307.76) | 11587.94(9630.88-13881.16) | 50747.50(41339.94-59634.47) | 337.93(273.44-433.90) | 12593.02(10075.38-15373.56) | 32266.71(25321.88-38795.55) | 156.23(104.94-225.53) |
| Democratic Republic of the Congo | 954.43(818.98-1115.98) | 2478.79(2158.02-2846.20) | 159.71(138.12-182.71) | 6502.25(5213.30-8018.94) | 18054.51(14456.89-21811.93) | 192.86(158.38-228.76) | 8014.83(6513.99-9801.84) | 18852.76(13640.30-23641.26) | 135.22(77.14-214.80) |
| Denmark | 869.30(760.06-965.58) | 1979.42(1672.56-2338.20) | 127.70(102.03-153.40) | 7016.66(5823.99-8074.55) | 17647.32(13817.93-21596.28) | 151.51(118.37-189.07) | 5562.67(5167.47-5980.61) | 11423.74(10287.59-12545.67) | 105.36(92.49-118.03) |
| Djibouti | 7.97(6.92-9.07) | 45.69(40.32-51.47) | 473.31(420.10-529.32) | 54.24(43.62-64.78) | 313.81(261.86-369.11) | 478.57(411.96-559.23) | 52.36(38.98-68.65) | 298.33(219.43-429.79) | 469.72(348.76-651.83) |
| Dominica | 5.67(5.11-6.19) | 8.82(7.99-9.63) | 55.49(45.28-66.61) | 36.21(29.79-42.81) | 61.52(51.68-71.62) | 69.89(51.22-92.16) | 63.03(53.18-76.46) | 84.56(70.50-100.78) | 34.14(15.78-56.25) |
| Dominican Republic | 263.38(237.92-293.99) | 1003.17(915.83-1090.35) | 280.88(246.94-313.33) | 1876.75(1581.08-2243.65) | 7244.91(6172.53-8474.93) | 286.03(241.30-338.14) | 2220.08(1916.52-2609.29) | 7389.41(6054.77-9095.90) | 232.84(159.18-328.67) |
| Ecuador | 503.50(448.94-570.66) | 2691.89(2349.59-3033.33) | 434.64(379.06-513.15) | 4244.16(3559.75-5170.83) | 24993.08(20494.53-29531.69) | 488.88(398.42-609.15) | 3272.93(3029.83-3538.61) | 13830.81(11753.28-16378.02) | 322.58(259.46-396.09) |
| Egypt | 2689.23(2360.85-3036.29) | 9065.94(7969.22-10103.95) | 237.12(208.15-271.98) | 17976.96(15182.56-21529.44) | 66271.43(56180.05-77721.86) | 268.65(230.15-320.28) | 22161.30(19548.77-24225.57) | 43668.19(37804.56-51645.62) | 97.05(69.43-141.24) |
| El Salvador | 258.11(233.79-283.15) | 762.07(682.50-851.41) | 195.25(166.43-225.10) | 1947.10(1669.75-2275.23) | 6347.89(5349.00-7304.34) | 226.02(181.63-270.08) | 2036.57(1837.95-2301.62) | 5155.23(4366.18-5985.94) | 153.13(112.36-197.56) |
| Equatorial Guinea | 12.80(11.28-14.25) | 46.79(41.03-52.85) | 265.48(231.84-306.89) | 84.76(69.62-101.25) | 326.53(266.46-386.65) | 285.23(236.29-340.83) | 115.86(91.65-141.23) | 315.09(241.44-415.32) | 171.96(93.18-271.62) |
| Eritrea | 64.62(56.60-73.84) | 197.07(174.50-219.25) | 204.99(177.91-234.64) | 436.55(357.75-528.11) | 1338.51(1111.64-1567.91) | 206.61(172.16-245.20) | 511.51(417.34-629.21) | 1589.00(1252.92-1956.06) | 210.65(152.24-304.37) |
| Estonia | 243.22(214.25-282.15) | 316.76(280.21-358.90) | 30.23(19.02-43.36) | 2285.78(1922.32-2790.02) | 2989.11(2529.14-3493.09) | 30.77(15.24-49.34) | 1622.51(1473.82-1788.96) | 2415.07(2137.93-2684.69) | 48.85(31.80-65.39) |
| Eswatini | 20.74(18.23-23.46) | 46.53(40.63-52.84) | 124.35(104.80-151.58) | 139.64(115.79-167.41) | 314.11(256.67-374.88) | 124.95(99.86-157.60) | 176.33(134.63-221.06) | 397.02(281.88-536.67) | 125.15(77.86-189.47) |
| Ethiopia | 1357.93(1153.66-1588.33) | 3389.25(2912.88-3884.68) | 149.59(135.35-164.34) | 8760.99(7343.81-10445.18) | 23629.64(19753.22-27695.58) | 169.71(153.64-191.71) | 11791.61(9930.38-15452.98) | 22091.25(16377.32-32159.30) | 87.35(44.17-165.02) |
| Fiji | 35.12(30.90-39.58) | 81.66(68.89-95.22) | 132.51(107.60-157.30) | 239.13(197.11-282.09) | 589.19(468.41-713.93) | 146.39(102.12-181.46) | 260.22(228.79-299.88) | 565.01(464.46-678.98) | 117.13(72.96-164.38) |
| Finland | 913.49(823.96-1043.96) | 2320.35(1982.93-2833.79) | 154.01(131.39-177.33) | 7515.70(6526.04-8779.82) | 21580.32(17821.56-27159.89) | 187.14(150.93-219.84) | 5839.10(5403.94-6246.68) | 13843.59(12235.95-15227.30) | 137.08(122.02-151.62) |
| France | 12103.37(11070.50-13250.80) | 25629.09(21562.26-28679.31) | 111.75(87.49-136.00) | 97106.64(84988.70-108847.40) | 238852.44(182562.49-278798.34) | 145.97(108.84-182.67) | 73893.31(68364.53-79139.11) | 138582.38(122577.62-153329.70) | 87.54(75.24-99.45) |
| Gabon | 53.11(47.32-59.62) | 103.73(92.90-117.83) | 95.30(75.62-117.40) | 341.90(283.36-407.10) | 712.02(599.38-844.60) | 108.25(80.66-141.27) | 518.38(395.76-658.77) | 773.00(633.42-955.04) | 49.12(18.94-89.89) |
| Gambia | 28.37(24.96-31.91) | 101.99(89.77-115.39) | 259.56(226.24-297.29) | 173.17(144.00-201.85) | 636.51(523.01-762.01) | 267.55(218.06-323.26) | 187.79(144.44-239.64) | 695.98(519.19-855.64) | 270.61(184.67-377.24) |
| Georgia | 623.58(556.99-695.88) | 603.93(556.41-658.88) | -3.15(-11.28-6.60) | 4942.38(4135.63-5908.81) | 4584.24(3996.09-5341.22) | -7.25(-20.00-7.79) | 4331.91(3841.84-4917.30) | 4651.00(4178.22-5122.85) | 7.37(-7.50-22.78) |
| Germany | 15478.14(14674.77-16109.19) | 45334.19(43543.94-47180.89) | 192.89(182.24-207.33) | 124536.30(116489.74-132366.17) | 421892.88(404969.44-439866.87) | 238.77(221.78-256.73) | 101188.83(93952.82-107919.68) | 221855.05(195243.23-243018.94) | 119.25(107.28-130.51) |
| Ghana | 408.67(357.94-458.05) | 1379.20(1210.37-1568.04) | 237.49(211.87-285.17) | 2700.29(2263.92-3197.50) | 9187.04(7709.78-11026.99) | 240.22(202.47-304.63) | 2803.28(2348.86-3329.84) | 9276.59(7533.52-11138.32) | 230.92(160.69-310.97) |
| Greece | 2110.60(1846.22-2383.81) | 4064.00(3617.82-4640.20) | 92.55(70.69-121.71) | 17142.67(14308.68-20552.38) | 38018.53(31737.41-46111.32) | 121.78(88.74-159.19) | 12555.23(11584.01-13518.03) | 28464.03(25260.42-31259.17) | 126.71(112.30-140.31) |
| Greenland | 3.99(3.47-4.56) | 10.49(9.07-11.86) | 163.19(131.65-193.71) | 24.52(20.01-29.46) | 69.53(57.75-82.46) | 183.55(130.48-234.45) | 31.41(20.76-40.83) | 63.63(48.90-77.72) | 102.58(62.37-160.81) |
| Grenada | 6.44(5.90-7.18) | 11.20(10.03-12.55) | 73.84(56.21-90.89) | 40.75(34.32-48.79) | 81.55(69.34-98.00) | 100.13(69.27-136.19) | 55.43(50.76-59.91) | 102.68(92.00-113.15) | 85.24(62.85-104.84) |
| Guam | 8.30(7.03-9.79) | 24.99(22.48-29.71) | 200.89(142.62-277.84) | 59.83(48.79-74.23) | 210.63(179.26-262.07) | 252.02(177.01-364.53) | 50.17(41.48-58.24) | 102.84(85.99-120.18) | 105.01(82.11-130.55) |
| Guatemala | 272.42(242.93-308.20) | 1137.93(989.64-1284.17) | 317.72(272.28-368.05) | 1949.59(1636.43-2332.64) | 8927.43(7409.72-10761.35) | 357.91(291.77-434.73) | 2104.39(1999.59-2216.42) | 6508.55(5712.04-7359.46) | 209.28(175.24-244.62) |
| Guinea | 300.60(261.34-332.98) | 560.77(503.08-622.87) | 86.55(69.34-101.36) | 1733.30(1402.09-2066.37) | 3417.64(2853.38-4012.64) | 97.18(70.47-125.52) | 2212.88(1775.22-2708.23) | 4176.98(3311.18-5165.54) | 88.76(46.15-149.27) |
| Guinea-Bissau | 34.17(29.81-38.37) | 63.51(56.11-72.25) | 85.83(65.03-107.43) | 200.28(158.75-242.01) | 392.91(325.24-470.47) | 96.18(63.01-135.57) | 309.02(236.10-374.89) | 520.05(409.07-619.92) | 68.29(34.01-114.38) |
| Guyana | 30.97(28.40-33.81) | 58.11(53.45-63.73) | 87.59(74.28-101.52) | 200.73(168.99-234.00) | 417.92(355.17-490.13) | 108.21(84.66-139.92) | 260.24(238.14-283.48) | 481.11(398.76-580.19) | 84.87(48.75-123.40) |
| Haiti | 255.28(232.62-280.61) | 633.98(577.41-697.26) | 148.34(135.24-163.50) | 1610.74(1342.51-1899.94) | 4306.47(3628.99-5074.87) | 167.36(142.38-195.90) | 2924.95(2007.59-3685.87) | 5876.53(4334.71-7624.42) | 100.91(48.68-182.19) |
| Honduras | 211.60(191.26-236.29) | 1009.06(893.30-1127.41) | 376.86(335.02-418.67) | 1383.66(1148.70-1642.12) | 6663.57(5463.89-8103.30) | 381.59(318.45-462.46) | 1880.29(1625.66-2133.54) | 8394.01(7010.08-10031.94) | 346.42(267.81-443.61) |
| Hungary | 1502.56(1371.44-1639.78) | 2217.08(2050.16-2440.33) | 47.55(37.43-60.94) | 11465.36(9928.54-13028.65) | 18015.99(15734.35-20875.01) | 57.13(36.57-78.22) | 11412.92(10769.57-12062.01) | 16454.69(14757.41-18131.97) | 44.18(32.70-56.41) |
| Iceland | 46.65(40.99-52.18) | 120.56(103.44-139.72) | 158.43(134.49-199.98) | 384.23(326.13-452.73) | 1060.40(864.01-1276.86) | 175.98(140.15-244.05) | 308.36(280.21-332.41) | 694.68(605.70-774.23) | 125.28(106.69-145.39) |
| India | 38891.65(33481.18-45114.10) | 133179.32(114417.44-153108.79) | 242.44(228.34-257.26) | 264803.37(224107.85-316270.66) | 1041163.36(872603.57-1234479.05) | 293.18(274.24-312.91) | 246602.91(198821.60-314035.73) | 850984.11(720185.58-980745.62) | 245.08(194.52-302.75) |
| Indonesia | 7014.38(6055.18-8113.00) | 22616.97(19493.93-26072.67) | 222.44(211.21-236.92) | 49756.65(41949.61-59524.53) | 161898.15(135477.52-194341.08) | 225.38(212.49-241.40) | 52300.11(44130.86-61799.98) | 163545.56(133208.52-197153.07) | 212.71(160.69-271.49) |
| Iran (Islamic Republic of) | 2058.78(1749.65-2392.88) | 9187.58(7934.73-10504.44) | 346.26(317.89-377.82) | 14241.52(12096.71-17018.65) | 72245.34(61225.50-84527.42) | 407.29(376.66-447.38) | 13899.37(8787.71-15884.53) | 49112.41(30078.28-55765.58) | 253.34(219.49-303.28) |
| Iraq | 686.16(613.11-769.70) | 2614.10(2266.83-3000.71) | 280.97(242.81-318.93) | 4512.03(3772.59-5446.65) | 18886.56(15770.59-22552.64) | 318.58(274.39-369.38) | 6176.71(5193.34-7445.99) | 17919.75(14397.03-21121.74) | 190.12(125.17-259.14) |
| Ireland | 562.29(506.21-661.29) | 1451.95(1241.84-1641.59) | 158.22(134.45-185.71) | 4417.93(3859.73-5164.03) | 12816.20(10609.87-14672.63) | 190.10(164.09-230.68) | 3562.77(3310.79-3803.18) | 7089.58(6324.49-7787.37) | 98.99(85.90-110.16) |
| Israel | 931.34(811.18-1122.56) | 2801.55(2367.42-3352.94) | 200.81(172.80-234.16) | 8123.81(6887.06-9884.30) | 26275.15(21630.74-31769.30) | 223.43(189.05-267.26) | 4639.80(4228.74-5053.94) | 12160.61(10723.41-13671.68) | 162.09(145.05-177.52) |
| Italy | 16477.79(13897.34-19047.66) | 22054.48(18455.35-25407.65) | 33.84(23.05-47.91) | 151340.77(127857.41-177913.05) | 186889.37(153047.07-221031.45) | 23.49(13.18-35.26) | 85761.54(77699.35-93783.34) | 141125.09(122874.29-154402.09) | 64.56(54.24-72.34) |
| Jamaica | 151.44(137.90-163.82) | 303.63(279.28-338.49) | 100.49(86.72-118.57) | 1060.99(887.70-1247.78) | 2291.21(1948.92-2751.70) | 115.95(93.49-147.31) | 1189.10(1107.43-1258.11) | 2419.09(2000.30-2884.23) | 103.44(67.74-141.73) |
| Japan | 13570.55(11577.51-15843.75) | 29524.90(25370.06-34034.61) | 117.57(99.82-137.13) | 111275.68(93927.28-132041.03) | 203811.53(168708.52-242338.10) | 83.16(71.18-96.57) | 92305.93(84598.05-98557.74) | 266944.21(225828.42-290756.41) | 189.20(162.95-205.67) |
| Jordan | 107.38(97.09-118.29) | 682.90(619.43-746.89) | 535.98(491.74-586.82) | 658.06(554.77-767.64) | 4599.73(4000.91-5270.53) | 598.98(518.50-680.44) | 829.24(693.54-994.22) | 3591.54(2973.15-4301.82) | 333.11(248.06-441.53) |
| Kazakhstan | 1185.56(1031.38-1342.62) | 2052.19(1827.16-2279.78) | 73.10(55.80-95.04) | 8769.74(7179.36-10510.89) | 14847.22(12491.53-17145.29) | 69.30(47.93-102.73) | 7573.60(7029.52-8216.36) | 12973.96(11653.34-14312.15) | 71.31(52.14-92.27) |
| Kenya | 569.33(486.99-662.51) | 1786.78(1541.41-2052.80) | 213.84(201.12-228.07) | 3871.25(3260.57-4608.81) | 12314.16(10365.38-14447.08) | 218.09(205.13-231.88) | 3676.39(2962.17-4664.77) | 12160.91(9687.22-15010.39) | 230.78(181.26-296.18) |
| Kiribati | 3.99(3.51-4.52) | 7.85(7.05-8.87) | 96.63(79.67-115.77) | 24.06(19.65-29.28) | 49.67(41.38-59.49) | 106.44(78.90-137.64) | 28.43(24.19-32.41) | 51.18(42.89-62.00) | 80.02(50.85-122.36) |
| Kuwait | 48.63(42.95-55.36) | 279.79(242.05-322.94) | 475.29(414.35-547.71) | 356.85(297.96-420.54) | 2325.12(1938.48-2789.17) | 551.57(465.15-661.39) | 441.71(400.97-478.49) | 1364.39(1162.35-1589.54) | 208.89(164.58-254.87) |
| Kyrgyzstan | 252.55(225.18-280.37) | 379.04(335.63-428.25) | 50.08(35.53-62.82) | 1958.42(1638.02-2325.89) | 2845.84(2393.91-3395.93) | 45.31(24.69-65.24) | 1704.22(1494.79-1934.96) | 2089.73(1818.31-2397.05) | 22.62(2.17-44.50) |
| Lao People's Democratic Republic | 165.53(146.55-183.56) | 418.75(371.99-472.46) | 152.98(125.08-188.03) | 1097.77(897.16-1309.38) | 2977.35(2542.69-3592.03) | 171.22(124.72-224.66) | 1441.81(1134.65-1811.04) | 3153.48(2527.84-3845.79) | 118.72(65.72-183.45) |
| Latvia | 406.50(369.73-449.09) | 456.71(413.84-502.46) | 12.35(4.31-24.08) | 3435.92(2955.94-3951.92) | 4044.07(3510.50-4879.46) | 17.70(2.54-39.69) | 2924.52(2647.27-3206.73) | 3580.38(3193.46-3934.49) | 22.43(9.01-37.06) |
| Lebanon | 180.23(159.55-200.83) | 722.35(635.37-817.00) | 300.80(262.49-347.58) | 1249.52(1039.80-1470.69) | 5861.22(4862.69-6989.39) | 369.08(312.98-435.93) | 1554.91(1035.56-2005.16) | 4408.51(3787.12-5130.25) | 183.52(118.26-330.08) |
| Lesotho | 54.08(47.33-61.19) | 83.32(73.34-94.86) | 54.08(37.94-71.24) | 365.88(296.77-438.43) | 546.33(459.02-660.02) | 49.32(27.54-72.01) | 427.84(353.07-520.06) | 708.97(542.83-879.96) | 65.71(25.14-123.13) |
| Liberia | 99.35(87.54-112.82) | 175.07(154.57-193.20) | 76.21(62.37-94.99) | 607.22(499.43-758.97) | 1189.25(993.85-1388.06) | 95.85(69.79-130.10) | 759.29(649.87-895.74) | 1228.91(899.76-1604.50) | 61.85(22.71-111.32) |
| Libya | 153.24(137.30-168.70) | 544.63(483.53-606.47) | 255.40(224.06-291.07) | 1063.01(892.65-1229.82) | 4218.27(3560.77-4857.93) | 296.82(250.30-350.52) | 1272.66(978.23-1623.65) | 4587.17(3125.67-7206.25) | 260.44(136.26-467.74) |
| Lithuania | 436.43(392.27-508.45) | 627.44(555.79-684.82) | 43.77(27.95-56.48) | 3998.97(3380.97-4788.34) | 5702.89(4736.65-6574.75) | 42.61(20.99-64.82) | 2943.43(2679.67-3237.77) | 4954.69(4428.58-5457.55) | 68.33(51.28-88.97) |
| Luxembourg | 83.34(72.98-97.03) | 198.47(171.18-227.26) | 138.15(112.22-173.07) | 659.87(550.67-805.29) | 1746.42(1439.80-2073.31) | 164.66(123.26-220.47) | 554.61(517.65-594.03) | 1085.08(966.15-1205.21) | 95.65(80.59-111.44) |
| Madagascar | 294.55(258.81-338.16) | 666.36(574.41-756.69) | 126.23(104.57-149.28) | 1969.32(1593.21-2387.67) | 4825.70(3969.17-5701.93) | 145.04(112.27-177.39) | 2376.50(1980.11-2844.98) | 4283.46(3206.90-5694.27) | 80.24(42.40-121.44) |
| Malawi | 252.17(222.82-282.20) | 542.91(473.34-605.28) | 115.30(95.60-137.57) | 1629.18(1329.22-1925.67) | 3678.47(3040.24-4369.47) | 125.79(95.71-160.49) | 1849.04(1463.46-2197.09) | 4085.16(2877.63-5388.28) | 120.93(80.09-178.08) |
| Malaysia | 820.97(735.60-911.87) | 3136.99(2743.56-3642.02) | 282.11(245.87-352.57) | 5816.24(4789.64-6855.63) | 23980.50(19825.71-29784.10) | 312.30(262.06-415.33) | 5526.01(4938.50-6192.39) | 20840.53(18793.21-23235.07) | 277.14(227.99-325.73) |
| Maldives | 7.78(6.84-8.76) | 36.61(32.58-41.34) | 370.56(311.52-443.49) | 53.11(44.07-63.01) | 271.42(227.19-322.11) | 411.06(336.80-515.47) | 58.53(46.83-68.34) | 183.41(155.71-211.64) | 213.34(158.23-296.53) |
| Mali | 277.22(244.15-308.16) | 690.90(616.07-775.40) | 149.22(124.31-174.37) | 1763.61(1476.43-2061.71) | 4554.27(3867.99-5380.19) | 158.24(119.40-195.19) | 2436.95(2051.28-2842.48) | 5860.05(4779.68-6996.35) | 140.47(96.59-193.28) |
| Malta | 58.82(50.74-68.29) | 186.05(166.26-215.31) | 216.27(187.57-257.99) | 452.41(371.31-547.12) | 1608.91(1366.66-1910.88) | 255.63(209.57-319.06) | 385.29(354.03-413.99) | 992.34(878.24-1105.52) | 157.56(137.97-177.80) |
| Marshall Islands | 1.86(1.62-2.12) | 3.89(3.37-4.52) | 109.01(90.19-130.35) | 12.04(9.79-14.75) | 26.26(21.75-31.75) | 118.08(93.66-146.44) | 16.17(13.93-18.33) | 28.00(22.53-34.48) | 73.16(37.14-119.64) |
| Mauritania | 90.92(79.92-101.45) | 210.65(184.60-241.99) | 131.68(111.06-153.18) | 534.05(442.43-637.68) | 1368.55(1129.34-1661.24) | 156.26(121.67-195.84) | 654.97(518.60-797.81) | 1382.05(1023.70-1821.85) | 111.01(67.19-172.73) |
| Mauritius | 62.87(56.83-70.32) | 199.61(173.23-223.37) | 217.51(188.35-250.38) | 465.88(394.51-553.86) | 1581.17(1305.36-1872.66) | 239.40(192.58-285.47) | 470.93(443.07-498.29) | 1298.91(1195.51-1387.84) | 175.82(159.21-191.88) |
| Mexico | 3660.92(3155.78-4191.69) | 14927.91(12886.62-16953.41) | 307.76(288.27-329.66) | 27173.51(23232.97-32167.33) | 122846.84(104360.05-144147.10) | 352.08(328.00-375.79) | 28719.76(27281.70-30204.88) | 91212.87(81300.73-102312.21) | 217.60(189.82-247.00) |
| Micronesia (Federated States of) | 5.47(4.90-6.13) | 8.09(7.07-9.29) | 47.78(33.84-64.30) | 34.47(28.77-41.03) | 54.29(44.41-65.32) | 57.50(36.22-77.93) | 48.85(40.83-57.18) | 56.44(45.64-70.39) | 15.53(-6.34-47.07) |
| Monaco | 10.79(9.31-12.69) | 18.96(15.94-22.77) | 75.70(55.79-104.29) | 90.81(72.38-109.59) | 171.42(137.99-214.78) | 88.78(61.85-127.08) | 76.29(60.67-92.54) | 126.13(101.29-150.01) | 65.34(38.99-109.10) |
| Mongolia | 94.78(85.09-106.48) | 176.86(156.88-199.99) | 86.59(68.29-106.46) | 656.17(541.78-813.91) | 1276.90(1060.83-1524.39) | 94.60(62.71-124.21) | 635.22(555.44-730.61) | 1045.92(895.02-1199.00) | 64.65(42.45-88.79) |
| Montenegro | 75.17(66.17-85.37) | 134.09(117.05-148.81) | 78.39(61.12-98.09) | 580.94(477.10-682.22) | 1004.53(827.97-1159.79) | 72.92(49.31-101.37) | 493.89(426.58-570.86) | 1005.32(874.94-1172.99) | 103.55(69.76-136.52) |
| Morocco | 1191.21(1057.52-1341.44) | 4145.84(3648.72-4639.08) | 248.04(216.72-289.28) | 7863.93(6449.41-9457.91) | 30352.80(25481.84-36131.51) | 285.97(233.50-358.67) | 9612.22(8021.70-11313.03) | 30506.82(24076.00-36064.03) | 217.38(149.86-281.36) |
| Mozambique | 377.55(333.54-434.46) | 795.64(698.64-904.68) | 110.74(92.15-134.49) | 2396.10(1969.12-2980.82) | 5253.98(4318.72-6232.21) | 119.27(93.14-157.85) | 2925.80(2333.83-3752.51) | 6403.12(4645.90-8646.80) | 118.85(73.72-165.70) |
| Myanmar | 1658.04(1471.24-1839.40) | 4542.93(4051.12-5004.59) | 173.99(151.58-196.68) | 11252.91(9207.69-13253.03) | 33300.51(27789.19-38511.12) | 195.93(160.85-230.71) | 13792.30(11363.99-16546.66) | 33984.54(27776.89-42343.73) | 146.40(95.22-219.58) |
| Namibia | 44.78(38.87-51.70) | 116.28(102.24-131.13) | 159.67(129.14-187.72) | 296.13(244.44-360.88) | 799.58(662.71-943.61) | 170.01(126.28-208.01) | 338.05(282.92-401.76) | 900.70(737.56-1078.80) | 166.44(116.61-222.92) |
| Nauru | 0.54(0.47-0.63) | 0.70(0.62-0.78) | 30.07(18.61-42.74) | 3.29(2.70-4.19) | 4.64(3.88-5.56) | 41.36(21.70-62.63) | 5.07(3.81-6.53) | 5.75(4.29-7.35) | 13.28(-11.19-45.06) |
| Nepal | 603.87(525.27-687.65) | 2338.24(2043.79-2670.47) | 287.21(254.96-321.71) | 4280.71(3483.25-5087.87) | 17936.97(14846.41-21445.63) | 319.02(269.79-374.98) | 4738.83(3904.84-5750.90) | 16647.95(13630.32-20167.43) | 251.31(179.15-341.48) |
| Netherlands | 3957.79(3510.47-4335.62) | 7133.07(6371.00-7939.85) | 80.23(64.57-94.63) | 34183.77(30100.25-38161.70) | 64546.23(56587.65-73480.44) | 88.82(69.17-108.71) | 21686.22(19765.13-23459.86) | 37284.82(33328.54-40803.10) | 71.93(63.48-80.19) |
| New Zealand | 364.78(303.30-429.98) | 908.16(769.07-1049.63) | 148.96(132.07-165.97) | 2405.79(1959.71-2947.50) | 6174.03(5048.16-7462.86) | 156.63(136.17-179.24) | 2823.74(2628.10-2997.44) | 7126.75(6351.78-7655.37) | 152.39(137.01-165.39) |
| Nicaragua | 153.21(138.30-166.34) | 682.98(604.47-752.06) | 345.78(306.96-385.09) | 1100.08(944.08-1262.50) | 5158.85(4260.23-5988.61) | 368.95(310.06-420.86) | 806.64(719.44-913.46) | 2834.57(2388.46-3267.20) | 251.41(198.27-305.28) |
| Niger | 193.20(166.25-219.04) | 639.20(562.69-734.36) | 230.86(195.61-269.28) | 1215.11(971.34-1443.01) | 4079.09(3417.86-4889.58) | 235.70(192.44-293.61) | 1344.45(1066.94-1649.12) | 4454.40(3543.17-5614.77) | 231.32(163.90-323.53) |
| Nigeria | 3757.31(3153.77-4398.48) | 9031.27(7779.67-10330.13) | 140.37(131.41-150.74) | 23479.84(19649.71-28003.06) | 57838.32(48951.47-67596.88) | 146.33(137.27-156.77) | 27348.63(23130.13-31940.51) | 58624.25(47998.38-69159.90) | 114.36(71.69-165.62) |
| Niue | 0.30(0.27-0.34) | 0.28(0.25-0.32) | -6.60(-16.04-5.02) | 2.20(1.79-2.67) | 2.17(1.86-2.65) | -1.23(-16.20-19.68) | 2.72(2.34-3.07) | 2.17(1.85-2.47) | -20.29(-32.00--7.35) |
| North Macedonia | 225.41(199.08-251.94) | 491.47(433.10-556.42) | 118.04(97.62-146.17) | 1541.30(1269.34-1833.79) | 3326.44(2800.71-3872.75) | 115.82(86.66-157.84) | 1519.43(1371.77-1691.64) | 3029.52(2562.16-3524.43) | 99.39(70.19-132.08) |
| Northern Mariana Islands | 1.71(1.50-1.92) | 6.14(5.36-6.92) | 259.60(214.19-312.29) | 11.98(10.07-14.05) | 45.57(38.23-52.98) | 280.29(221.47-345.26) | 10.41(8.35-12.66) | 34.96(29.59-40.24) | 235.94(174.90-294.91) |
| Norway | 375.51(318.39-436.18) | 1259.32(1068.93-1449.80) | 235.37(213.05-260.81) | 1846.66(1530.17-2203.63) | 10192.00(8415.63-12133.91) | 451.91(410.15-505.92) | 4972.84(4600.37-5200.35) | 10017.70(8990.45-10813.11) | 101.45(91.52-111.37) |
| Oman | 73.73(64.20-82.69) | 306.76(261.39-348.35) | 316.04(276.27-366.41) | 448.26(362.78-537.28) | 2023.20(1644.20-2353.03) | 351.35(288.83-437.37) | 533.69(423.29-667.20) | 1343.62(1136.92-1572.85) | 151.76(92.37-238.81) |
| Pakistan | 5399.81(4610.92-6282.96) | 13112.12(11354.35-14930.16) | 142.83(129.12-156.13) | 35914.69(30009.70-42874.86) | 93974.25(78927.05-110912.44) | 161.66(145.04-175.29) | 39437.04(33573.70-45932.38) | 93720.01(78877.16-112145.10) | 137.64(98.18-187.07) |
| Palau | 1.34  (1.17-1.55) | 2.95(2.55-3.57) | 119.54(90.78-149.91) | 9.11(7.46-10.79) | 21.16(17.70-26.64) | 132.25(95.95-174.01) | 8.36(7.28-9.80) | 15.51(12.96-18.56) | 85.56(49.17-127.90) |
| Palestine | 90.47(78.67-101.13) | 303.37(265.50-339.80) | 235.33(201.34-270.19) | 561.03(454.99-666.44) | 2117.75(1774.94-2499.09) | 277.48(225.75-335.25) | 701.77(592.21-840.08) | 1541.22(1345.54-1718.58) | 119.62(78.59-167.80) |
| Panama | 133.17(121.08-145.78) | 558.16(494.59-635.27) | 319.14(276.23-362.09) | 1066.26(899.61-1228.91) | 4830.46(4041.99-5630.84) | 353.03(288.95-421.25) | 872.69(804.93-939.80) | 3302.55(2708.50-3887.79) | 278.43(218.45-335.48) |
| Papua New Guinea | 140.29(120.32-163.26) | 434.68(376.70-490.52) | 209.84(176.19-245.02) | 962.90(782.23-1157.13) | 3085.70(2572.81-3649.85) | 220.46(179.60-269.58) | 1170.74(882.26-1532.36) | 3102.60(2350.69-4230.15) | 165.01(97.68-250.49) |
| Paraguay | 182.61(162.96-206.44) | 584.51(515.83-644.18) | 220.10(190.61-246.51) | 1434.13(1197.51-1713.64) | 4987.66(4228.97-5726.03) | 247.78(201.44-289.70) | 1116.42(964.16-1281.02) | 4185.35(3406.85-5109.17) | 274.89(205.51-358.72) |
| Peru | 1295.74(1156.59-1450.23) | 5439.38(4770.79-6238.31) | 319.79(277.44-367.18) | 10801.73(9160.05-12673.27) | 53639.64(44926.18-64504.74) | 396.58(328.62-468.82) | 9575.12(8473.60-10774.00) | 31752.18(26083.71-38352.45) | 231.61(172.37-306.49) |
| Philippines | 2361.59(2028.05-2740.57) | 7224.63(6222.62-8271.09) | 205.92(193.02-220.87) | 16815.40(14077.52-20111.85) | 54078.03(45856.34-64121.99) | 221.60(207.64-234.52) | 15105.89(13286.61-17237.69) | 51461.86(44756.22-58633.71) | 240.67(191.86-292.69) |
| Poland | 5141.48(4333.45-6007.63) | 9424.22(8466.85-10483.20) | 83.30(66.99-102.46) | 38774.97(31856.24-46999.61) | 78524.91(70580.06-87997.98) | 102.51(79.55-128.63) | 34566.48(32604.39-36609.25) | 65503.54(59046.86-71715.60) | 89.50(76.32-102.59) |
| Portugal | 1442.50(1239.09-1627.87) | 3585.57(3163.49-4065.88) | 148.57(122.81-174.84) | 10952.79(9082.29-12800.98) | 30913.46(24763.54-36440.01) | 182.24(140.78-222.36) | 9399.16(8771.72-10054.74) | 21772.89(19368.04-23521.06) | 131.65(114.87-144.56) |
| Puerto Rico | 323.99(288.95-347.62) | 779.30(700.45-851.03) | 140.53(123.14-161.03) | 2413.37(2042.25-2760.05) | 6228.51(5176.47-7211.49) | 158.08(127.74-193.92) | 2790.91(2639.01-2940.09) | 5880.15(5029.77-6696.08) | 110.69(82.11-137.24) |
| Qatar | 12.67(11.02-14.58) | 145.68(125.31-170.12) | 1049.40(930.72-1190.70) | 78.71(64.87-95.11) | 956.22(783.22-1166.02) | 1114.88(961.92-1294.62) | 105.30(91.26-120.30) | 524.31(418.84-661.36) | 397.91(284.55-562.07) |
| Republic of Korea | 1922.78(1697.23-2188.61) | 10513.37(9235.14-11644.36) | 446.78(380.67-521.48) | 13337.93(10981.19-15926.69) | 84805.63(70488.41-98828.93) | 535.82(430.35-664.88) | 16811.81(15015.73-21312.96) | 64882.81(52763.75-75141.09) | 255.89(230.12-278.35) |
| Republic of Moldova | 462.18(402.03-530.16) | 534.67(472.16-612.74) | 15.68(3.63-29.90) | 3761.58(3055.45-4600.42) | 4769.13(3883.34-5669.54) | 26.79(9.65-48.19) | 2911.03(2684.27-3149.91) | 3416.37(3100.93-3770.92) | 285.94(170.84-361.69) |
| Romania | 2888.67(2593.25-3163.72) | 4572.79(4172.15-4975.94) | 58.30(45.53-72.25) | 20274.41(16840.11-23640.83) | 35512.97(30013.70-40391.85) | 75.16(53.97-98.46) | 21034.14(19815.18-22260.36) | 36355.51(33157.55-39645.85) | 17.36(8.26-27.96) |
| Russian Federation | 18454.78(15593.00-21616.91) | 26382.06(22485.53-30388.67) | 42.96(36.60-50.38) | 153256.90(126902.49-185396.56) | 220449.95(182646.29-263338.66) | 43.84(38.77-49.75) | 119590.31(112296.52-127710.89) | 184953.88(170023.15-200599.06) | 72.84(58.49-89.35) |
| Rwanda | 189.89(165.79-214.11) | 441.25(386.38-502.82) | 132.37(111.77-153.07) | 1212.33(1000.04-1470.04) | 3044.28(2488.08-3722.24) | 151.11(123.23-181.18) | 1932.35(1431.95-2646.94) | 3585.70(2487.25-4719.23) | 54.66(46.05-63.37) |
| Saint Kitts and Nevis | 3.96(3.55-4.42) | 6.80(6.08-7.67) | 71.89(53.68-93.84) | 23.39(19.27-27.76) | 50.55(43.03-60.81) | 116.09(83.88-154.18) | 54.79(51.81-57.64) | 75.66(64.75-85.07) | 85.56(40.59-167.41) |
| Saint Lucia | 8.52(7.75-9.54) | 27.01(24.35-29.61) | 217.17(188.52-250.02) | 55.34(46.41-65.36) | 190.35(156.91-224.54) | 243.99(194.70-295.42) | 83.07(78.85-87.52) | 223.11(189.45-256.80) | 38.08(17.93-55.64) |
| Saint Vincent and the Grenadines | 5.86(5.28-6.42) | 13.90(12.70-15.31) | 137.10(117.50-159.43) | 39.52(32.73-46.77) | 99.23(83.98-117.34) | 151.09(119.47-185.26) | 53.43(49.78-56.75) | 119.34(107.86-131.76) | 168.56(131.38-205.50) |
| Samoa | 8.84(7.74-9.94) | 16.30(14.41-18.21) | 84.43(67.04-108.15) | 61.96(50.58-74.11) | 122.31(102.93-143.69) | 97.39(72.27-132.97) | 71.05(58.41-88.03) | 115.66(96.22-146.02) | 123.37(103.94-148.93) |
| San Marino | 4.88(4.17-5.64) | 11.26(9.27-13.64) | 130.77(105.64-158.55) | 44.52(37.15-52.43) | 115.65(91.86-146.62) | 159.76(124.16-198.97) | 24.14(20.84-27.43) | 38.17(29.13-48.53) | 62.80(40.12-90.37) |
| Sao Tome and Principe | 5.95(5.21-6.83) | 11.87(10.49-13.38) | 99.41(80.20-118.71) | 37.71(30.55-45.20) | 79.24(65.90-93.71) | 110.13(80.04-145.09) | 43.46(38.06-48.13) | 79.42(67.44-91.47) | 58.15(27.87-97.35) |
| Saudi Arabia | 663.22(580.03-741.83) | 2551.86(2229.46-2978.74) | 284.77(240.25-330.18) | 3866.73(3127.37-4643.66) | 17621.16(14918.72-21444.71) | 355.71(286.85-427.66) | 6286.70(4883.15-7822.26) | 16235.80(13681.59-19440.49) | 82.76(55.23-113.91) |
| Senegal | 290.50(255.81-322.28) | 824.02(739.17-930.33) | 183.66(153.43-215.46) | 1708.13(1409.66-2062.40) | 5141.44(4330.67-6158.58) | 201.00(152.40-251.47) | 2011.24(1664.12-2358.68) | 5724.64(4557.79-6991.32) | 158.26(103.62-247.81) |
| Serbia | 1187.69(1061.14-1332.31) | 2274.57(1965.08-2536.98) | 91.51(68.30-116.49) | 8505.80(7140.72-9971.23) | 16654.60(13026.26-19432.00) | 95.80(57.95-129.83) | 9188.84(8186.07-10099.31) | 14987.60(13195.45-16706.41) | 184.63(128.00-250.06) |
| Seychelles | 7.21(6.52-8.10) | 16.18(14.54-18.19) | 124.38(107.23-146.52) | 47.57(40.29-56.88) | 111.98(94.89-132.78) | 135.41(105.89-170.60) | 68.88(59.53-76.55) | 110.61(92.09-128.57) | 63.11(43.77-85.95) |
| Sierra Leone | 174.08(152.89-194.88) | 331.54(293.01-366.47) | 90.45(74.04-107.64) | 1040.87(850.51-1240.94) | 2120.17(1753.84-2468.72) | 103.69(74.55-137.37) | 1291.35(1087.84-1530.12) | 2316.06(1828.84-2881.18) | 60.58(38.80-82.19) |
| Singapore | 169.31(153.23-188.58) | 854.54(749.04-969.68) | 404.71(349.88-463.36) | 1225.63(1051.48-1435.66) | 6799.50(5727.90-8113.61) | 454.77(379.34-539.09) | 1274.24(1200.09-1346.16) | 4534.86(4075.89-4945.82) | 79.35(43.76-128.40) |
| Slovakia | 635.23(558.12-703.31) | 1058.87(944.44-1195.91) | 66.69(53.46-81.97) | 4903.48(4063.59-5808.70) | 8459.14(7079.79-10054.10) | 72.51(49.56-99.63) | 4976.31(4515.15-5516.07) | 6937.06(6057.40-7797.61) | 39.40(20.28-59.01) |
| Slovenia | 292.75(264.42-320.95) | 560.85(486.93-617.54) | 91.58(74.54-110.81) | 2395.76(2067.13-2763.79) | 4848.64(3988.39-5585.42) | 102.38(74.10-130.47) | 2005.83(1868.99-2148.80) | 4047.95(3522.26-4513.84) | 101.81(80.78-122.65) |
| Solomon Islands | 13.32(11.59-15.23) | 36.57(32.10-41.40) | 174.52(146.86-203.60) | 84.67(68.58-102.63) | 245.91(203.01-296.70) | 190.42(150.71-235.11) | 100.05(71.94-122.70) | 245.03(196.65-310.90) | 144.89(97.59-208.16) |
| Somalia | 137.47(121.53-155.11) | 368.66(320.93-424.91) | 168.18(144.66-194.16) | 916.34(741.43-1106.61) | 2491.50(2008.43-3037.59) | 171.90(142.81-202.43) | 1085.09(824.16-1413.79) | 2743.15(1929.75-3680.44) | 152.80(105.07-220.27) |
| South Africa | 1453.79(1248.06-1675.89) | 3913.51(3339.47-4519.17) | 169.19(157.15-184.01) | 10281.32(8642.02-12178.53) | 27809.94(23435.11-32881.47) | 170.49(159.30-185.34) | 8764.90(7377.69-10555.78) | 26644.57(23881.87-29055.50) | 203.99(161.99-244.65) |
| South Sudan | 178.75(153.68-205.54) | 245.37(210.32-275.48) | 37.27(24.85-51.17) | 1153.93(933.91-1410.99) | 1726.76(1390.92-2043.96) | 49.64(31.30-71.28) | 1613.20(1288.59-2017.90) | 2129.97(1624.79-2780.30) | 32.03(-1.37-73.65) |
| Spain | 9878.98(9106.96-11052.85) | 20226.29(17793.27-22773.04) | 104.74(88.28-122.66) | 82374.11(74789.04-91495.46) | 196221.23(163074.30-230613.22) | 138.21(113.95-164.15) | 49925.36(45625.60-53781.99) | 105166.94(92823.57-116264.30) | 110.65(97.39-121.69) |
| Sri Lanka | 912.89(796.25-1022.63) | 3043.34(2583.83-3542.50) | 233.37(202.50-268.88) | 6723.10(5520.76-7919.54) | 24397.50(19592.64-29953.18) | 262.89(215.73-312.68) | 6927.52(6243.92-7730.42) | 18165.31(13680.30-22902.47) | 162.22(97.50-235.61) |
| Sudan | 792.41(704.21-890.47) | 2062.43(1800.38-2341.48) | 160.27(134.10-192.15) | 5046.32(4165.34-6002.45) | 15128.04(12499.85-18215.99) | 199.78(156.31-255.28) | 6123.38(5035.52-7811.84) | 12500.55(10084.45-16143.75) | 104.14(64.73-165.49) |
| Suriname | 17.15(15.66-18.79) | 53.86(48.46-59.69) | 214.11(190.85-242.20) | 121.26(102.14-142.56) | 404.04(330.38-479.57) | 233.21(191.53-284.61) | 146.22(131.55-161.29) | 400.11(316.10-482.29) | 173.64(118.25-234.43) |
| Sweden | 1495.62(1252.77-1773.83) | 3430.49(2846.40-4016.98) | 129.37(113.54-148.95) | 12310.25(10124.81-14897.98) | 29646.46(24214.76-35463.49) | 140.83(123.01-162.02) | 12789.83(11794.06-13565.76) | 19844.11(17525.06-22011.64) | 55.16(43.57-66.56) |
| Switzerland | 1516.09(1307.91-1751.74) | 3202.33(2778.47-3729.60) | 111.22(90.69-134.32) | 12685.51(10485.03-14949.60) | 29802.31(24363.42-35896.64) | 134.93(106.98-167.61) | 9232.11(8380.30-9963.30) | 16734.25(14524.63-18556.40) | 81.26(69.44-92.20) |
| Syrian Arab Republic | 424.77(382.35-478.44) | 1454.54(1264.67-1675.46) | 242.43(206.46-278.51) | 2749.14(2316.64-3292.16) | 10607.30(8903.69-12749.28) | 285.84(238.12-342.22) | 3736.46(3120.75-4483.38) | 9614.07(7783.76-11791.32) | 157.30(100.08-244.54) |
| Taiwan (Province of China) | 1353.69(1277.18-1428.13) | 9231.25(8867.83-9648.27) | 581.93(547.39-627.15) | 8925.67(7986.22-9771.92) | 84678.53(80870.21-88790.74) | 848.71(773.96-940.64) | 11719.02(11112.20-12257.68) | 42552.16(37978.42-46691.76) | 263.10(234.43-286.82) |
| Tajikistan | 304.72(264.47-344.02) | 746.42(656.93-835.29) | 144.96(116.80-176.07) | 2045.03(1633.11-2480.61) | 4413.78(3595.43-5287.95) | 115.83(81.55-158.28) | 2891.19(2151.86-4283.42) | 4202.16(3572.05-4935.57) | 45.34(-0.73-95.72) |
| Thailand | 3409.85(3047.81-3828.55) | 12672.12(11329.31-14127.65) | 271.63(238.86-308.78) | 23078.95(19677.75-27698.06) | 98492.07(84271.72-114344.47) | 326.76(278.50-394.53) | 26515.46(22774.96-30418.01) | 92512.34(75639.59-113827.45) | 248.90(181.81-333.64) |
| Timor-Leste | 18.61(16.51-20.82) | 84.63(73.59-95.25) | 354.74(309.62-404.41) | 128.09(106.73-153.54) | 591.85(483.83-713.22) | 362.07(302.00-441.99) | 121.97(98.74-146.85) | 550.15(437.24-673.94) | 351.05(255.08-469.83) |
| Togo | 92.58(82.10-103.18) | 308.84(261.02-355.51) | 233.57(195.55-268.25) | 577.83(482.38-683.70) | 2011.11(1614.01-2424.95) | 248.04(198.76-292.29) | 608.54(482.81-745.66) | 2103.37(1577.43-2746.57) | 245.64(171.97-331.69) |
| Tokelau | 0.15(0.13-0.17) | 0.18(0.16-0.20) | 18.49(6.87-30.86) | 1.07(0.87-1.30) | 1.41(1.19-1.68) | 32.26(14.73-54.89) | 1.49(1.22-1.85) | 1.42(1.14-1.79) | -4.52(-19.28-13.56) |
| Tonga | 5.02(4.33-5.65) | 8.45(7.43-9.77) | 68.49(49.82-91.99) | 37.06(30.56-43.84) | 66.87(56.61-79.34) | 80.45(56.78-111.18) | 36.74(29.50-49.85) | 60.81(47.13-82.39) | 65.48(36.77-98.10) |
| Trinidad and Tobago | 66.21(59.78-72.90) | 172.77(156.47-196.92) | 160.94(136.30-184.94) | 462.85(395.13-541.47) | 1321.88(1095.34-1574.18) | 185.60(149.10-222.11) | 589.34(558.31-619.41) | 1356.74(1119.86-1615.63) | 130.21(90.78-175.26) |
| Tunisia | 431.84(366.34-504.17) | 1546.76(1317.22-1741.90) | 258.18(221.25-293.93) | 2895.44(2343.23-3544.05) | 11977.17(9788.78-14143.91) | 313.66(258.04-363.78) | 2762.32(2274.54-3433.80) | 8781.91(6736.40-11183.38) | 217.92(147.51-305.97) |
| Turkmenistan | 138.32(121.13-158.16) | 303.08(270.92-335.77) | 304.51(263.34-352.72) | 1076.98(891.34-1323.24) | 2299.07(1937.07-2675.23) | 113.48(85.20-148.49) | 912.63(847.32-989.66) | 2061.10(1699.52-2477.91) | 125.84(88.88-168.91) |
| Tuvalu | 0.71(0.63-0.82) | 1.23(1.10-1.37) | 119.12(99.29-142.55) | 4.67(3.93-5.63) | 8.77(7.51-10.25) | 87.69(64.60-112.81) | 6.26(5.26-7.27) | 9.39(7.65-11.53) | 49.90(29.39-77.90) |
| Turkey | 2862.59(2555.87-3207.22) | 11579.36(9985.99-13332.18) | 72.38(56.89-93.71) | 19170.60(16091.68-23004.36) | 87403.68(71251.41-106475.41) | 355.93(299.82-437.44) | 28014.25(24292.42-31641.01) | 73257.35(61506.95-85164.66) | 161.50(117.12-211.37) |
| Uganda | 457.70(403.00-516.68) | 1139.14(1006.14-1264.29) | 148.88(124.63-175.84) | 2962.02(2430.88-3529.76) | 7769.43(6322.51-9132.56) | 162.30(126.59-203.07) | 3559.50(2565.95-4855.43) | 8327.66(6012.95-10561.58) | 133.96(78.44-214.28) |
| Ukraine | 8097.64(6867.75-9507.25) | 9077.55(7700.96-10431.63) | 12.10(5.40-20.02) | 70411.81(57733.32-85339.58) | 77482.91(63945.14-92427.09) | 10.04(2.84-18.41) | 48191.11(42676.98-54282.04) | 59517.00(48433.76-71648.71) | 23.50(-0.16-53.73) |
| United Arab Emirates | 58.46(52.14-65.06) | 664.60(563.32-778.85) | 1036.91(906.13-1213.23) | 341.19(286.97-399.86) | 4581.63(3737.17-5523.02) | 1242.83(1059.68-1511.74) | 294.87(216.33-374.63) | 1600.96(1304.13-1936.84) | 442.95(329.54-650.76) |
| United Kingdom | 13613.91(11692.89-15729.22) | 22693.25(19560.22-25807.80) | 66.69(58.45-75.05) | 123536.25(105186.55-146531.19) | 198388.38(168849.25-230358.77) | 60.59(52.82-68.79) | 92742.30(86411.42-99000.01) | 136294.67(123939.61-147586.39) | 46.96(41.16-50.63) |
| United Republic of Tanzania | 689.93(612.67-765.17) | 1946.34(1811.96-2075.30) | 182.11(158.49-207.19) | 4468.77(3720.59-5171.96) | 13405.86(11850.31-15077.80) | 199.99(167.12-240.23) | 5606.99(4516.90-6775.63) | 13836.31(10527.32-18534.09) | 146.77(98.85-207.62) |
| United States of America | 37430.57(31240.99-43769.26) | 87250.42(79711.85-95477.37) | 133.10(111.61-162.22) | 292668.07(240636.19-351011.45) | 691921.76(635759.95-752237.75) | 136.42(107.69-170.18) | 232605.01(212342.18-250159.61) | 612898.79(546641.65-657597.28) | 163.49(153.60-171.20) |
| United States Virgin Islands | 9.80(8.76-10.84) | 30.76(27.54-33.83) | 213.85(182.52-254.31) | 63.75(51.83-74.54) | 195.72(166.42-234.82) | 206.98(160.25-268.35) | 88.33(71.45-108.13) | 162.52(133.16-197.85) | 84.00(42.91-134.25) |
| Uruguay | 490.43(445.82-537.75) | 854.69(775.99-952.39) | 74.27(61.52-89.72) | 3461.65(2985.56-3885.37) | 6981.03(6011.14-8284.64) | 101.67(81.35-131.07) | 3476.90(3273.44-3661.74) | 6180.05(5656.74-6644.70) | 77.75(68.31-86.40) |
| Uzbekistan | 1173.44(1028.30-1334.99) | 2919.16(2611.84-3204.52) | 148.77(121.23-182.57) | 8613.78(6983.57-10352.92) | 19293.71(15941.30-22194.11) | 123.99(85.09-169.42) | 5452.58(4410.85-6858.20) | 11803.02(10342.54-13367.45) | 116.47(69.29-173.85) |
| Vanuatu | 6.51(5.72-7.45) | 18.90(16.72-21.54) | 190.22(166.57-218.83) | 42.41(34.94-51.13) | 130.03(108.86-156.03) | 206.59(170.26-248.00) | 51.12(41.12-62.64) | 135.15(110.80-160.19) | 164.36(117.78-224.43) |
| Venezuela (Bolivarian Republic of) | 782.41(709.00-873.51) | 3201.92(2886.67-3533.82) | 309.24(274.70-348.89) | 6020.29(5083.11-6958.19) | 26939.25(23299.31-31243.66) | 347.47(301.45-401.55) | 5408.05(5053.57-5781.00) | 21091.41(17300.06-25661.55) | 290.00(221.19-365.22) |
| Viet Nam | 3886.00(3416.11-4353.92) | 12418.32(11081.80-14111.49) | 219.57(194.23-248.37) | 26886.91(21982.29-32434.48) | 92475.41(78357.69-111538.81) | 243.94(201.06-296.61) | 28045.39(23631.66-33706.37) | 79650.20(67270.85-93423.54) | 184.00(131.71-244.95) |
| Yemen | 319.63(284.46-358.19) | 1269.40(1125.81-1423.45) | 297.14(254.89-335.95) | 2082.83(1736.06-2483.65) | 9044.22(7597.64-10892.74) | 334.23(271.67-391.39) | 2804.74(2176.19-3754.81) | 8991.62(6850.24-11970.36) | 220.59(142.48-329.76) |
| Zambia | 204.59(181.49-229.09) | 529.02(465.80-587.86) | 158.57  (135.93-179.91) | 1291.55(1056.03-1540.37) | 3589.68(2976.07-4235.59) | 177.94(142.82-213.65) | 1451.60(1189.53-1752.65) | 3539.26(2760.76-4543.20) | 143.82(96.11-195.63) |
| Zimbabwe | 346.85(302.46-393.42) | 587.17(505.33-664.10) | 69.29(55.45-85.18) | 2156.13(1775.60-2595.65) | 3745.42(3083.98-4423.89) | 73.71(53.17-95.72) | 2478.08(2048.32-2862.09) | 4636.16(3758.61-5696.71) | 87.09(50.47-134.83) |

**Table 4. The ASIR, ASPR, ASDR, and their variations of PD from 1990 to 2021**

| Location name | ASIR (per 100,000 population) | | | ASPR (per 100,000 population) | | | ASDR (per 100,000 population) | | |
| --- | --- | --- | --- | --- | --- | --- | --- | --- | --- |
|  | 1990 | 2021 | EAPC (95%CI) | 1990 | 2021 | EAPC (95%CI) | 1990 | 2021 | EAPC (95%CI) |
|  | Both (95%UI) | Both (95%UI) |  | Both (95%UI) | Both (95%UI) |  | Both (95%UI) | Both (95%UI) |  |
| Afghanistan | 10.41(9.44-11.47) | 11.56(10.33-12.69) | 0.37(0.31-0.44) | 61.46(51.73-74.49) | 79.83(65.93-93.25) | 0.91(0.78-1.05) | 133.60(92.56-184.28) | 126.51(94.53-159.77) | -0.18(-0.21--0.15) |
| Albania | 12.24(10.89-13.62) | 13.03(11.43-14.44) | 0.26(0.23-0.28) | 90.43(73.89-108.19) | 98.96(79.10-116.15) | 0.34(0.29-0.39) | 96.47(86.00-107.63) | 90.23(75.70-106.10) | -0.04(-0.17-0.09) |
| Algeria | 11.38(10.28-12.56) | 12.97(11.56-14.57) | 0.43(0.38-0.49) | 72.07(59.62-85.43) | 95.76(78.73-114.35) | 0.93(0.86-1.00) | 80.03(67.24-93.70) | 80.08(66.95-95.92) | 0.23(0.14-0.32) |
| American Samoa | 8.65(7.37-9.88) | 8.90(7.60-10.16) | 0.20(0.14-0.26) | 69.39(57.22-82.79) | 73.81(62.36-86.46) | 0.33(0.24-0.42) | 98.05(84.80-109.58) | 88.14(74.85-106.08) | -0.23(-0.30--0.16) |
| Andorra | 13.24(11.38-15.52) | 15.85(14.07-18.38) | 0.59(0.55-0.63) | 108.47(88.09-129.62) | 140.34(118.02-164.99) | 0.85(0.79-0.92) | 82.03(64.05-104.96) | 72.67(55.78-92.06) | -0.13(-0.31-0.06) |
| Angola | 8.02(7.23-8.93) | 9.00(7.99-10.08) | 0.43(0.38-0.47) | 51.54(42.73-62.42) | 63.33(51.28-77.38) | 0.69(0.62-0.77) | 73.24(61.71-88.48) | 79.77(63.53-98.90) | 0.21(0.15-0.26) |
| Antigua and Barbuda | 9.52(8.72-10.45) | 12.58(11.45-14.04) | 0.81(0.75-0.87) | 64.77(55.63-75.52) | 88.47(74.34-108.12) | 0.81(0.71-0.91) | 83.23(77.56-89.06) | 101.33(92.38-108.43) | 0.69(0.52-0.86) |
| Argentina | 13.29(12.25-14.36) | 13.67(12.39-15.75) | 0.18(0.11-0.24) | 97.51(85.20-108.28) | 103.07(87.56-124.50) | 0.28(0.14-0.41) | 89.33(83.27-94.82) | 82.40(75.58-88.84) | -0.06(-0.14-0.02) |
| Armenia | 9.05(8.21-9.87) | 9.89(8.83-10.93) | 0.31(0.29-0.33) | 72.87(63.21-84.11) | 78.04(65.04-92.97) | 0.25(0.21-0.29) | 65.93(59.91-72.71) | 66.16(58.09-73.29) | -0.20(-0.37--0.03) |
| Australia | 9.72(8.61-10.54) | 12.13(11.03-13.83) | 0.77(0.71-0.83) | 66.74(54.31-78.06) | 89.93(76.29-108.90) | 1.00(0.91-1.08) | 76.06(70.74-81.03) | 77.84(69.23-83.99) | 0.16(0.03-0.29) |
| Austria | 13.34(11.90-14.91) | 17.11(13.81-20.34) | 0.88(0.84-0.91) | 107.34(91.75-126.54) | 146.48(112.37-176.85) | 1.06(1.03-1.10) | 80.84(75.13-86.78) | 85.71(77.36-94.19) | 0.49(0.34-0.65) |
| Azerbaijan | 10.05(8.85-11.36) | 13.12(11.63-14.63) | 0.94(0.90-0.97) | 75.27(62.05-91.33) | 89.25(71.04-106.24) | 0.58(0.53-0.63) | 73.49(59.83-90.65) | 69.12(58.70-80.32) | 0.17(-0.01-0.35) |
| Bahamas | 9.49(8.66-10.39) | 11.06(10.21-11.95) | 0.42(0.34-0.49) | 65.04(54.97-77.16) | 79.90(68.58-93.13) | 0.48(0.33-0.63) | 76.52(70.75-82.38) | 94.53(80.77-111.38) | 0.76(0.61-0.90) |
| Bahrain | 12.28(10.92-13.70) | 16.28(14.53-18.27) | 0.85(0.82-0.89) | 76.74(62.89-91.45) | 116.21(97.46-139.07) | 1.33(1.29-1.38) | 129.91(116.09-143.60) | 107.57(93.12-124.67) | -0.67(-1.01--0.33) |
| Bangladesh | 9.71(8.78-10.85) | 10.54(9.37-11.85) | 0.27(0.19-0.35) | 65.33(52.97-77.96) | 85.18(70.64-102.04) | 0.90(0.81-0.99) | 79.70(65.54-100.73) | 76.47(58.10-104.08) | -0.31(-0.56--0.06) |
| Barbados | 8.92(8.19-9.81) | 11.14(10.07-12.31) | 0.64(0.59-0.69) | 61.91(53.05-72.60) | 80.67(68.70-95.78) | 0.66(0.56-0.76) | 71.48(66.71-76.00) | 86.14(72.23-100.44) | 0.68(0.43-0.93) |
| Belarus | 11.59(10.38-12.77) | 12.42(11.25-13.64) | 0.10(0.01-0.18) | 97.97(81.51-116.21) | 104.37(88.36-121.46) | 0.07(-0.06-0.20) | 80.06(70.42-90.66) | 84.70(73.25-96.61) | -0.12(-0.30-0.07) |
| Belgium | 12.68(11.47-14.41) | 16.24(14.45-18.14) | 0.86(0.83-0.89) | 102.59(89.53-119.37) | 142.14(121.64-163.39) | 1.11(1.07-1.14) | 79.16(73.03-84.94) | 83.62(74.19-91.87) | 0.44(0.37-0.51) |
| Belize | 8.65(7.91-9.46) | 10.46(9.60-11.41) | 0.62(0.55-0.68) | 59.57(50.74-70.19) | 75.70(63.92-88.72) | 0.71(0.61-0.81) | 62.80(58.32-67.52) | 86.23(76.59-95.70) | 0.96(0.45-1.48) |
| Benin | 10.30(9.29-11.40) | 11.48(10.24-12.59) | 0.35(0.31-0.40) | 61.08(50.22-72.26) | 71.95(58.13-86.59) | 0.52(0.44-0.60) | 78.84(66.73-91.89) | 81.54(67.29-97.16) | 0.20(0.14-0.26) |
| Bermuda | 12.26(11.23-13.38) | 12.83(11.66-14.13) | -0.06(-0.14-0.02) | 79.97(67.71-94.43) | 95.39(82.10-108.75) | 0.29(0.16-0.41) | 101.44(80.69-114.91) | 67.53(56.56-81.47) | -1.32(-1.47--1.17) |
| Bhutan | 8.85(7.80-9.99) | 12.28(10.69-14.05) | 1.23(1.18-1.27) | 62.78(49.76-76.87) | 93.80(74.71-113.94) | 1.51(1.44-1.57) | 78.55(58.63-98.84) | 94.86(79.93-109.92) | 0.68(0.65-0.71) |
| Bolivia (Plurinational State of) | 13.08(12.15-14.00) | 19.09(17.03-21.21) | 1.32(1.26-1.38) | 94.86(84.55-105.51) | 160.33(132.70-192.73) | 1.76(1.66-1.86) | 116.20(89.97-139.65) | 120.78(95.43-152.85) | 0.20(0.18-0.23) |
| Bosnia and Herzegovina | 13.25(11.86-14.59) | 12.89(11.36-14.35) | -0.04(-0.06--0.02) | 99.12(80.56-116.87) | 96.97(78.82-118.59) | 0.01(-0.04-0.05) | 96.40(86.15-106.33) | 89.29(73.34-106.10) | -0.38(-0.54--0.21) |
| Botswana | 8.44(7.48-9.49) | 10.36(9.25-11.65) | 0.70(0.63-0.77) | 55.00(43.88-68.28) | 70.42(57.33-85.33) | 0.80(0.69-0.92) | 72.93(58.27-88.91) | 67.92(57.76-81.87) | -0.14(-0.33-0.04) |
| Brazil | 9.29(7.90-10.70) | 10.84(9.38-12.31) | 0.44(0.39-0.49) | 68.18(57.72-80.62) | 90.59(77.08-106.45) | 0.82(0.73-0.90) | 71.83(66.40-76.35) | 74.42(67.13-80.24) | 0.26(0.21-0.31) |
| Brunei Darussalam | 15.66(14.34-17.07) | 16.48(15.03-18.44) | 0.04(-0.11-0.20) | 74.68(61.65-88.47) | 96.97(80.56-118.40) | 0.67(0.49-0.85) | 88.31(51.81-119.19) | 82.50(51.80-108.96) | 0.25(0.08-0.41) |
| Bulgaria | 16.79(15.71-18.13) | 13.46(12.23-14.86) | -0.84(-1.08--0.60) | 125.96(114.04-141.59) | 95.12(79.51-111.83) | -1.13(-1.49--0.76) | 109.56(102.17-117.03) | 103.13(92.57-113.44) | -0.23(-0.35--0.12) |
| Burkina Faso | 10.27(9.24-11.31) | 11.13(10.14-12.26) | 0.24(0.22-0.26) | 59.97(48.35-70.47) | 68.76(57.59-81.13) | 0.41(0.36-0.46) | 81.53(64.35-103.84) | 82.55(65.36-105.11) | 0.16(0.10-0.21) |
| Burundi | 8.03(7.28-8.99) | 8.48(7.58-9.40) | 0.16(0.15-0.17) | 49.54(40.74-60.15) | 56.22(46.66-66.67) | 0.38(0.36-0.41) | 79.85(60.39-100.48) | 75.92(48.92-100.07) | -0.44(-0.54--0.34) |
| Cabo Verde | 8.52(7.49-9.64) | 11.97(10.56-13.45) | 1.33(1.22-1.44) | 59.54(48.94-71.34) | 84.18(68.96-98.81) | 1.30(1.19-1.41) | 65.16(54.36-77.78) | 88.91(70.00-104.39) | 0.78(0.55-1.00) |
| Cambodia | 8.22(7.43-9.00) | 9.94(8.95-11.03) | 0.67(0.63-0.71) | 56.19(46.90-65.82) | 73.94(62.53-86.59) | 0.98(0.91-1.05) | 84.30(71.27-99.48) | 89.74(72.08-108.20) | 0.27(0.16-0.38) |
| Cameroon | 11.39(10.26-12.52) | 12.51(11.23-14.07) | 0.29(0.23-0.34) | 66.09(53.84-77.40) | 77.81(64.03-93.79) | 0.52(0.43-0.61) | 103.19(88.17-120.11) | 101.82(80.97-128.87) | -0.01(-0.05-0.04) |
| Canada | 14.82(14.09-15.70) | 21.05(20.04-21.99) | 1.15(1.05-1.24) | 124.09(116.20-133.52) | 197.61(184.67-209.27) | 1.48(1.35-1.61) | 85.79(79.40-92.19) | 95.86(85.19-104.71) | 0.52(0.36-0.69) |
| Central African Republic | 8.28(7.50-9.21) | 8.50(7.69-9.43) | 0.09(0.06-0.12) | 51.45(42.06-61.31) | 56.25(46.61-67.67) | 0.26(0.19-0.32) | 84.59(69.58-99.12) | 79.98(61.90-97.75) | -0.16(-0.19--0.13) |
| Chad | 9.13(8.22-10.15) | 10.26(9.22-11.54) | 0.43(0.38-0.49) | 55.09(44.53-65.51) | 63.85(52.36-77.78) | 0.57(0.47-0.67) | 65.99(53.19-79.91) | 84.19(67.44-104.28) | 0.91(0.82-0.99) |
| Chile | 12.62(11.68-13.71) | 15.69(13.98-17.35) | 0.91(0.84-0.98) | 86.22(74.94-98.16) | 119.69(100.48-139.52) | 1.37(1.26-1.48) | 88.52(82.62-93.25) | 83.87(76.05-90.21) | 0.13(0.02-0.25) |
| China | 12.83(10.92-14.73) | 24.34(20.67-28.30) | 2.16(2.10-2.21) | 91.77(75.88-109.65) | 245.73(208.28-289.24) | 3.16(3.03-3.30) | 105.26(93.23-116.71) | 107.96(91.10-125.52) | -0.02(-0.13-0.09) |
| Colombia | 8.76(7.87-9.59) | 10.93(9.58-12.50) | 0.54(0.50-0.59) | 67.81(56.54-79.72) | 97.33(81.54-113.01) | 0.95(0.89-1.02) | 67.22(63.10-71.11) | 70.59(61.27-80.84) | -0.15(-0.24--0.06) |
| Comoros | 8.39(7.54-9.40) | 9.03(8.01-10.11) | 0.26(0.24-0.28) | 52.36(43.42-61.81) | 60.55(48.34-73.90) | 0.48(0.45-0.51) | 74.93(60.54-91.62) | 76.23(56.47-101.63) | -0.04(-0.10-0.03) |
| Congo | 9.21(8.24-10.16) | 10.06(8.98-11.17) | 0.31(0.26-0.35) | 58.80(47.39-69.52) | 70.27(57.88-85.90) | 0.54(0.44-0.64) | 90.98(76.61-109.57) | 82.99(68.88-100.95) | -0.42(-0.50--0.34) |
| Cook Islands | 12.41(11.00-14.03) | 12.60(10.80-14.96) | 0.02(-0.04-0.07) | 92.83(76.56-112.44) | 104.23(85.04-128.59) | 0.37(0.30-0.44) | 104.14(89.89-118.74) | 75.58(61.47-96.63) | -1.05(-1.12--0.98) |
| Costa Rica | 10.10(9.02-11.12) | 12.28(10.76-14.14) | 0.57(0.49-0.64) | 80.52(67.00-94.30) | 108.07(91.81-131.66) | 0.84(0.74-0.94) | 66.12(60.71-71.35) | 71.01(63.46-78.95) | 0.18(0.02-0.33) |
| Croatia | 11.68(10.43-13.16) | 12.55(11.13-13.83) | 0.22(0.20-0.24) | 90.61(75.04-108.52) | 99.47(81.05-118.07) | 0.23(0.18-0.28) | 96.32(90.07-102.49) | 83.64(75.63-91.37) | -0.36(-0.46--0.26) |
| Cuba | 7.69(7.06-8.34) | 9.47(8.78-10.30) | 0.70(0.67-0.73) | 52.73(44.88-61.10) | 73.84(64.17-84.84) | 1.10(1.05-1.16) | 63.35(59.57-66.72) | 76.56(68.17-85.49) | 0.61(0.53-0.68) |
| Cyprus | 17.52(15.43-20.37) | 18.03(15.39-21.26) | -0.00(-0.04-0.03) | 127.45(104.07-152.96) | 152.47(124.46-185.55) | 0.51(0.45-0.56) | 179.63(150.92-205.21) | 83.71(73.00-94.85) | -2.55(-2.77--2.32) |
| Czechia | 10.16(9.18-11.05) | 11.55(10.46-12.87) | 0.37(0.35-0.40) | 80.38(66.51-93.30) | 94.67(81.69-110.50) | 0.46(0.42-0.51) | 77.01(72.50-81.63) | 79.58(70.93-87.81) | 0.26(0.18-0.35) |
| Côte d'Ivoire | 11.28(10.24-12.40) | 12.42(11.19-13.71) | 0.32(0.26-0.38) | 65.74(54.50-78.23) | 77.39(63.35-90.29) | 0.54(0.45-0.64) | 95.48(81.69-109.84) | 96.48(80.67-118.18) | 0.09(0.02-0.16) |
| Democratic People's Republic of Korea | 11.26(10.03-12.76) | 17.05(14.77-20.14) | 1.42(1.35-1.50) | 82.90(68.43-99.37) | 161.47(132.14-191.36) | 2.33(2.25-2.41) | 97.73(77.90-118.01) | 103.16(80.72-123.42) | 0.36(0.27-0.45) |
| Democratic Republic of the Congo | 7.70(6.90-8.56) | 8.08(7.21-9.06) | 0.18(0.08-0.28) | 50.94(40.69-62.29) | 58.88(47.74-70.34) | 0.47(0.33-0.60) | 74.76(59.93-89.80) | 73.51(51.71-94.33) | 0.01(-0.16-0.18) |
| Denmark | 10.09(8.94-11.14) | 15.53(13.14-18.04) | 1.52(1.46-1.58) | 81.31(67.97-93.57) | 135.73(108.01-164.14) | 1.82(1.74-1.90) | 61.35(56.98-66.20) | 83.68(75.40-92.05) | 1.17(1.05-1.29) |
| Djibouti | 7.60(6.74-8.52) | 9.13(8.13-10.07) | 0.63(0.60-0.66) | 49.26(39.65-59.31) | 60.72(50.25-71.85) | 0.68(0.62-0.74) | 59.98(43.60-80.22) | 71.34(52.05-102.40) | 0.50(0.42-0.57) |
| Dominica | 9.51(8.68-10.37) | 11.15(10.15-12.13) | 0.47(0.43-0.52) | 61.02(50.47-71.98) | 75.49(63.32-87.82) | 0.55(0.44-0.65) | 103.11(86.95-124.96) | 108.09(90.04-128.81) | 0.00(-0.05-0.06) |
| Dominican Republic | 7.91(7.24-8.76) | 10.28(9.35-11.16) | 0.77(0.73-0.82) | 52.86(44.76-63.46) | 73.62(62.34-85.84) | 0.94(0.83-1.05) | 72.22(62.36-85.22) | 77.10(63.13-94.87) | 0.56(0.37-0.74) |
| Ecuador | 9.90(8.93-11.23) | 16.63(14.57-18.75) | 1.64(1.60-1.68) | 82.42(69.22-100.28) | 154.51(126.86-181.84) | 1.94(1.86-2.02) | 69.47(64.33-74.56) | 88.26(75.12-104.09) | 1.02(0.66-1.38) |
| Egypt | 12.50(11.37-13.95) | 17.28(15.53-19.07) | 1.07(1.04-1.09) | 85.39(70.68-101.49) | 135.75(115.05-155.89) | 1.48(1.44-1.51) | 121.89(109.13-137.19) | 106.47(92.78-123.45) | -0.33(-0.42--0.24) |
| El Salvador | 8.98(8.11-9.85) | 11.90(10.59-13.27) | 0.76(0.66-0.86) | 67.40(57.70-78.91) | 99.89(84.10-114.58) | 1.00(0.85-1.16) | 73.74(66.43-83.61) | 79.65(67.44-92.84) | 0.14(0.03-0.26) |
| Equatorial Guinea | 8.05(7.27-8.91) | 11.09(9.86-12.56) | 1.24(1.18-1.30) | 51.50(42.51-60.00) | 77.75(62.38-92.87) | 1.51(1.47-1.56) | 79.75(64.28-95.76) | 86.33(66.49-111.91) | 0.32(0.26-0.38) |
| Eritrea | 7.81(7.04-8.76) | 8.93(8.03-9.83) | 0.46(0.44-0.49) | 48.91(40.78-59.59) | 58.22(47.90-68.55) | 0.53(0.50-0.56) | 74.09(61.40-88.94) | 86.32(66.70-106.76) | 0.47(0.39-0.55) |
| Estonia | 11.70(10.35-13.43) | 11.53(10.23-13.04) | -0.07(-0.21-0.07) | 109.64(92.14-133.78) | 102.73(87.63-119.43) | -0.20(-0.40-0.00) | 78.44(71.15-86.42) | 75.53(67.26-84.08) | -0.29(-0.38--0.21) |
| Eswatini | 8.76(7.92-9.80) | 10.27(9.14-11.42) | 0.52(0.47-0.57) | 57.62(47.92-69.52) | 68.75(56.22-81.87) | 0.52(0.41-0.63) | 82.82(64.55-104.60) | 95.64(69.27-127.63) | 0.85(0.55-1.16) |
| Ethiopia | 8.33(7.09-9.57) | 8.76(7.47-10.05) | 0.12(0.09-0.15) | 51.47(42.59-61.46) | 60.76(50.31-71.88) | 0.47(0.43-0.52) | 80.97(66.97-104.68) | 62.85(46.40-92.52) | -1.08(-1.20--0.96) |
| Fiji | 12.12(10.73-13.81) | 12.16(10.46-13.78) | 0.11(0.08-0.15) | 84.35(68.85-100.30) | 89.34(70.90-109.39) | 0.27(0.21-0.33) | 100.20(88.47-114.61) | 94.88(78.57-112.75) | -0.44(-0.59--0.29) |
| Finland | 12.27(11.06-13.95) | 17.23(14.77-20.92) | 1.23(1.16-1.30) | 101.28(87.52-118.21) | 152.37(126.25-188.12) | 1.48(1.39-1.58) | 76.83(70.99-82.35) | 92.47(82.15-101.79) | 0.81(0.73-0.89) |
| France | 13.66(12.57-14.85) | 17.49(14.47-19.70) | 0.84(0.79-0.90) | 108.48(94.78-121.46) | 152.06(118.77-177.46) | 1.15(1.08-1.23) | 79.31(73.34-85.02) | 83.16(73.27-92.33) | 0.31(0.23-0.39) |
| Gabon | 10.23(9.25-11.35) | 11.66(10.55-12.99) | 0.41(0.36-0.46) | 65.13(54.36-78.16) | 79.82(67.43-95.74) | 0.60(0.50-0.70) | 105.40(81.47-132.79) | 98.62(81.48-119.27) | -0.28(-0.33--0.23) |
| Gambia | 9.98(8.94-11.14) | 12.29(10.94-13.80) | 0.74(0.69-0.78) | 60.11(48.99-71.54) | 76.05(61.72-91.98) | 0.81(0.73-0.88) | 73.05(56.35-91.72) | 89.75(68.04-109.53) | 0.72(0.66-0.79) |
| Georgia | 10.16(9.13-11.29) | 9.86(9.06-10.80) | -0.15(-0.20--0.10) | 81.89(68.64-97.90) | 74.11(64.71-86.14) | -0.43(-0.51--0.34) | 73.79(65.34-83.95) | 73.79(66.19-81.20) | 0.22(0.08-0.36) |
| Germany | 11.74(11.14-12.20) | 21.53(20.78-22.31) | 1.87(1.74-2.01) | 93.95(87.75-100.28) | 186.45(178.73-194.45) | 2.11(1.94-2.29) | 72.92(67.71-77.83) | 94.95(84.23-104.23) | 0.98(0.78-1.17) |
| Ghana | 8.30(7.36-9.27) | 10.23(9.18-11.48) | 0.66(0.61-0.71) | 54.14(44.18-63.85) | 68.50(56.71-82.57) | 0.68(0.58-0.77) | 63.69(53.80-74.83) | 77.32(63.12-92.30) | 0.73(0.67-0.80) |
| Greece | 13.49(11.92-15.15) | 15.95(14.05-18.35) | 0.49(0.44-0.55) | 110.12(91.92-131.62) | 137.11(116.09-165.04) | 0.64(0.57-0.72) | 79.67(73.47-85.72) | 95.02(86.15-104.60) | 0.54(0.43-0.64) |
| Greenland | 14.95(13.24-16.87) | 16.95(14.95-18.91) | 0.43(0.41-0.45) | 93.74(75.05-114.88) | 116.03(95.35-137.41) | 0.70(0.67-0.73) | 132.40(87.25-174.56) | 116.37(90.96-142.87) | -0.12(-0.28-0.05) |
| Grenada | 8.20(7.54-9.10) | 10.59(9.62-11.69) | 0.72(0.67-0.78) | 54.79(46.67-65.45) | 74.78(63.19-89.49) | 0.86(0.76-0.96) | 68.33(62.50-74.03) | 101.20(90.95-111.03) | 1.22(0.77-1.66) |
| Guam | 13.29(11.53-15.66) | 11.97(10.72-14.51) | -0.30(-0.38--0.23) | 101.71(82.06-125.13) | 98.30(84.12-123.57) | -0.04(-0.16-0.07) | 92.84(75.85-107.12) | 49.16(41.01-57.48) | -1.52(-1.81--1.23) |
| Guatemala | 9.72(8.79-10.81) | 10.92(9.61-12.18) | 0.29(0.21-0.36) | 62.77(52.65-75.95) | 84.49(69.46-101.63) | 0.76(0.63-0.89) | 84.07(80.11-87.93) | 64.76(57.18-72.96) | -1.03(-1.14--0.92) |
| Guinea | 10.35(9.15-11.42) | 11.75(10.60-12.93) | 0.39(0.36-0.43) | 59.00(47.02-70.41) | 70.72(58.70-82.95) | 0.55(0.49-0.61) | 80.55(64.90-98.42) | 94.34(75.43-117.00) | 0.70(0.62-0.77) |
| Guinea-Bissau | 10.90(9.80-12.02) | 11.82(10.64-13.17) | 0.26(0.22-0.30) | 62.05(49.72-74.48) | 70.83(58.11-86.55) | 0.40(0.33-0.47) | 107.21(84.91-128.02) | 111.32(87.92-131.01) | 0.26(0.21-0.30) |
| Guyana | 9.17(8.48-9.96) | 10.28(9.56-11.05) | 0.28(0.23-0.33) | 55.32(46.44-65.12) | 68.98(59.47-80.73) | 0.44(0.33-0.55) | 80.23(73.64-87.03) | 89.04(74.38-106.80) | 0.51(0.31-0.70) |
| Haiti | 9.75(9.07-10.52) | 10.53(9.78-11.40) | 0.16(0.11-0.20) | 53.61(45.01-63.31) | 64.28(54.59-74.31) | 0.40(0.30-0.50) | 118.12(83.03-145.82) | 109.19(81.21-140.97) | -0.13(-0.18--0.08) |
| Honduras | 11.85(10.82-13.07) | 18.00(16.19-20.02) | 1.42(1.31-1.54) | 73.05(60.75-87.12) | 114.74(93.72-137.78) | 1.37(1.23-1.51) | 108.85(94.14-123.92) | 157.67(131.01-188.02) | 1.47(1.31-1.62) |
| Hungary | 9.84(9.01-10.72) | 10.67(9.86-11.78) | 0.33(0.28-0.39) | 75.61(65.44-85.62) | 84.93(74.06-98.56) | 0.48(0.40-0.56) | 75.94(71.78-80.33) | 75.22(67.38-82.78) | 0.13(0.03-0.23) |
| Iceland | 15.72(13.86-17.62) | 20.15(17.31-23.40) | 0.82(0.75-0.89) | 128.23(108.98-150.68) | 172.60(140.72-207.73) | 0.97(0.88-1.06) | 100.82(91.64-108.62) | 109.41(95.68-121.75) | 0.69(0.50-0.87) |
| India | 10.19(8.69-11.77) | 11.91(10.31-13.50) | 0.62(0.55-0.69) | 65.86(54.89-78.69) | 93.90(78.33-111.05) | 1.34(1.25-1.42) | 70.85(56.08-90.98) | 82.31(69.34-95.13) | 0.56(0.40-0.73) |
| Indonesia | 8.56(7.26-9.79) | 11.14(9.58-12.67) | 0.92(0.89-0.95) | 59.98(49.68-71.75) | 79.42(65.71-94.44) | 0.94(0.90-0.98) | 70.73(58.82-85.15) | 93.21(74.95-113.79) | 0.89(0.80-0.98) |
| Iran (Islamic Republic of) | 10.04(8.50-11.56) | 13.05(11.16-14.91) | 0.80(0.75-0.86) | 69.29(57.92-82.47) | 103.68(86.54-122.38) | 1.21(1.14-1.28) | 76.36(49.77-86.97) | 73.79(45.29-83.31) | 0.04(-0.03-0.11) |
| Iraq | 9.43(8.40-10.63) | 13.02(11.40-14.96) | 1.29(1.20-1.38) | 61.89(51.23-74.93) | 95.97(78.21-115.65) | 1.70(1.59-1.81) | 86.99(72.98-105.05) | 100.59(80.61-117.99) | 0.18(0.03-0.34) |
| Ireland | 13.08(11.75-15.06) | 17.64(15.16-19.83) | 1.02(0.95-1.09) | 105.04(90.78-123.06) | 153.91(128.40-176.14) | 1.28(1.19-1.37) | 83.22(77.07-89.00) | 83.04(74.24-91.28) | 0.32(0.22-0.42) |
| Israel | 18.45(16.07-21.73) | 21.87(18.51-26.12) | 0.26(0.11-0.42) | 164.48(138.47-199.54) | 199.71(166.21-240.28) | 0.25(0.05-0.46) | 93.36(84.97-101.84) | 90.60(79.74-102.00) | -0.11(-0.19--0.03) |
| Italy | 17.47(14.88-19.99) | 13.93(11.83-15.96) | -1.16(-1.37--0.95) | 160.72(136.22-187.75) | 113.18(93.93-132.49) | -1.71(-2.00--1.43) | 90.15(81.45-98.50) | 79.29(69.96-86.84) | -0.45(-0.57--0.33) |
| Jamaica | 8.19(7.45-8.89) | 9.53(8.72-10.62) | 0.48(0.41-0.54) | 58.69(49.05-68.95) | 73.21(62.13-88.59) | 0.64(0.54-0.75) | 62.77(58.42-66.60) | 77.66(64.15-92.88) | 0.73(0.49-0.97) |
| Japan | 8.01(6.83-9.29) | 7.28(6.29-8.28) | 0.20(0.05-0.34) | 65.50(55.39-77.64) | 52.95(44.63-62.40) | 0.07(-0.18-0.32) | 55.56(50.87-59.42) | 58.54(50.93-63.30) | 0.50(0.40-0.61) |
| Jordan | 10.68(9.69-11.79) | 11.35(10.38-12.42) | 0.36(0.29-0.42) | 62.20(51.57-72.41) | 75.66(65.65-86.24) | 1.00(0.86-1.14) | 87.13(72.63-104.75) | 66.34(55.37-79.01) | -0.99(-1.12--0.86) |
| Kazakhstan | 10.13(8.87-11.48) | 12.56(11.32-13.76) | 0.86(0.79-0.94) | 76.31(61.77-91.57) | 93.39(79.56-106.97) | 0.82(0.67-0.96) | 68.60(63.65-74.33) | 89.02(80.08-97.70) | 0.64(0.47-0.80) |
| Kenya | 7.89(6.67-9.15) | 9.15(7.80-10.38) | 0.50(0.45-0.56) | 53.00(44.00-63.38) | 62.55(52.25-73.85) | 0.50(0.43-0.56) | 55.82(44.43-71.31) | 72.45(56.43-91.29) | 1.02(0.95-1.10) |
| Kiribati | 13.05(11.78-14.44) | 13.58(12.45-15.02) | 0.18(0.15-0.21) | 78.84(64.80-94.37) | 85.45(71.40-103.22) | 0.32(0.26-0.38) | 101.05(85.28-115.84) | 100.16(84.48-120.82) | -0.05(-0.08--0.03) |
| Kuwait | 9.65(8.72-10.89) | 11.31(9.40-13.23) | 0.50(0.43-0.57) | 72.71(61.89-85.21) | 96.39(77.51-117.02) | 0.91(0.83-1.00) | 105.16(94.27-114.17) | 65.22(55.06-76.20) | -0.79(-1.31--0.26) |
| Kyrgyzstan | 8.98(8.09-9.89) | 8.89(7.92-9.86) | -0.03(-0.11-0.05) | 70.61(58.98-83.69) | 69.52(58.08-82.40) | -0.04(-0.18-0.10) | 63.96(56.12-72.68) | 56.00(48.76-63.93) | -0.46(-0.68--0.24) |
| Lao People's Democratic Republic | 9.27(8.46-10.06) | 10.44(9.36-11.60) | 0.39(0.35-0.43) | 60.07(49.85-70.96) | 74.26(62.19-89.27) | 0.67(0.61-0.73) | 89.05(71.59-110.41) | 88.44(71.31-109.38) | -0.13(-0.18--0.08) |
| Latvia | 11.18(10.17-12.31) | 11.04(10.00-12.28) | -0.21(-0.31--0.12) | 94.12(80.94-108.12) | 93.38(80.77-113.20) | -0.26(-0.38--0.14) | 79.98(72.27-87.58) | 76.54(68.33-84.43) | -0.28(-0.43--0.13) |
| Lebanon | 9.57(8.57-10.72) | 11.54(10.16-13.01) | 0.65(0.62-0.68) | 66.02(54.51-77.53) | 92.56(77.33-111.38) | 1.16(1.10-1.22) | 87.94(58.91-113.31) | 67.12(57.75-78.28) | -0.54(-0.69--0.38) |
| Lesotho | 7.06(6.26-7.91) | 9.20(8.28-10.28) | 0.85(0.81-0.90) | 47.71(39.02-56.89) | 60.17(50.25-71.89) | 0.62(0.54-0.71) | 59.71(49.03-73.24) | 85.43(65.42-105.86) | 1.67(1.40-1.94) |
| Liberia | 9.78(8.86-10.84) | 10.49(9.34-11.52) | 0.20(0.16-0.24) | 59.50(48.64-72.95) | 69.61(56.57-80.55) | 0.51(0.46-0.57) | 81.92(70.30-95.23) | 82.37(60.77-105.49) | 0.11(0.03-0.19) |
| Libya | 9.00(8.09-10.04) | 11.58(10.30-12.86) | 0.92(0.85-0.98) | 62.55(52.06-72.67) | 91.60(76.88-106.18) | 1.37(1.28-1.47) | 80.12(61.23-103.01) | 108.34(73.78-170.84) | 1.43(1.27-1.58) |
| Lithuania | 9.65(8.69-11.25) | 10.61(9.40-11.62) | 0.12(0.04-0.19) | 88.01(74.61-105.40) | 91.99(77.61-106.57) | -0.11(-0.21--0.01) | 64.75(58.86-71.17) | 73.39(65.59-80.94) | 0.46(0.32-0.59) |
| Luxembourg | 14.62(12.92-16.89) | 18.52(15.91-21.21) | 0.80(0.76-0.84) | 115.92(97.56-140.60) | 157.37(130.18-186.49) | 1.06(1.01-1.11) | 95.82(89.43-102.73) | 94.40(84.21-104.90) | 0.36(0.22-0.50) |
| Madagascar | 6.89(6.13-7.84) | 7.49(6.61-8.37) | 0.28(0.27-0.30) | 44.79(36.50-54.90) | 53.03(42.63-63.24) | 0.52(0.50-0.54) | 59.88(49.05-74.06) | 57.65(42.67-78.54) | -0.11(-0.18--0.04) |
| Malawi | 7.93(7.16-8.79) | 8.56(7.62-9.46) | 0.23(0.21-0.26) | 49.79(41.21-58.49) | 57.56(47.63-67.67) | 0.41(0.36-0.47) | 64.69(49.61-79.75) | 73.36(50.78-99.39) | 0.28(0.17-0.40) |
| Malaysia | 9.81(8.83-10.88) | 11.92(10.55-13.60) | 0.71(0.66-0.75) | 69.71(57.05-82.83) | 92.24(76.38-114.51) | 0.99(0.95-1.04) | 70.13(62.21-79.04) | 85.64(76.95-95.91) | 0.60(0.42-0.78) |
| Maldives | 11.64(10.56-12.79) | 12.66(11.26-14.40) | 0.27(0.25-0.30) | 76.15(62.81-89.68) | 93.81(78.39-113.67) | 0.66(0.59-0.73) | 99.26(80.15-113.66) | 69.03(58.24-79.93) | -1.41(-1.49--1.33) |
| Mali | 8.83(8.00-9.76) | 9.62(8.72-10.56) | 0.26(0.24-0.29) | 54.86(45.76-64.30) | 62.59(52.48-74.78) | 0.41(0.37-0.45) | 89.35(75.66-104.16) | 93.83(77.67-110.88) | 0.35(0.26-0.45) |
| Malta | 13.67(11.96-15.72) | 17.55(15.74-20.12) | 0.84(0.78-0.90) | 107.09(87.84-130.17) | 148.84(126.92-175.41) | 1.14(1.04-1.24) | 91.56(84.09-98.26) | 88.57(78.57-99.08) | -0.00(-0.23-0.22) |
| Marshall Islands | 13.18(11.55-14.74) | 14.23(12.59-16.17) | 0.33(0.28-0.37) | 87.30(70.66-105.93) | 98.50(80.34-120.15) | 0.49(0.41-0.57) | 121.76(105.84-137.75) | 116.75(95.37-142.42) | -0.10(-0.15--0.04) |
| Mauritania | 10.39(9.36-11.46) | 11.30(10.06-12.96) | 0.24(0.19-0.29) | 60.52(49.56-72.41) | 73.30(59.75-89.70) | 0.56(0.49-0.63) | 80.11(63.36-97.64) | 80.31(60.07-104.59) | 0.00(-0.14-0.15) |
| Mauritius | 9.59(8.72-10.74) | 11.10(9.67-12.37) | 0.35(0.30-0.39) | 71.17(61.04-83.79) | 89.00(72.44-104.78) | 0.54(0.47-0.60) | 79.13(74.37-83.37) | 76.26(69.76-81.44) | -0.20(-0.30--0.10) |
| Mexico | 9.48(8.16-10.87) | 12.35(10.66-14.00) | 0.65(0.55-0.75) | 67.38(57.19-79.50) | 100.92(85.60-118.59) | 0.97(0.82-1.12) | 80.79(76.85-84.69) | 78.05(69.74-87.30) | -0.10(-0.18--0.03) |
| Micronesia (Federated States of) | 12.57(11.41-13.97) | 13.47(12.10-14.87) | 0.34(0.31-0.38) | 79.94(67.08-95.44) | 90.39(73.38-107.74) | 0.58(0.52-0.64) | 116.81(98.25-135.37) | 106.16(86.03-130.30) | -0.32(-0.37--0.27) |
| Monaco | 14.10(12.16-16.26) | 18.21(15.46-21.81) | 0.86(0.82-0.89) | 115.42(94.19-136.78) | 157.08(127.95-193.96) | 1.02(0.97-1.07) | 90.66(72.48-109.47) | 108.74(87.36-129.19) | 0.74(0.65-0.82) |
| Mongolia | 9.69(8.81-10.82) | 9.05(8.15-10.26) | -0.13(-0.18--0.08) | 67.66(56.20-83.44) | 67.51(56.11-81.27) | 0.13(0.06-0.21) | 69.59(60.40-80.72) | 62.70(53.55-72.15) | -0.56(-0.68--0.45) |
| Montenegro | 12.52(11.06-14.21) | 13.26(11.79-14.55) | 0.30(0.26-0.34) | 98.14(80.48-115.09) | 100.84(83.69-115.95) | 0.22(0.14-0.30) | 84.72(73.21-97.91) | 107.06(93.44-124.53) | 0.81(0.56-1.06) |
| Morocco | 9.09(8.15-10.30) | 13.00(11.63-14.38) | 1.35(1.29-1.42) | 60.03(49.05-71.85) | 95.94(78.94-113.87) | 1.73(1.65-1.82) | 76.76(63.79-90.87) | 101.46(80.79-118.67) | 1.11(1.02-1.20) |
| Mozambique | 7.76(6.99-8.76) | 8.74(7.83-9.76) | 0.35(0.33-0.37) | 47.20(38.77-58.28) | 56.20(46.16-67.47) | 0.45(0.41-0.50) | 66.12(51.52-85.60) | 80.23(56.73-111.88) | 0.98(0.85-1.11) |
| Myanmar | 8.33(7.53-9.17) | 10.29(9.30-11.28) | 0.75(0.72-0.78) | 55.62(45.94-64.88) | 75.54(62.89-87.26) | 1.04(0.98-1.11) | 75.41(63.19-90.40) | 83.67(68.70-104.62) | 0.20(0.11-0.30) |
| Namibia | 8.54(7.62-9.50) | 10.00(8.94-11.14) | 0.54(0.49-0.59) | 55.22(45.16-66.56) | 68.80(56.30-81.42) | 0.69(0.59-0.79) | 72.05(60.46-86.43) | 85.37(70.52-101.68) | 0.56(0.43-0.69) |
| Nauru | 15.43(13.89-17.31) | 14.90(13.35-16.36) | -0.05(-0.12-0.03) | 95.65(77.28-118.94) | 99.61(83.34-117.88) | 0.24(0.09-0.38) | 157.22(120.73-198.90) | 132.58(99.42-168.86) | -0.55(-0.58--0.52) |
| Nepal | 7.67(6.86-8.63) | 10.78(9.56-12.20) | 1.25(1.21-1.30) | 52.96(42.78-64.32) | 83.38(68.53-99.79) | 1.60(1.55-1.66) | 69.03(56.46-84.42) | 85.36(69.42-103.49) | 0.87(0.66-1.09) |
| Netherlands | 18.78(16.77-20.45) | 19.11(17.14-21.25) | -0.27(-0.50--0.03) | 162.18(143.17-181.79) | 168.81(148.57-192.20) | -0.31(-0.60--0.02) | 101.15(91.98-109.46) | 94.29(84.28-103.13) | -0.08(-0.16-0.01) |
| New Zealand | 8.93(7.49-10.42) | 10.17(8.67-11.71) | 0.48(0.45-0.51) | 59.00(48.41-71.67) | 68.81(56.87-83.28) | 0.52(0.48-0.56) | 69.38(64.38-73.72) | 77.69(69.33-83.37) | 0.48(0.41-0.55) |
| Nicaragua | 10.88(9.87-11.81) | 14.89(13.29-16.43) | 0.92(0.84-1.00) | 76.64(65.09-87.40) | 111.76(91.94-130.73) | 1.04(0.93-1.14) | 61.60(54.96-70.39) | 64.24(54.19-73.81) | 0.29(0.07-0.51) |
| Niger | 9.08(8.09-10.08) | 9.74(8.67-10.96) | 0.29(0.25-0.32) | 55.49(44.29-67.09) | 61.62(50.56-75.17) | 0.46(0.39-0.52) | 72.21(56.34-88.12) | 78.13(62.95-97.37) | 0.44(0.37-0.50) |
| Nigeria | 9.94(8.40-11.51) | 12.49(10.66-14.29) | 0.81(0.76-0.86) | 61.63(51.11-73.86) | 78.46(64.74-93.47) | 0.86(0.78-0.94) | 78.78(67.27-91.40) | 90.34(75.80-103.95) | 0.65(0.55-0.74) |
| Niue | 12.96(11.43-14.51) | 13.26(11.60-15.03) | 0.08(0.01-0.14) | 93.29(76.25-112.81) | 102.87(87.76-125.12) | 0.40(0.31-0.49) | 112.04(96.46-127.29) | 103.73(88.56-118.03) | -0.31(-0.34--0.28) |
| North Macedonia | 13.17(11.73-14.72) | 14.93(13.51-16.55) | 0.44(0.38-0.49) | 92.23(76.15-110.20) | 103.48(86.96-121.51) | 0.39(0.30-0.48) | 96.27(86.52-107.05) | 105.22(89.52-121.32) | 0.18(-0.07-0.43) |
| Northern Mariana Islands | 13.42(11.90-15.17) | 13.87(12.32-15.51) | -0.11(-0.23--0.00) | 101.06(84.70-118.92) | 109.02(91.74-126.85) | 0.01(-0.15-0.16) | 100.87(80.14-121.63) | 90.85(76.92-104.49) | -0.37(-0.43--0.30) |
| Norway | 5.02(4.30-5.76) | 12.02(10.27-13.80) | 3.39(3.14-3.65) | 26.38(22.30-31.01) | 96.32(80.36-113.97) | 5.04(4.64-5.45) | 63.95(59.33-66.83) | 89.47(80.77-96.52) | 1.53(1.36-1.70) |
| Oman | 13.44(11.78-15.05) | 20.18(17.24-22.94) | 1.40(1.32-1.48) | 80.21(63.34-95.99) | 135.68(106.19-161.63) | 1.79(1.72-1.85) | 100.54(80.01-124.39) | 102.33(86.16-119.24) | 0.49(0.26-0.71) |
| Pakistan | 10.50(9.02-12.15) | 12.45(10.71-14.16) | 0.62(0.59-0.65) | 68.53(57.06-81.90) | 89.08(73.86-105.65) | 0.91(0.85-0.97) | 82.58(69.70-96.84) | 103.02(87.36-122.14) | 0.56(0.41-0.70) |
| Palau | 15.29(13.62-17.71) | 15.36(13.44-17.61) | -0.00(-0.06-0.06) | 106.44(87.07-128.71) | 114.71(93.39-146.40) | 0.26(0.18-0.34) | 101.98(88.28-118.34) | 93.27(78.44-110.87) | -0.13(-0.21--0.06) |
| Palestine | 11.78(10.41-13.09) | 14.27(12.61-15.91) | 0.68(0.61-0.75) | 72.91(58.42-86.81) | 101.51(84.61-118.72) | 1.13(1.03-1.24) | 98.97(83.97-117.10) | 84.08(73.33-93.93) | -0.57(-0.81--0.34) |
| Panama | 9.22(8.38-10.07) | 12.52(11.03-14.31) | 0.82(0.74-0.91) | 73.77(62.17-84.60) | 108.73(90.78-127.08) | 0.97(0.86-1.09) | 63.46(58.71-68.20) | 74.36(60.99-87.59) | 0.46(0.28-0.64) |
| Papua New Guinea | 9.74(8.66-11.12) | 10.53(9.19-11.93) | 0.35(0.30-0.40) | 70.00(57.50-85.75) | 77.56(62.84-91.54) | 0.42(0.34-0.49) | 91.79(71.44-119.76) | 86.40(66.38-116.91) | -0.18(-0.24--0.13) |
| Paraguay | 8.60(7.67-9.66) | 10.34(9.14-11.46) | 0.56(0.52-0.60) | 67.36(55.55-80.94) | 88.44(74.49-101.66) | 0.80(0.73-0.86) | 55.74(48.14-63.99) | 79.30(64.44-96.73) | 1.35(1.26-1.45) |
| Peru | 11.32(10.09-12.63) | 16.21(14.24-18.60) | 1.15(1.13-1.17) | 92.93(78.92-110.01) | 160.74(134.43-194.05) | 1.74(1.70-1.78) | 89.59(79.12-100.87) | 97.23(79.86-117.53) | 0.18(-0.01-0.37) |
| Philippines | 9.45(8.05-10.84) | 9.95(8.50-11.30) | 0.13(0.06-0.19) | 65.83(54.50-78.88) | 74.67(62.35-88.47) | 0.39(0.30-0.48) | 67.37(59.47-75.98) | 76.87(66.61-87.67) | 0.62(0.57-0.67) |
| Poland | 11.77(9.99-13.66) | 12.56(11.33-13.89) | 0.19(0.18-0.21) | 89.15(73.62-107.50) | 103.34(93.22-115.62) | 0.43(0.39-0.47) | 80.71(75.92-85.38) | 84.91(76.58-93.04) | 0.03(-0.04-0.09) |
| Portugal | 10.15(8.92-11.42) | 13.54(11.79-15.25) | 0.95(0.91-0.99) | 77.99(64.74-91.50) | 112.31(90.59-132.41) | 1.23(1.15-1.31) | 67.48(62.63-72.25) | 73.37(65.94-79.83) | 0.52(0.39-0.64) |
| Puerto Rico | 8.98(8.05-9.57) | 10.01(9.01-10.98) | 0.33(0.27-0.40) | 66.44(56.32-76.12) | 82.74(68.25-95.41) | 0.68(0.59-0.78) | 77.46(73.21-81.50) | 70.91(61.06-81.34) | -0.31(-0.37--0.25) |
| Qatar | 16.92(15.02-19.09) | 24.22(21.16-27.95) | 1.28(1.22-1.33) | 101.06(80.21-122.20) | 161.22(128.85-198.10) | 1.67(1.58-1.76) | 199.42(173.24-227.31) | 115.82(94.76-141.07) | -1.92(-2.46--1.37) |
| Republic of Korea | 7.46(6.67-8.36) | 11.01(9.77-12.19) | 1.22(1.15-1.29) | 50.86(41.71-61.12) | 88.74(73.90-103.16) | 1.72(1.60-1.85) | 73.67(65.23-94.34) | 68.24(55.55-78.91) | 0.03(-0.07-0.14) |
| Republic of Moldova | 10.87(9.66-12.27) | 8.82(7.80-10.04) | -0.89(-0.95--0.83) | 91.41(74.83-110.09) | 78.97(64.58-93.66) | -0.71(-0.79--0.62) | 77.66(71.88-83.81) | 56.80(51.56-62.63) | -1.31(-1.49--1.12) |
| Romania | 10.48(9.54-11.38) | 11.54(10.54-12.54) | 0.28(0.25-0.31) | 74.90(62.42-86.44) | 87.87(74.46-99.82) | 0.46(0.40-0.52) | 81.20(76.58-85.86) | 87.21(79.36-95.23) | 0.16(0.07-0.24) |
| Russian Federation | 10.38(8.86-12.04) | 10.77(9.24-12.33) | 0.06(-0.03-0.15) | 87.24(72.21-105.20) | 90.27(75.14-107.45) | 0.03(-0.11-0.18) | 70.58(66.35-75.23) | 76.19(70.04-82.70) | 0.09(-0.01-0.19) |
| Rwanda | 8.13(7.30-9.02) | 8.32(7.48-9.31) | 0.12(0.09-0.16) | 50.20(41.59-60.21) | 56.76(46.16-68.48) | 0.47(0.41-0.53) | 92.77(69.00-125.29) | 78.75(54.20-104.50) | -1.04(-1.25--0.84) |
| Saint Kitts and Nevis | 10.38(9.48-11.32) | 11.71(10.67-12.87) | 0.34(0.30-0.38) | 62.09(52.22-73.28) | 79.69(67.28-95.61) | 0.71(0.63-0.80) | 137.08(129.87-143.88) | 143.73(125.16-159.25) | 0.61(0.43-0.79) |
| Saint Lucia | 10.41(9.63-11.49) | 11.53(10.43-12.64) | 0.27(0.22-0.31) | 64.49(54.36-76.44) | 79.88(65.70-94.04) | 0.51(0.42-0.61) | 103.93(98.95-109.25) | 95.92(81.33-110.32) | -0.69(-0.94--0.43) |
| Saint Vincent and the Grenadines | 8.42(7.69-9.10) | 10.17(9.37-11.15) | 0.58(0.55-0.61) | 56.37(46.78-66.18) | 70.48(59.66-83.05) | 0.65(0.59-0.71) | 76.70(71.35-81.41) | 88.55(80.31-97.69) | 0.54(0.42-0.66) |
| Samoa | 11.77(10.39-13.17) | 12.44(11.07-13.81) | 0.26(0.20-0.31) | 84.58(68.93-100.35) | 95.54(79.97-112.59) | 0.51(0.42-0.59) | 103.03(85.14-127.08) | 94.89(79.08-120.01) | -0.24(-0.30--0.18) |
| San Marino | 13.20(11.37-15.19) | 14.55(11.98-17.82) | 0.24(0.18-0.31) | 119.54(100.38-140.07) | 136.33(109.86-169.22) | 0.31(0.22-0.40) | 63.13(54.38-71.83) | 43.51(33.07-54.95) | -0.60(-0.85--0.35) |
| Sao Tome and Principe | 10.11(8.96-11.45) | 12.97(11.59-14.55) | 0.88(0.83-0.93) | 64.02(51.91-76.39) | 84.98(70.21-102.83) | 0.97(0.88-1.05) | 78.02(69.11-86.30) | 94.99(80.14-109.54) | 0.84(0.76-0.92) |
| Saudi Arabia | 14.13(12.48-15.76) | 18.89(16.58-21.72) | 0.90(0.85-0.96) | 81.20(64.61-97.59) | 133.32(109.53-162.56) | 1.57(1.50-1.64) | 141.09(110.57-174.61) | 138.08(116.74-165.19) | -0.17(-0.23--0.11) |
| Senegal | 10.77(9.69-11.77) | 12.60(11.46-13.96) | 0.47(0.44-0.51) | 62.34(50.80-74.96) | 78.18(65.10-93.92) | 0.63(0.56-0.70) | 79.74(66.30-94.08) | 94.91(76.34-114.88) | 0.63(0.55-0.70) |
| Serbia | 11.60(10.43-12.61) | 12.85(11.31-14.21) | 0.42(0.39-0.45) | 85.55(71.59-99.21) | 94.15(74.69-109.98) | 0.45(0.41-0.50) | 110.09(98.17-121.66) | 83.40(73.46-92.92) | -0.91(-0.99--0.84) |
| Seychelles | 12.67(11.51-14.15) | 15.31(13.85-17.15) | 0.60(0.57-0.63) | 83.73(70.96-99.96) | 105.50(89.28-125.54) | 0.72(0.64-0.79) | 121.50(104.98-135.20) | 110.54(90.89-129.22) | -0.12(-0.27-0.02) |
| Sierra Leone | 9.30(8.37-10.17) | 10.33(9.26-11.30) | 0.33(0.27-0.39) | 55.63(45.51-66.05) | 65.48(54.10-76.67) | 0.49(0.37-0.60) | 73.56(62.21-86.66) | 78.84(63.20-97.12) | 0.39(0.31-0.47) |
| Singapore | 8.47(7.75-9.31) | 10.20(8.96-11.52) | 0.60(0.50-0.69) | 61.47(52.62-72.10) | 81.35(68.38-96.88) | 0.90(0.74-1.06) | 67.90(63.88-71.55) | 55.65(50.09-60.55) | -0.39(-0.55--0.22) |
| Slovakia | 10.41(9.21-11.48) | 10.63(9.53-11.89) | 0.00(-0.04-0.04) | 81.08(67.30-95.74) | 85.31(71.73-100.92) | 0.09(0.05-0.14) | 82.77(75.28-91.78) | 70.38(61.50-79.00) | -0.40(-0.47--0.33) |
| Slovenia | 11.81(10.76-12.94) | 12.22(10.59-13.49) | 0.09(0.04-0.13) | 96.52(83.60-111.39) | 101.16(84.51-116.38) | 0.11(0.05-0.18) | 80.03(74.57-85.82) | 79.58(69.24-88.80) | -0.15(-0.27--0.03) |
| Solomon Islands | 12.00(10.65-13.45) | 12.54(11.20-14.07) | 0.18(0.14-0.23) | 78.43(63.40-94.50) | 86.37(70.12-104.19) | 0.36(0.28-0.43) | 103.76(80.56-125.74) | 95.39(77.35-118.06) | -0.33(-0.39--0.27) |
| Somalia | 7.40(6.69-8.28) | 7.52(6.72-8.44) | 0.03(-0.00-0.07) | 46.22(37.89-56.43) | 49.02(40.07-60.17) | 0.12(0.07-0.17) | 68.97(51.03-88.27) | 68.52(45.98-93.41) | 0.08(0.04-0.12) |
| South Africa | 7.87(6.69-9.10) | 9.65(8.20-11.07) | 0.67(0.63-0.71) | 54.97(46.02-66.14) | 68.26(57.02-81.00) | 0.68(0.60-0.75) | 50.05(41.83-60.79) | 69.96(62.41-76.09) | 1.14(0.84-1.44) |
| South Sudan | 7.74(6.88-8.70) | 7.95(6.90-8.96) | 0.06(0.05-0.08) | 49.60(40.35-60.75) | 54.11(42.13-64.72) | 0.27(0.25-0.28) | 74.88(61.06-92.31) | 77.88(60.33-101.75) | 0.06(0.01-0.12) |
| Spain | 17.12(15.84-19.10) | 19.97(17.46-22.74) | 0.42(0.19-0.65) | 144.93(131.43-160.98) | 177.82(146.98-207.53) | 0.54(0.27-0.80) | 87.36(79.67-94.12) | 90.67(79.96-100.55) | 0.32(0.20-0.44) |
| Sri Lanka | 9.59(8.52-10.69) | 11.42(9.91-13.18) | 0.58(0.53-0.62) | 70.87(57.72-83.24) | 93.13(75.38-113.90) | 0.93(0.87-0.99) | 81.23(73.75-90.42) | 72.69(54.83-90.67) | 0.01(-0.14-0.17) |
| Sudan | 9.44(8.52-10.44) | 11.83(10.37-13.29) | 0.78(0.73-0.83) | 60.18(50.06-71.25) | 88.81(72.19-107.92) | 1.31(1.21-1.41) | 77.63(63.78-99.20) | 79.13(64.03-101.20) | 0.09(0.03-0.15) |
| Suriname | 7.30(6.66-7.98) | 8.92(8.10-9.79) | 0.63(0.59-0.67) | 49.47(41.52-58.41) | 65.30(53.56-77.34) | 0.79(0.72-0.85) | 66.11(59.65-73.58) | 67.87(53.68-81.80) | 0.35(0.19-0.51) |
| Sweden | 9.37(8.00-10.89) | 14.74(12.36-17.05) | 1.60(1.54-1.67) | 76.54(63.90-91.20) | 123.14(101.86-145.94) | 1.61(1.55-1.68) | 73.21(67.59-77.86) | 76.92(68.18-85.71) | 0.20(-0.00-0.41) |
| Switzerland | 13.67(11.81-15.56) | 16.95(14.49-19.67) | 0.73(0.65-0.80) | 112.59(94.30-131.31) | 148.82(122.58-176.94) | 0.94(0.84-1.03) | 78.66(71.43-85.12) | 79.34(69.30-88.16) | 0.29(0.18-0.40) |
| Syrian Arab Republic | 9.77(8.81-11.02) | 12.93(11.49-14.59) | 1.03(0.95-1.10) | 62.28(52.16-74.69) | 95.39(79.21-114.17) | 1.57(1.46-1.69) | 92.36(77.95-111.08) | 97.10(79.38-118.88) | 0.03(-0.07-0.13) |
| Taiwan (Province of China) | 9.93(9.36-10.42) | 21.29(20.47-22.24) | 2.89(2.51-3.27) | 64.22(58.04-69.82) | 194.47(185.60-204.06) | 4.22(3.65-4.80) | 90.10(84.98-94.30) | 98.99(88.36-108.57) | 0.51(0.40-0.61) |
| Tajikistan | 12.43(10.83-14.08) | 17.55(15.63-19.72) | 1.20(1.12-1.28) | 83.53(66.25-101.41) | 102.83(82.40-123.82) | 0.66(0.60-0.72) | 123.32(91.16-184.66) | 109.60(92.10-129.51) | -0.56(-0.83--0.28) |
| Thailand | 11.27(10.20-12.50) | 11.64(10.46-12.92) | 0.12(0.09-0.15) | 75.09(62.87-91.05) | 90.70(77.70-105.02) | 0.65(0.61-0.69) | 95.97(82.11-110.23) | 86.35(70.57-106.25) | -0.76(-0.91--0.60) |
| Timor-Leste | 8.52(7.68-9.42) | 10.66(9.54-11.88) | 0.80(0.77-0.83) | 57.46(47.97-67.50) | 74.20(60.88-89.79) | 0.91(0.86-0.95) | 66.11(52.40-81.13) | 74.46(59.41-91.76) | 0.54(0.42-0.65) |
| Togo | 9.48(8.56-10.44) | 10.57(9.13-12.04) | 0.33(0.27-0.39) | 58.00(47.95-68.89) | 68.15(53.05-83.61) | 0.49(0.40-0.58) | 69.59(55.64-84.96) | 82.92(63.27-106.76) | 0.63(0.56-0.70) |
| Tokelau | 11.56(10.23-12.98) | 11.87(10.64-13.34) | 0.12(0.06-0.18) | 82.37(68.04-99.49) | 93.62(79.30-111.66) | 0.50(0.42-0.58) | 114.82(95.00-142.97) | 93.44(75.35-117.98) | -0.70(-0.72--0.68) |
| Tonga | 10.05(8.72-11.40) | 10.89(9.56-12.56) | 0.32(0.26-0.37) | 76.53(62.17-90.30) | 86.80(73.11-103.52) | 0.48(0.40-0.57) | 78.96(62.83-106.56) | 79.97(62.04-108.32) | 0.12(0.02-0.23) |
| Trinidad and Tobago | 8.36(7.67-9.06) | 9.15(8.35-10.45) | 0.21(0.16-0.27) | 56.42(48.16-65.70) | 69.08(57.38-82.40) | 0.54(0.44-0.64) | 77.28(73.39-80.98) | 72.41(59.81-86.32) | -0.26(-0.38--0.14) |
| Tunisia | 9.72(8.50-11.17) | 12.26(10.56-13.69) | 0.82(0.78-0.85) | 66.31(53.53-81.53) | 96.25(78.01-113.70) | 1.30(1.25-1.36) | 68.07(56.08-84.87) | 73.27(56.23-93.15) | 0.23(0.15-0.32) |
| Turkmenistan | 7.88(7.00-8.79) | 8.30(7.45-9.10) | 0.25(0.19-0.32) | 63.74(52.61-77.26) | 66.75(56.03-76.70) | 0.25(0.16-0.35) | 58.30(54.04-63.14) | 65.31(53.79-78.69) | 0.09(-0.09-0.28) |
| Tuvalu | 12.38(11.30-13.67) | 12.89(11.63-14.30) | 0.20(0.15-0.24) | 82.58(69.98-98.81) | 93.21(79.33-108.31) | 0.50(0.44-0.56) | 117.81(98.92-136.99) | 104.14(85.59-128.22) | -0.38(-0.40--0.35) |
| Turkey | 9.60(8.63-10.70) | 12.97(11.36-14.88) | 1.02(0.97-1.07) | 63.18(52.73-76.15) | 98.93(81.08-119.83) | 0.58(0.50-0.67) | 101.88(88.33-115.61) | 86.75(73.01-100.68) | -0.45(-0.68--0.22) |
| Uganda | 8.18(7.31-9.15) | 9.05(8.06-10.00) | 0.40(0.35-0.45) | 52.06(42.67-62.08) | 60.94(49.96-71.11) | 1.56(1.48-1.63) | 69.37(49.46-92.67) | 75.14(53.42-96.15) | 0.06(-0.06-0.18) |
| Ukraine | 11.27(9.66-13.14) | 11.36(9.68-13.00) | -0.04(-0.12-0.03) | 98.16(80.76-118.28) | 95.78(79.74-113.30) | -0.16(-0.26--0.06) | 68.87(61.04-77.43) | 72.79(59.17-87.61) | -0.11(-0.25-0.02) |
| United Arab Emirates | 17.34(15.64-19.16) | 19.56(17.31-22.48) | 0.25(0.12-0.38) | 97.37(78.61-115.61) | 140.65(112.67-173.95) | 1.13(0.95-1.30) | 103.15(74.98-132.79) | 94.92(78.82-111.52) | 1.50(0.93-2.09) |
| United Kingdom | 14.45(12.55-16.53) | 16.60(14.43-18.77) | 0.50(0.39-0.60) | 128.91(110.51-150.75) | 141.10(121.20-163.16) | 0.32(0.12-0.51) | 92.61(85.99-99.06) | 92.01(83.89-99.76) | 0.42(0.26-0.59) |
| United Republic of Tanzania | 7.44(6.70-8.23) | 8.77(8.21-9.45) | 0.49(0.47-0.52) | 47.11(39.42-54.72) | 59.68(53.40-66.13) | 0.67(0.63-0.71) | 65.98(51.81-81.99) | 68.41(51.64-92.72) | 0.02(-0.03-0.07) |
| United States of America | 11.13(9.39-12.89) | 14.25(13.04-15.52) | 0.72(0.67-0.78) | 86.92(72.15-104.03) | 111.85(102.83-121.45) | 0.75(0.69-0.80) | 68.05(62.20-73.13) | 97.67(87.38-104.70) | 1.22(1.13-1.30) |
| United States Virgin Islands | 13.38(12.18-14.62) | 15.89(14.59-17.24) | 0.43(0.36-0.50) | 80.42(65.27-95.46) | 102.91(88.55-122.20) | 0.60(0.50-0.71) | 127.70(102.73-155.27) | 83.31(68.75-100.65) | -1.47(-1.71--1.23) |
| Uruguay | 12.05(11.04-13.07) | 14.68(13.28-16.41) | 0.77(0.70-0.83) | 85.64(73.96-96.43) | 115.28(100.16-136.37) | 1.17(1.09-1.24) | 85.26(80.04-89.79) | 97.59(90.01-104.79) | 0.29(0.21-0.38) |
| Uzbekistan | 10.92(9.56-12.41) | 13.38(12.31-14.48) | 0.70(0.67-0.74) | 80.34(64.75-97.56) | 90.81(76.43-104.10) | 0.38(0.28-0.47) | 52.12(42.11-65.57) | 60.31(53.08-67.69) | 0.55(0.42-0.68) |
| Vanuatu | 12.94(11.68-14.58) | 12.96(11.64-14.80) | 0.06(0.02-0.09) | 86.52(72.17-103.06) | 91.01(75.90-109.91) | 0.25(0.18-0.31) | 111.85(91.45-135.72) | 102.20(83.03-120.89) | -0.34(-0.38--0.31) |
| Venezuela (Bolivarian Republic of) | 8.67(7.89-9.70) | 11.08(10.03-12.23) | 0.67(0.58-0.75) | 65.37(55.11-76.06) | 93.01(80.80-107.68) | 0.93(0.80-1.06) | 64.08(59.28-68.24) | 76.97(63.43-93.24) | 0.40(0.23-0.57) |
| Viet Nam | 10.35(9.15-11.45) | 13.77(12.33-15.61) | 0.96(0.93-0.99) | 71.57(58.54-86.10) | 103.23(87.00-124.63) | 1.24(1.19-1.29) | 78.73(66.21-94.86) | 96.09(81.43-112.83) | 0.76(0.69-0.82) |
| Yemen | 7.72(6.96-8.57) | 10.20(9.08-11.30) | 1.02(0.96-1.08) | 50.13(41.71-59.08) | 74.38(62.02-88.47) | 1.41(1.33-1.50) | 74.70(57.23-99.23) | 82.23(62.19-109.94) | 0.35(0.30-0.40) |
| Zambia | 8.68(7.86-9.51) | 9.17(8.15-10.20) | 0.19(0.15-0.23) | 52.71(43.47-62.89) | 60.69(49.62-71.69) | 0.49(0.39-0.58) | 67.10(53.75-82.90) | 69.79(53.94-91.64) | -0.06(-0.17-0.04) |
| Zimbabwe | 10.49(9.39-11.76) | 11.25(10.01-12.52) | 0.13(0.10-0.16) | 64.04(52.50-76.61) | 70.00(56.40-82.97) | 0.00(-0.07-0.08) | 82.84(68.68-95.91) | 96.85(79.51-117.42) | 0.69(0.44-0.94) |
